# Supplementary material for: Synthesis and In Vitro Anti-Toxoplasma gondii Activity of Novel Thiazolidin-4-one Derivatives
Source: Molecules. 2019 Aug 21;24(17):3029. doi: 10.3390/molecules24173029 (PMC6749389; doi:10.3390/molecules24173029)
Supplement: Supplementary file 1 [file molecules-24-03029-s001.pdf]

Supplementary material for:

Synthesis and *in vitro* anti-*Toxoplasma gondii* activity of novel thiazolidin-4-one derivatives

Nazar Trotsko[1]*, Adrian Bekier[2], Agata Paneth[1], Monika Wujec[1] and Katarzyna Dzitko[2]*

[1] Department of Organic Chemistry, Faculty of Pharmacy with Medical Analytics Division, Medical University of Lublin, 4A Chodźki, 20-093 Lublin, Poland; nazar.trotsko@umlub.pl (N.T.); agata.paneth@umlub.pl (A.P.); monika.wujec@umlub.pl (M.W.).

[2] Department of Immunoparasitology, Faculty of Biology and Environmental Protection, University of Lodz, Banacha 12/16, 90-237 Lodz, Poland; katarzyna.dzitko@biol.uni.lodz.pl (K.D.); adrian.bekier@unilodz.eu (A.B.).

* Correspondence: nazar.trotsko@umlub.pl and katarzyna.dzitko@biol.uni.lodz.pl

Contents:

1. ^{1}H NMR and ^{13}C NMR spectra for thiazolidin-4-ones (**42-73**) …………………..2
2. ^{1}H NMR and ^{13}C NMR spectra for (4-oxothiazolidin-5-ylo)acetic acid derivatives (**74-88**)………………………………………………………..58
3. ^{1}H NMR and ^{13}C NMR spectra for (4-oxothiazolidin-5-ylidene)acetic acid derivatives (**89-104**)…………………………………………………….…86

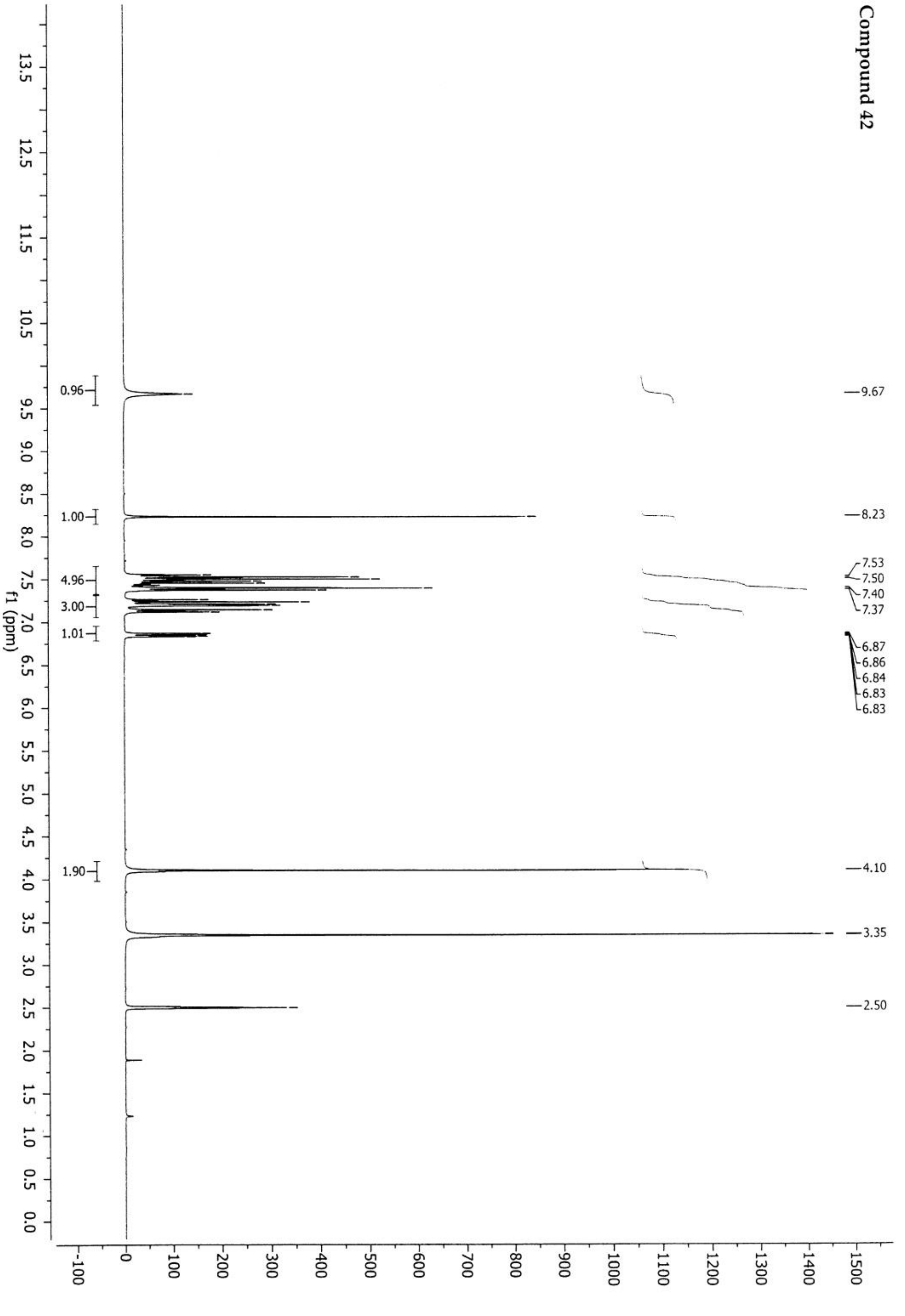

Fig. S1. ^{1}H NMR spectrum for compound **42**

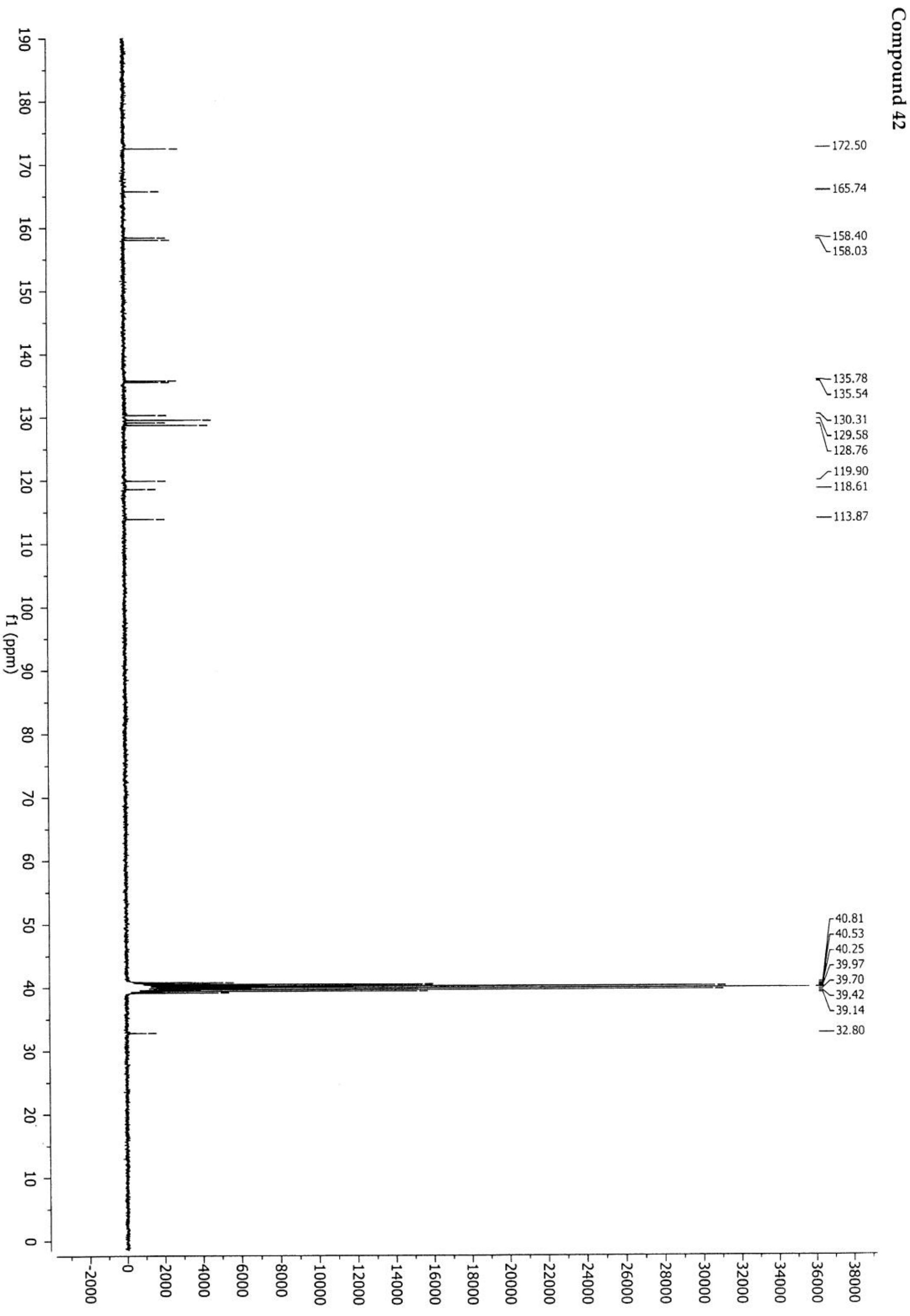

Fig. S2. ^{13}C NMR spectrum for compound **42**

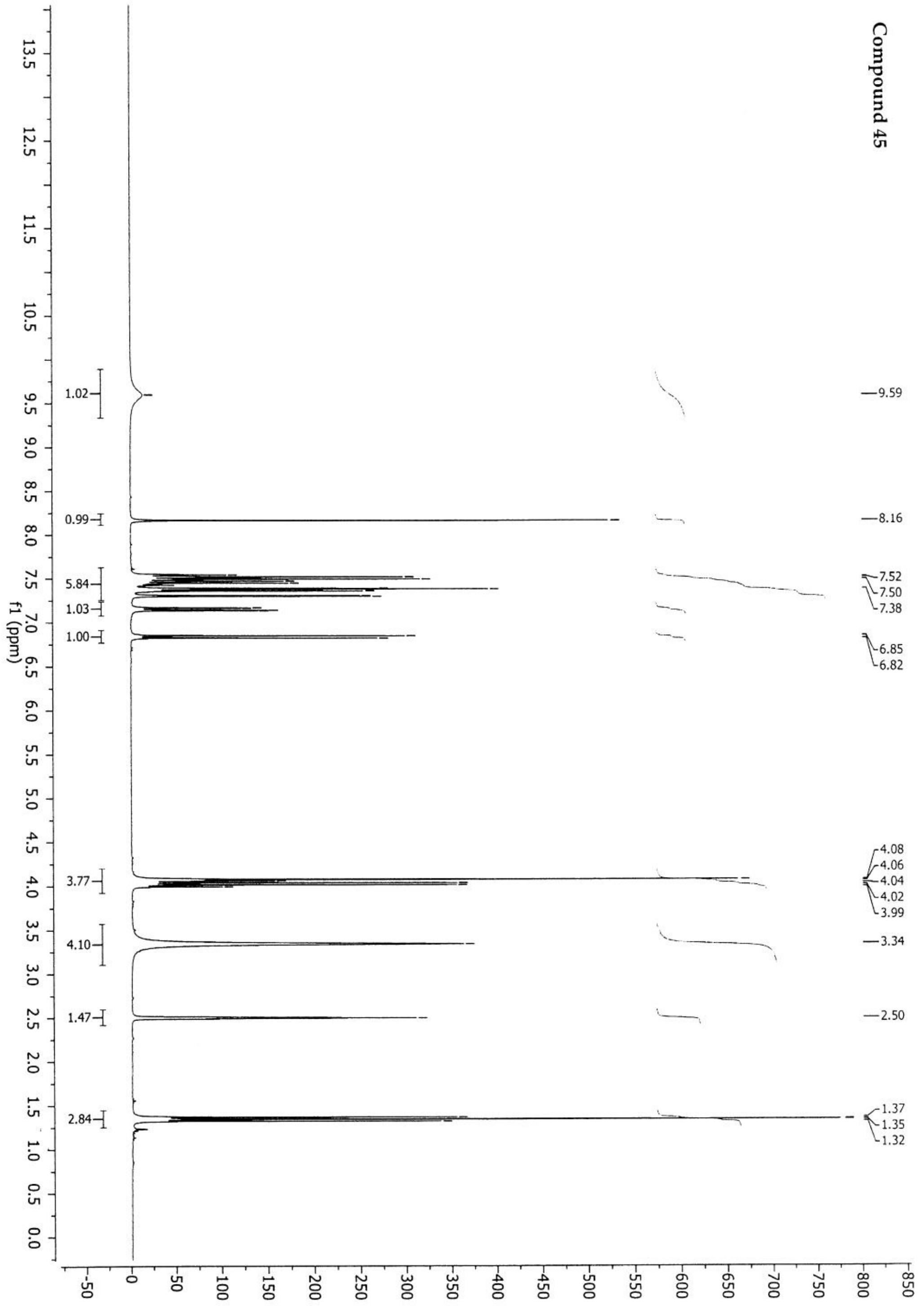

Fig. S3. ¹H NMR spectrum for compound **45**

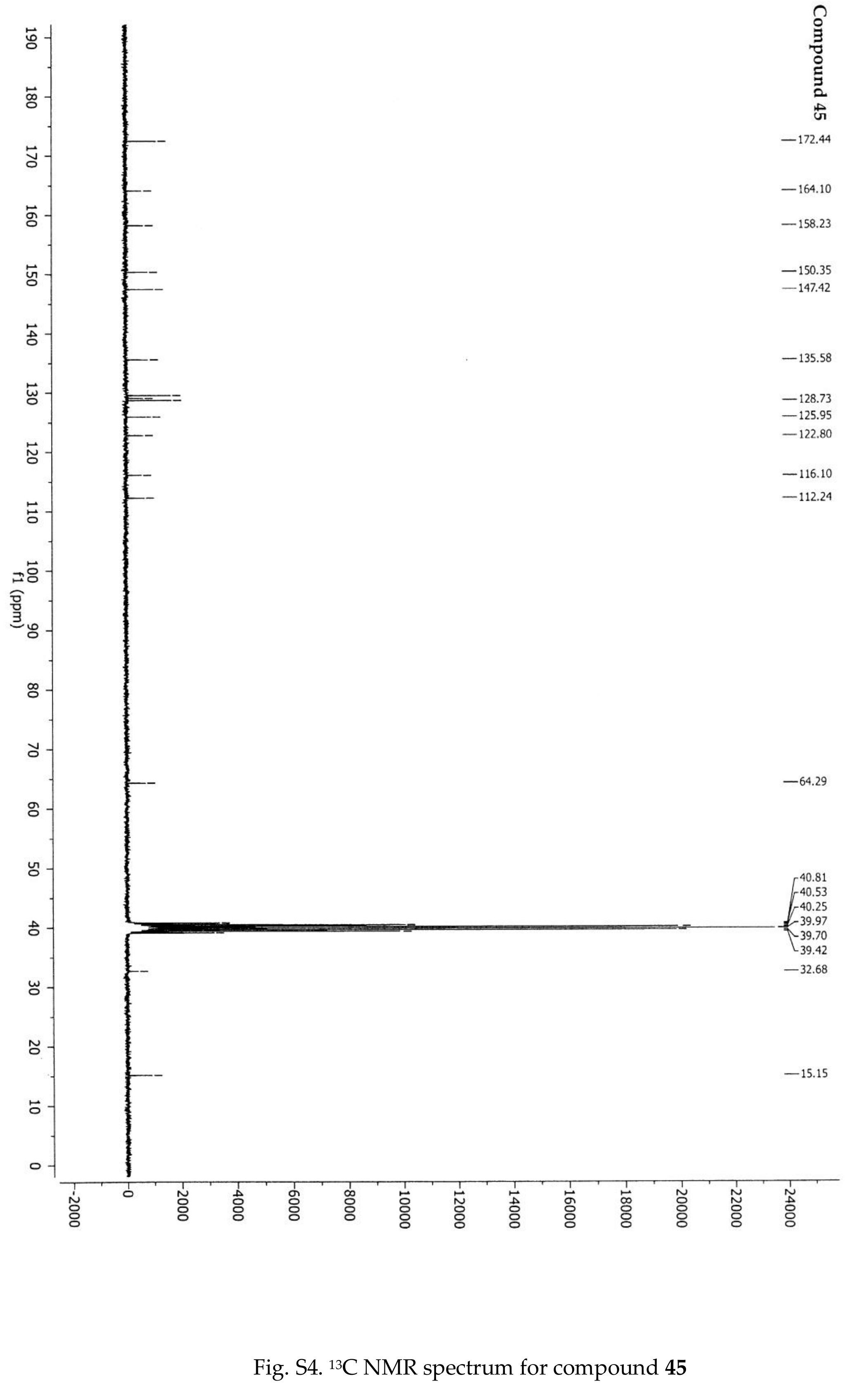

Fig. S4. ^{13}C NMR spectrum for compound **45**

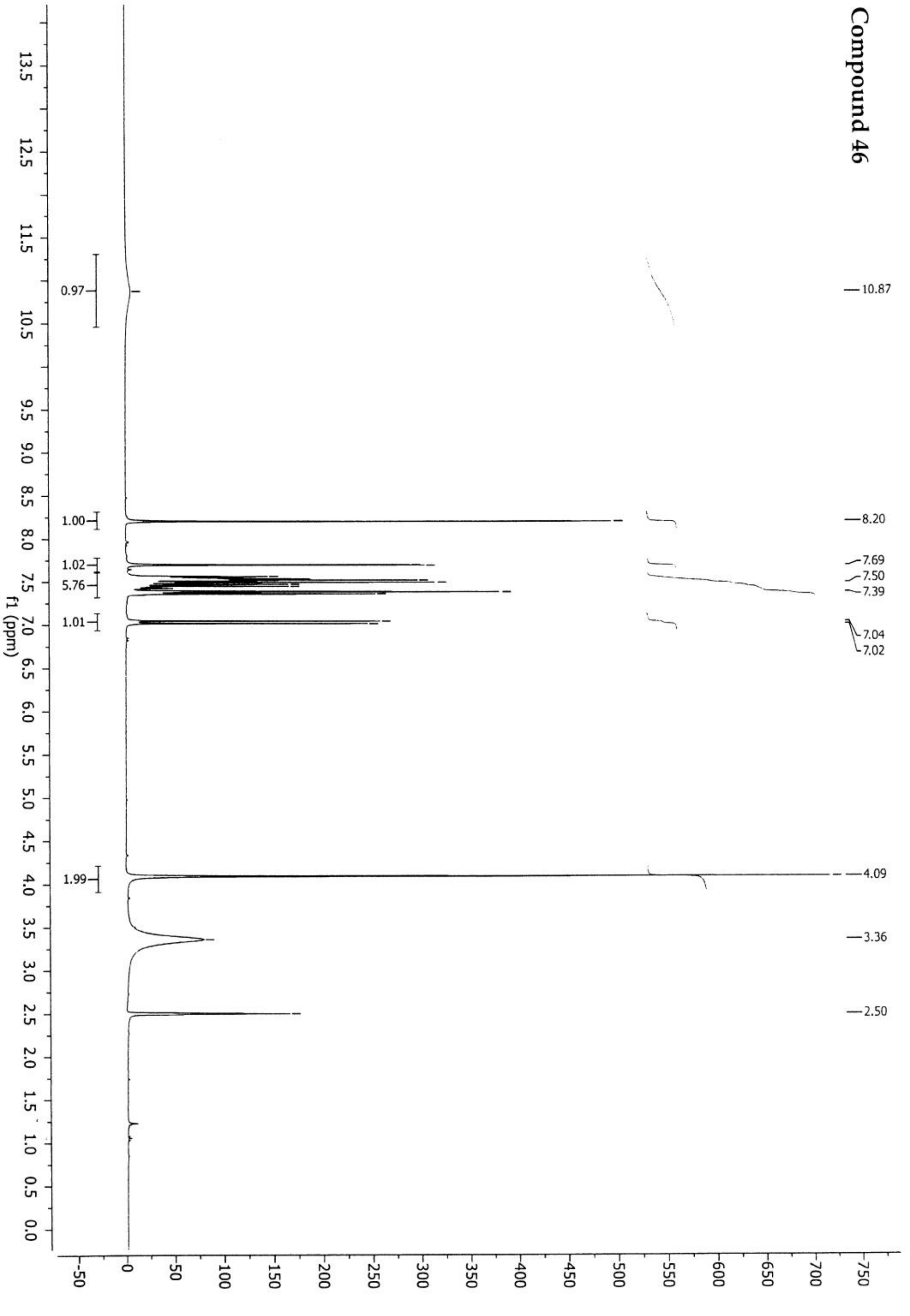

Fig. S5. ^{1}H NMR spectrum for compound **46**

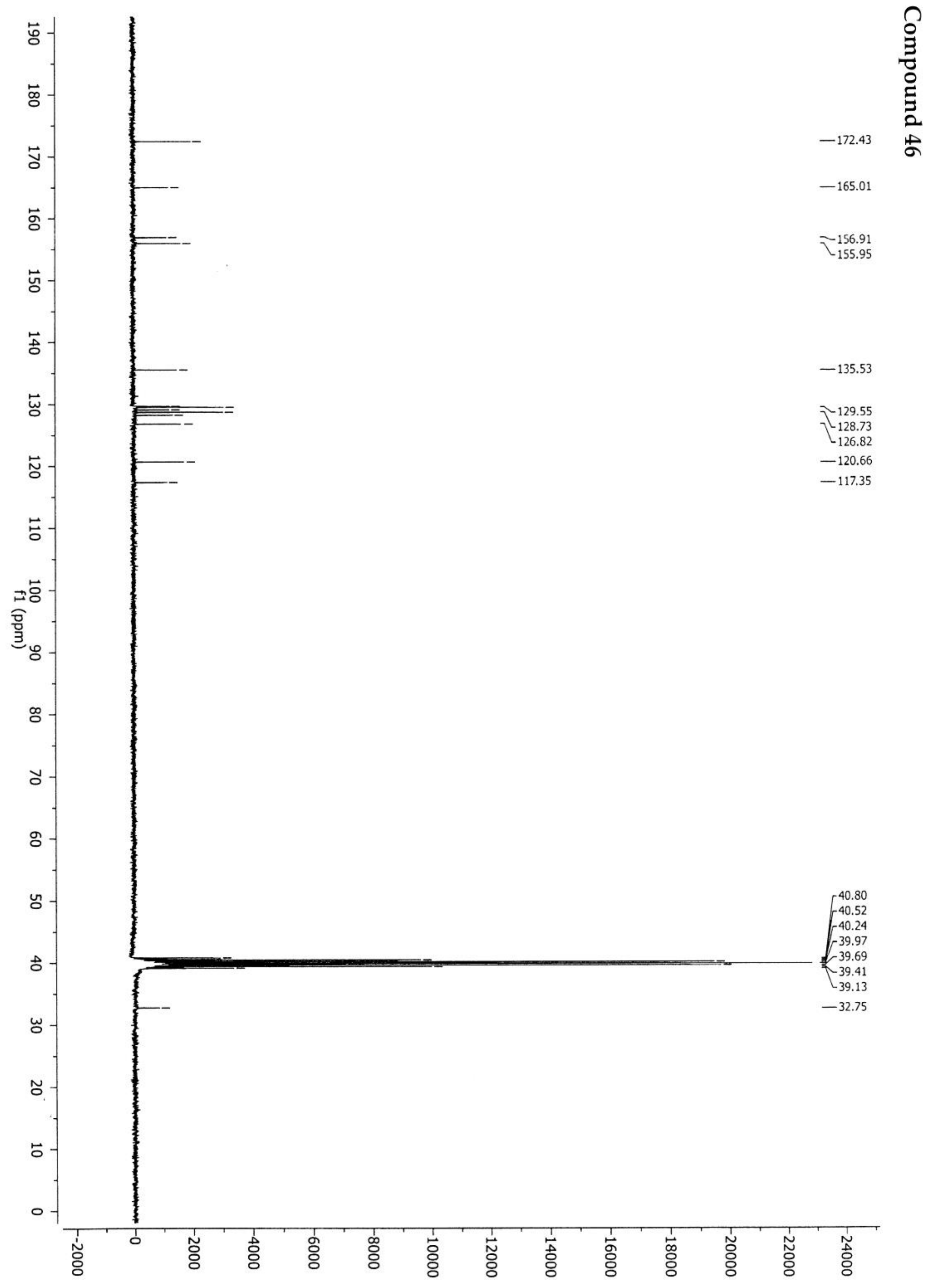

Fig. S6. ^{13}C NMR spectrum for compound **46**

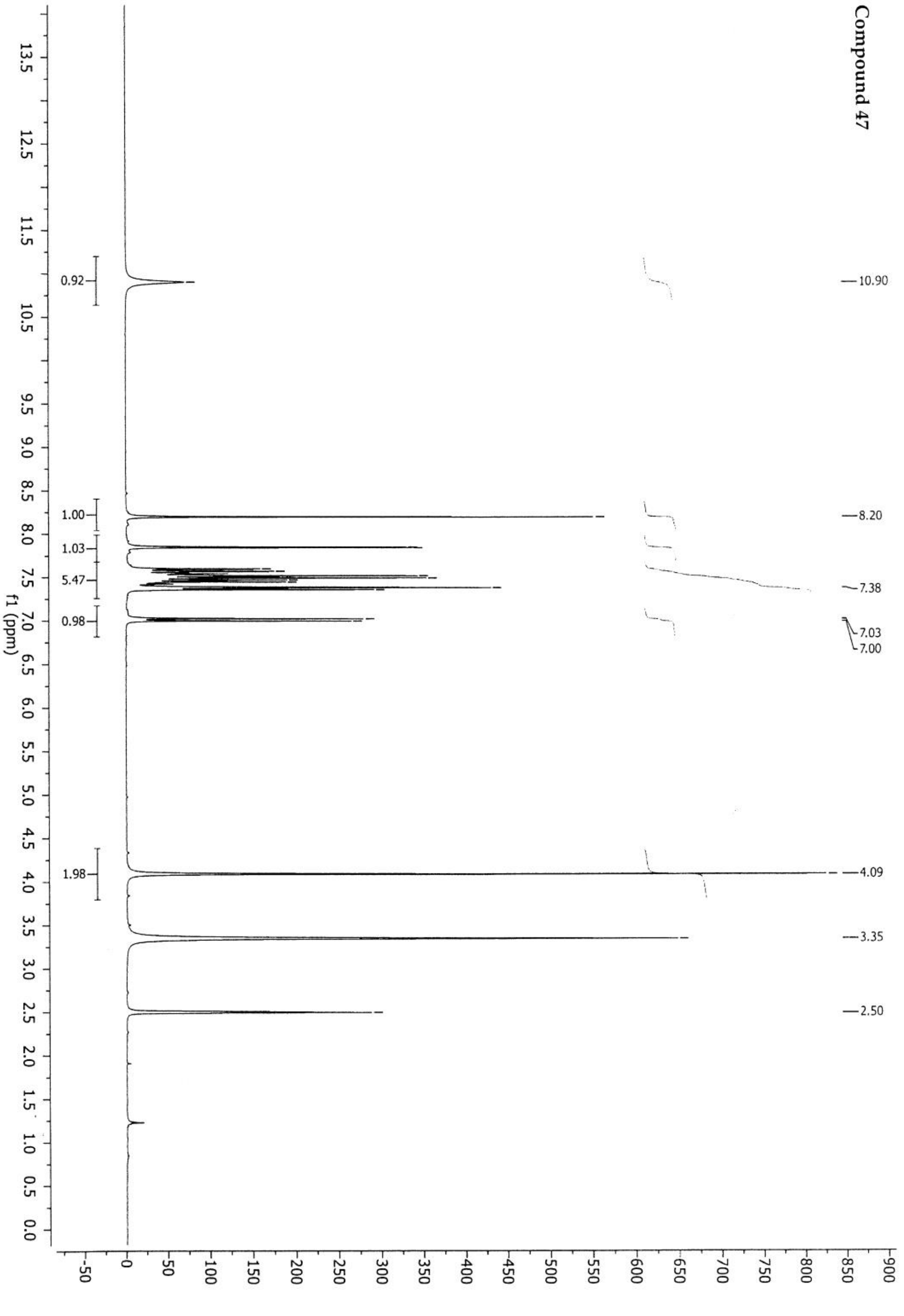

Fig. S7. ^{1}H NMR spectrum for compound **47**

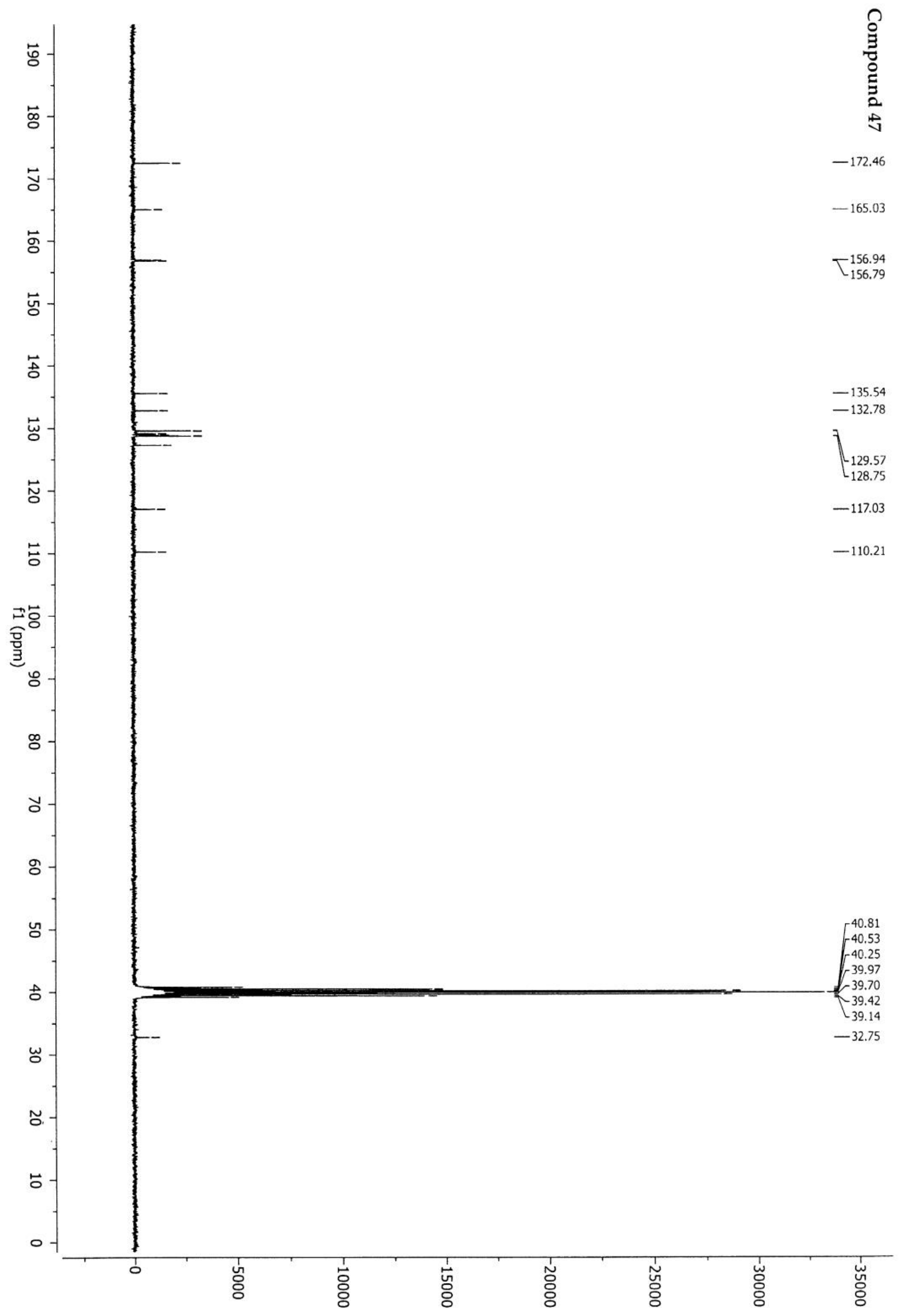

Fig. S8. ^{13}C NMR spectrum for compound **47**

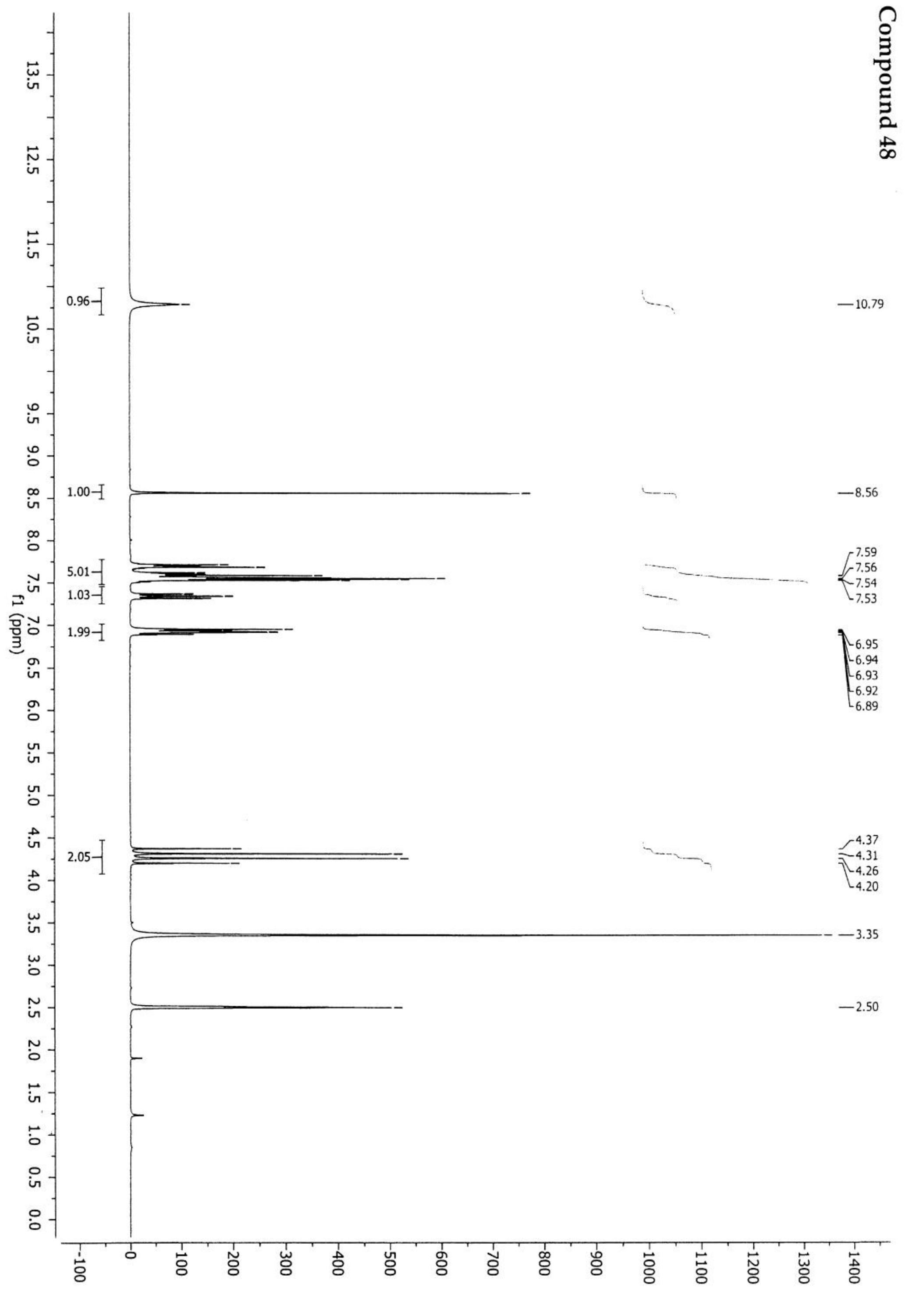

Fig. S9. ^{1}H NMR spectrum for compound **48**

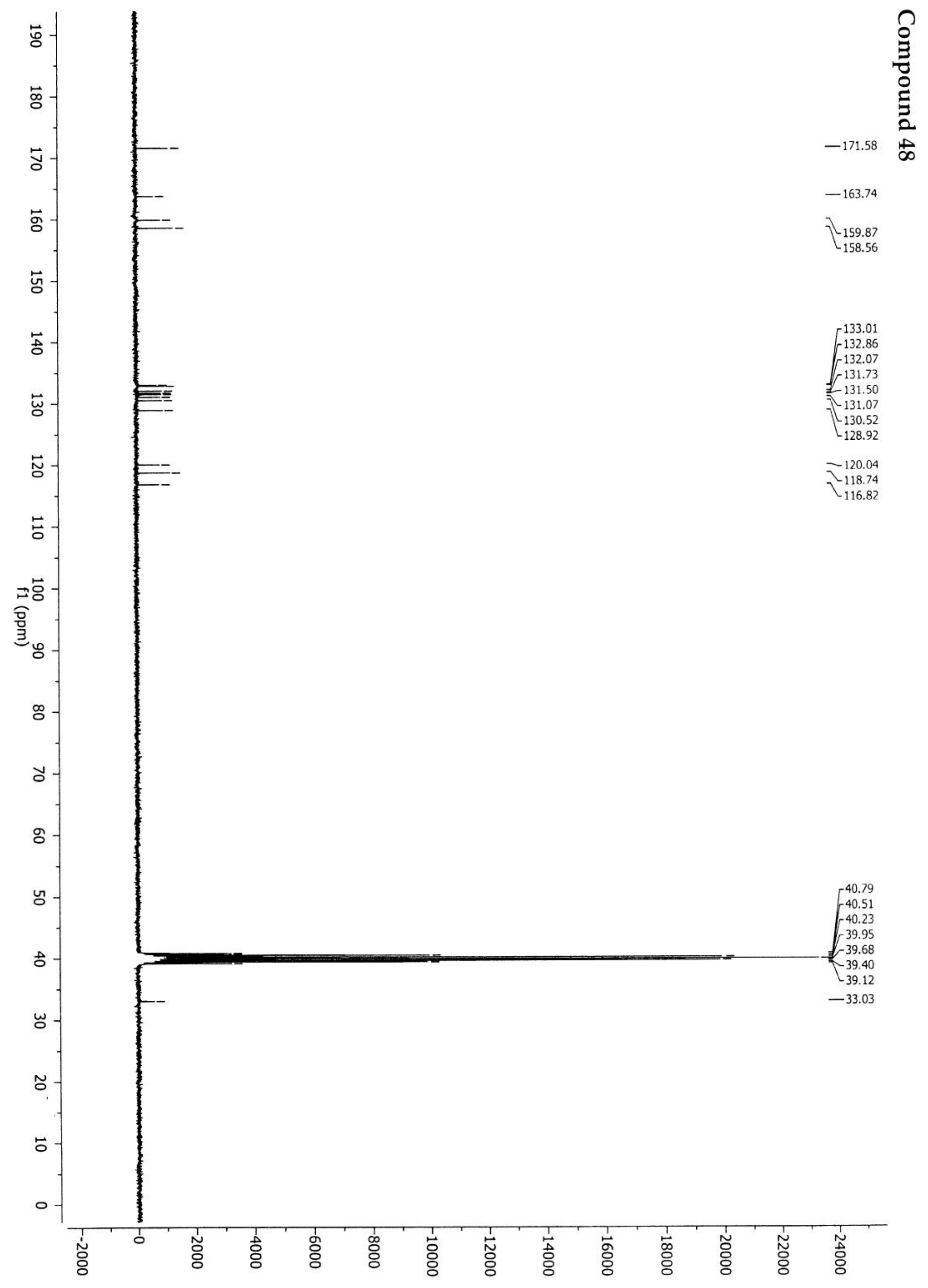

Fig. S10. ^{13}C NMR spectrum for compound **48**

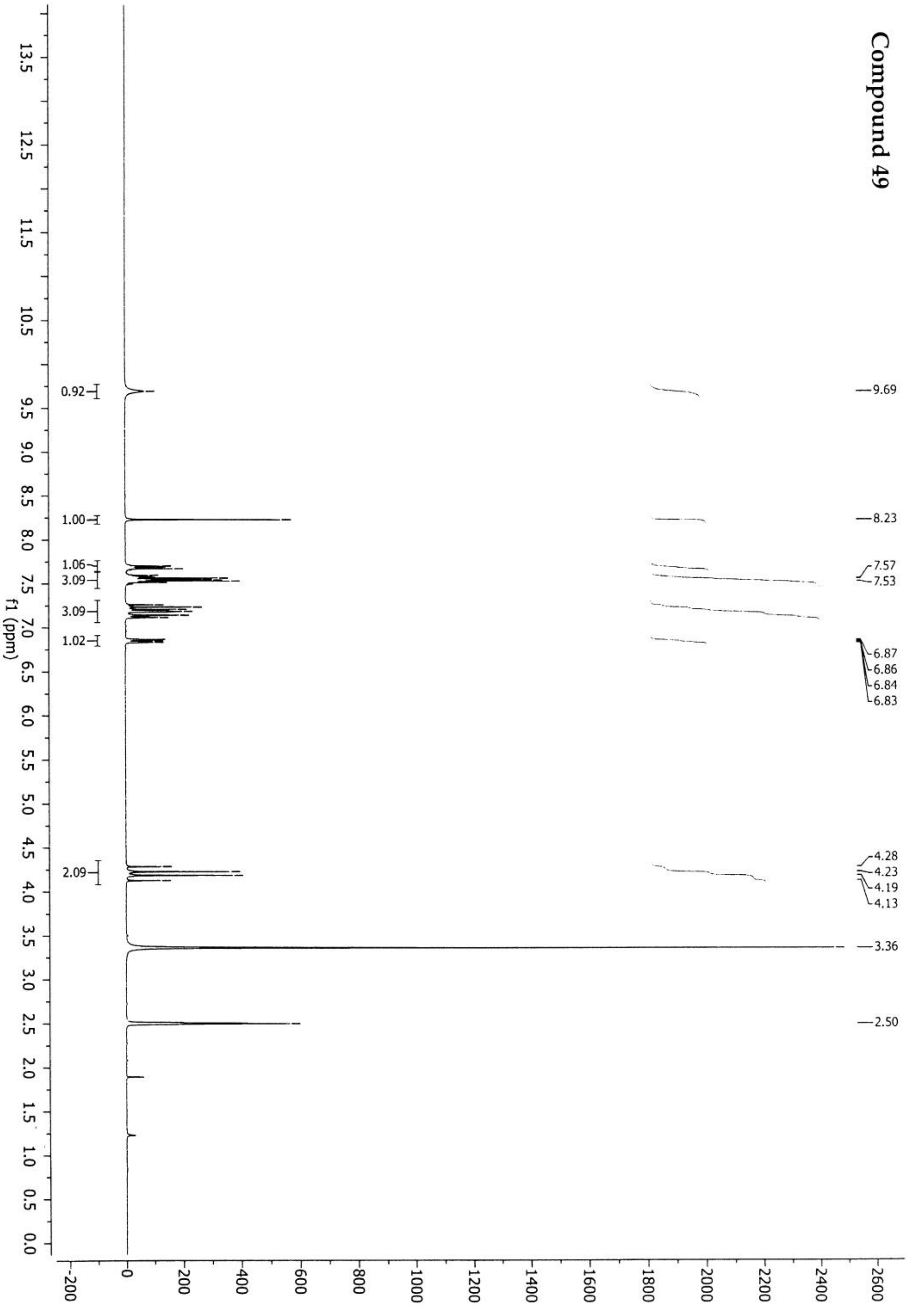

Fig. S11. ¹H NMR spectrum for compound **49**

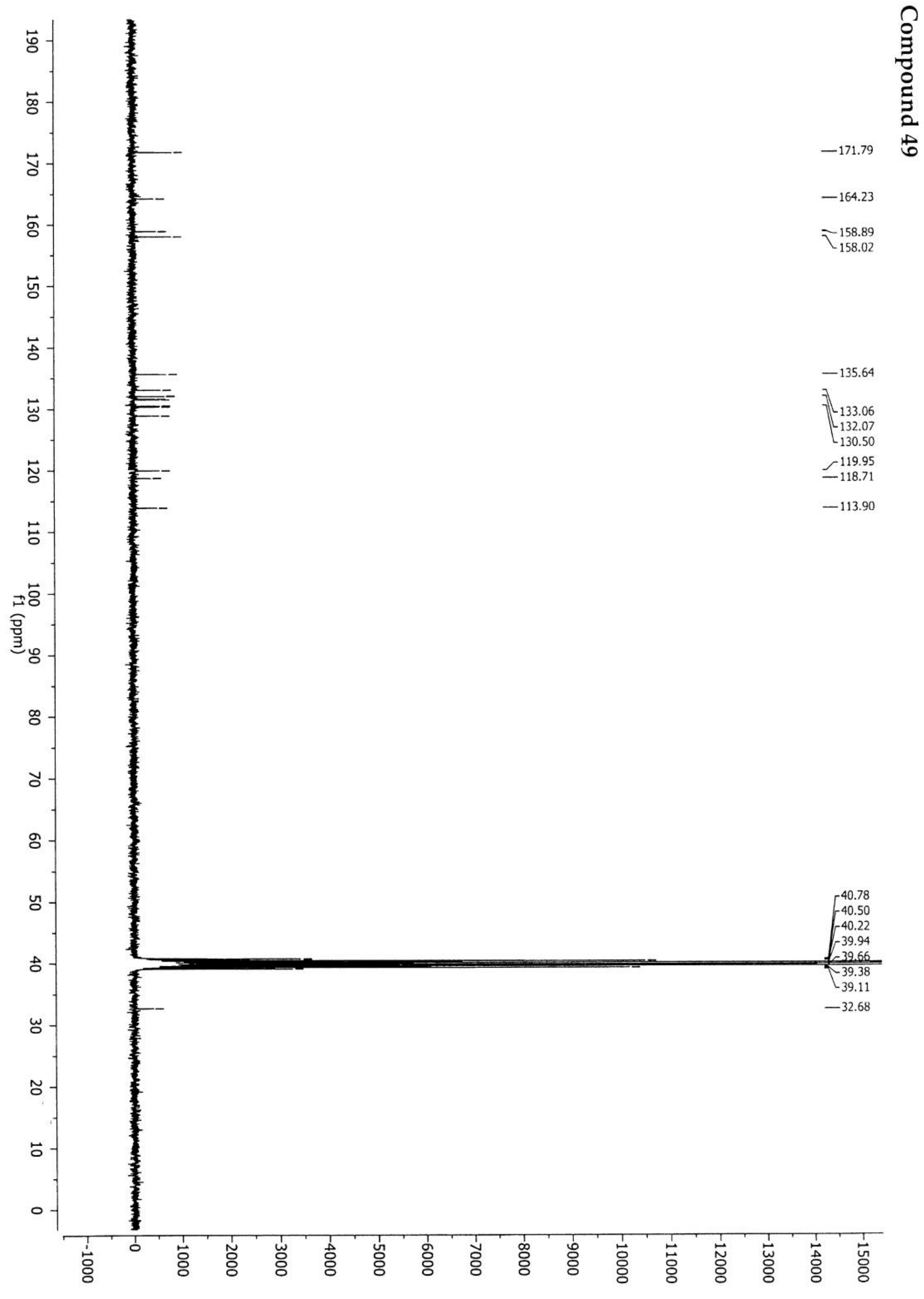

Fig. S12. ^{13}C NMR spectrum for compound **49**

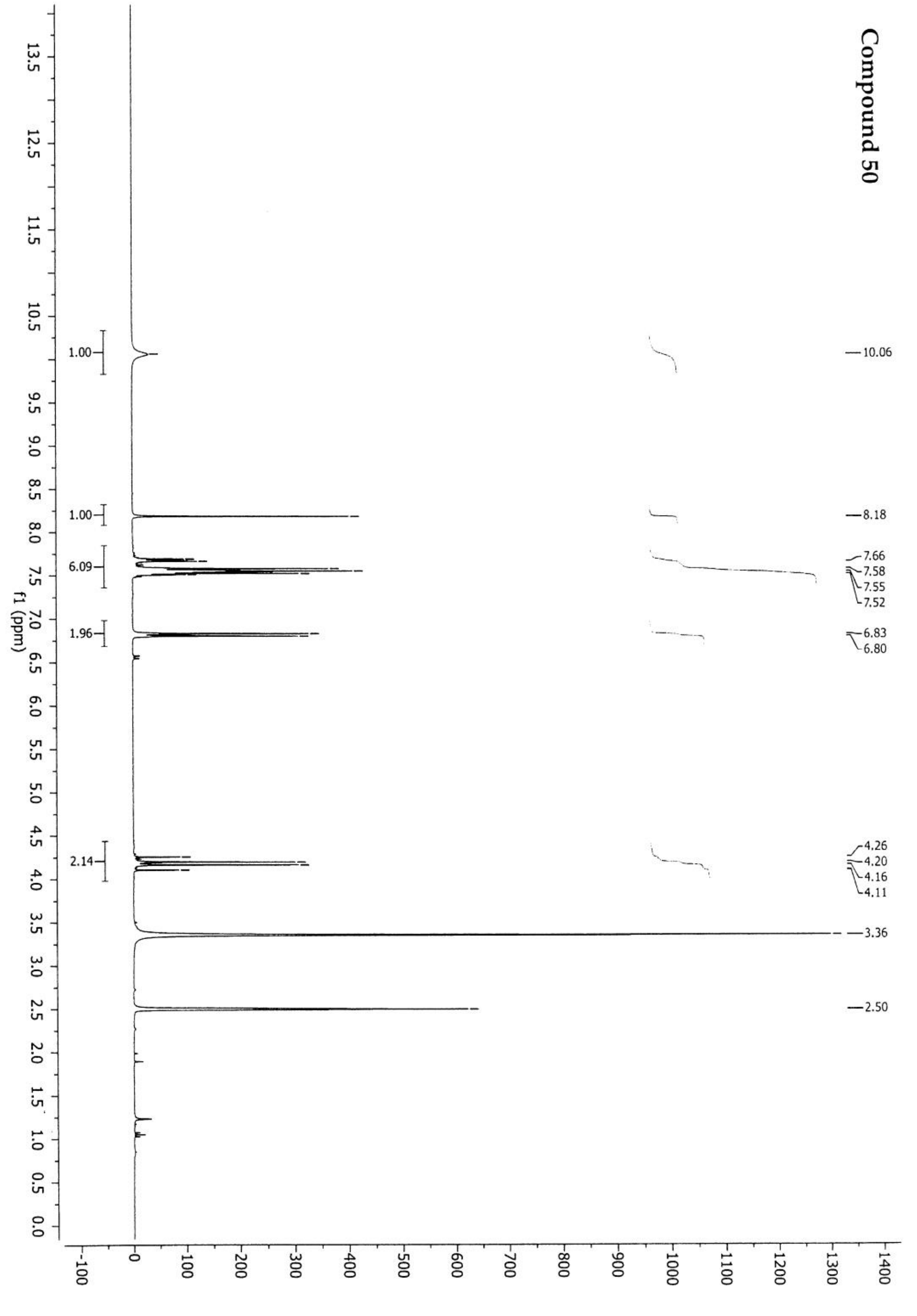

Fig. S13. ^{1}H NMR spectrum for compound **50**

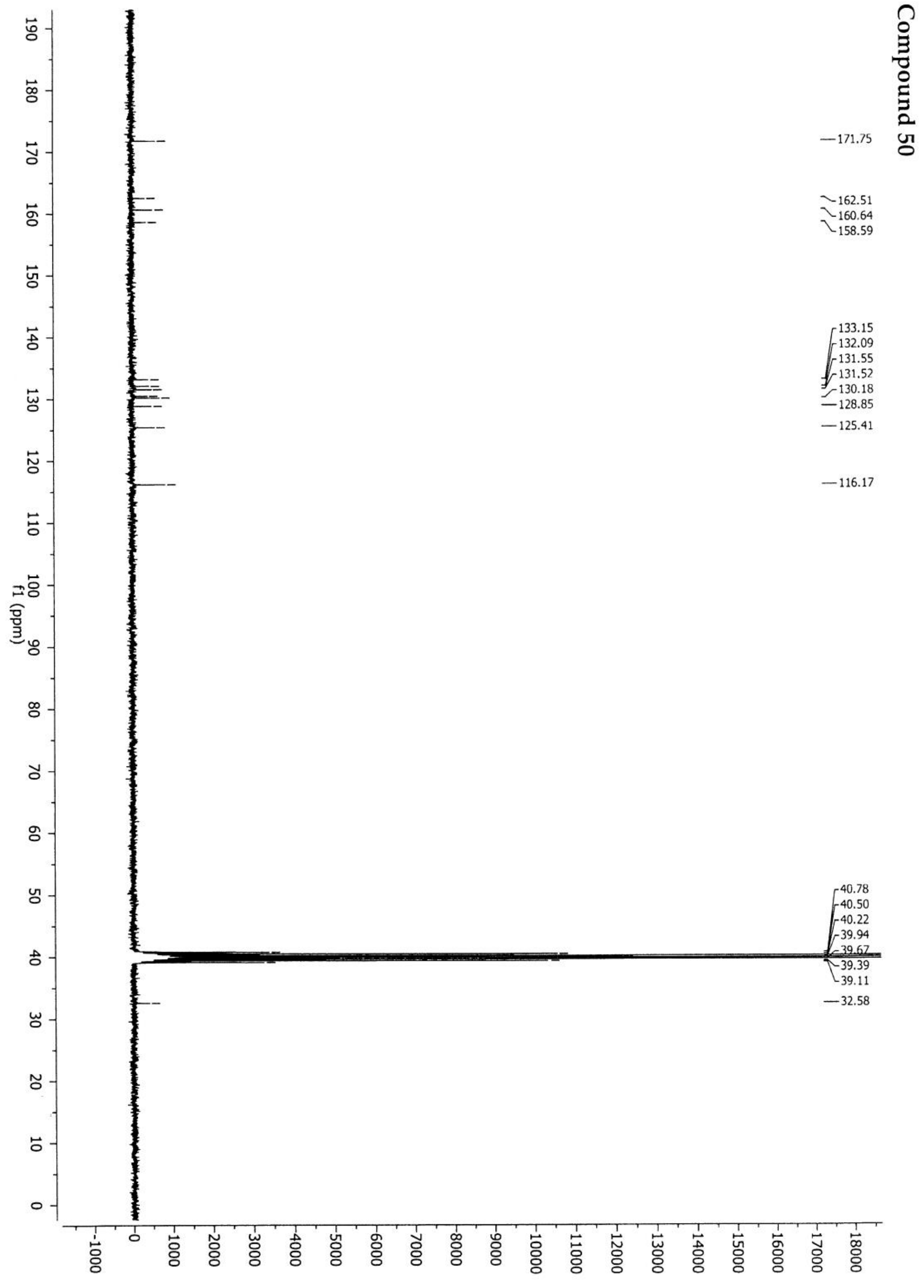

Fig. S14. ^{13}C NMR spectrum for compound **50**

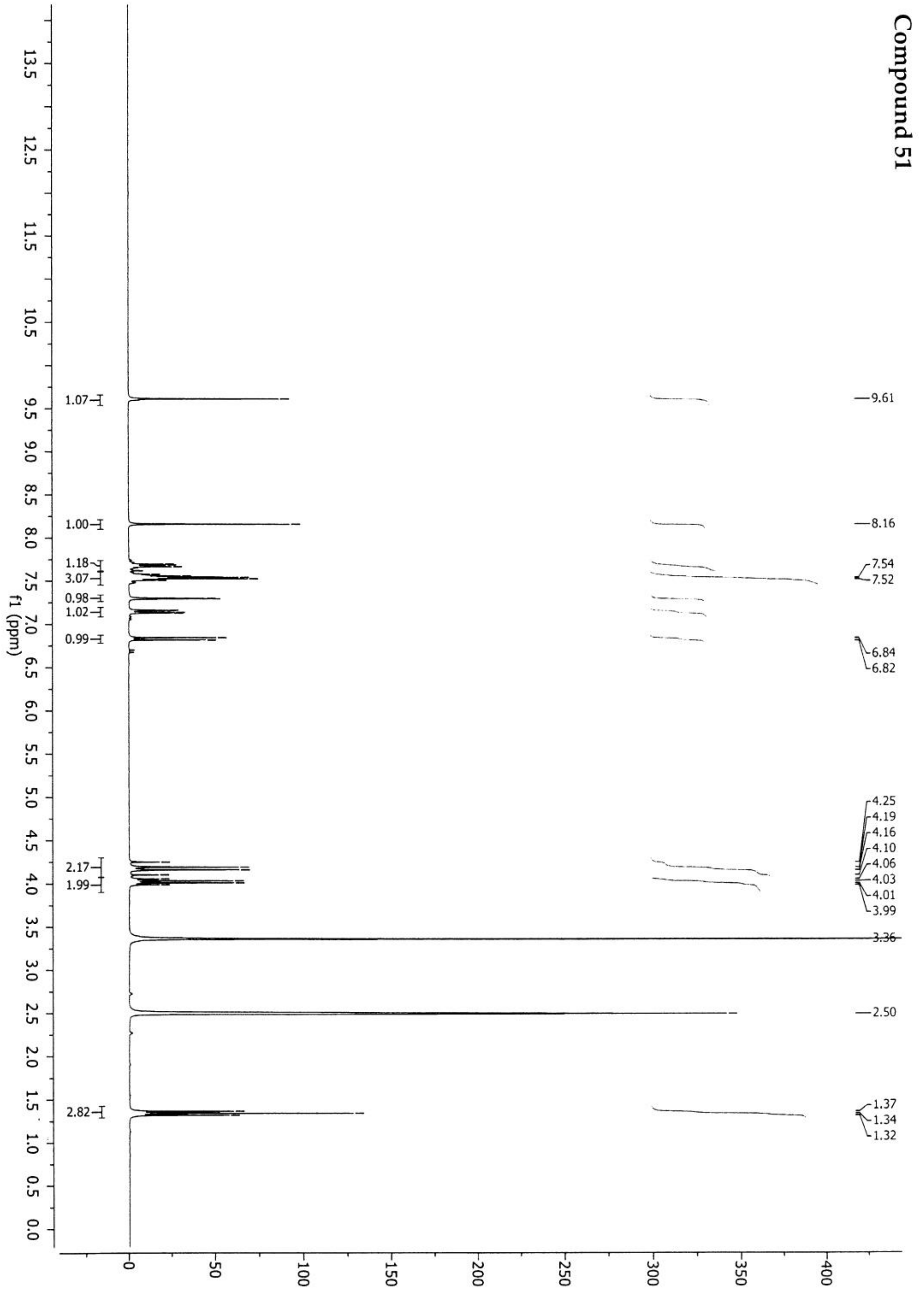

Fig. S15. ^{1}H NMR spectrum for compound **51**

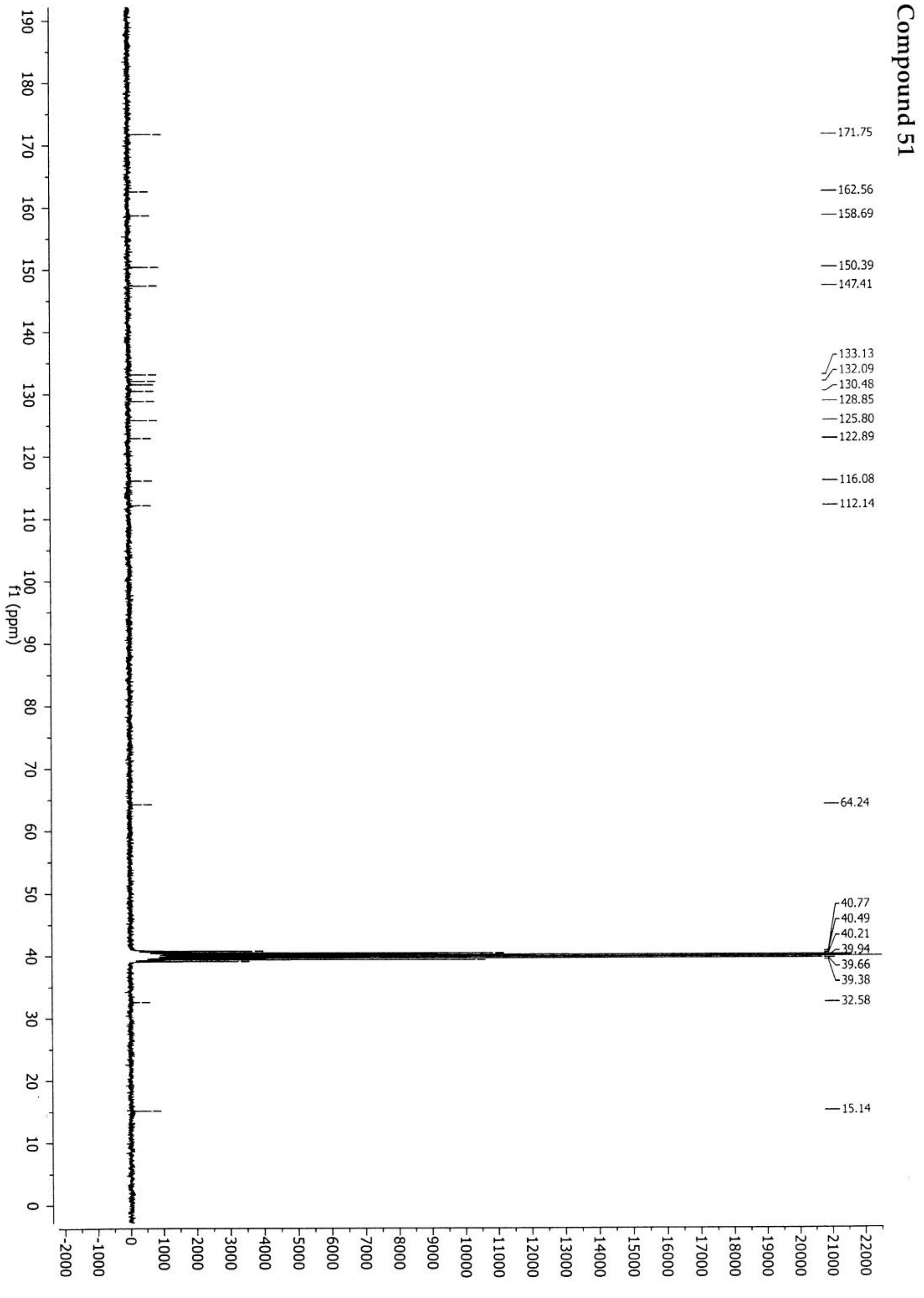

Fig. S16. ^{13}C NMR spectrum for compound **51**

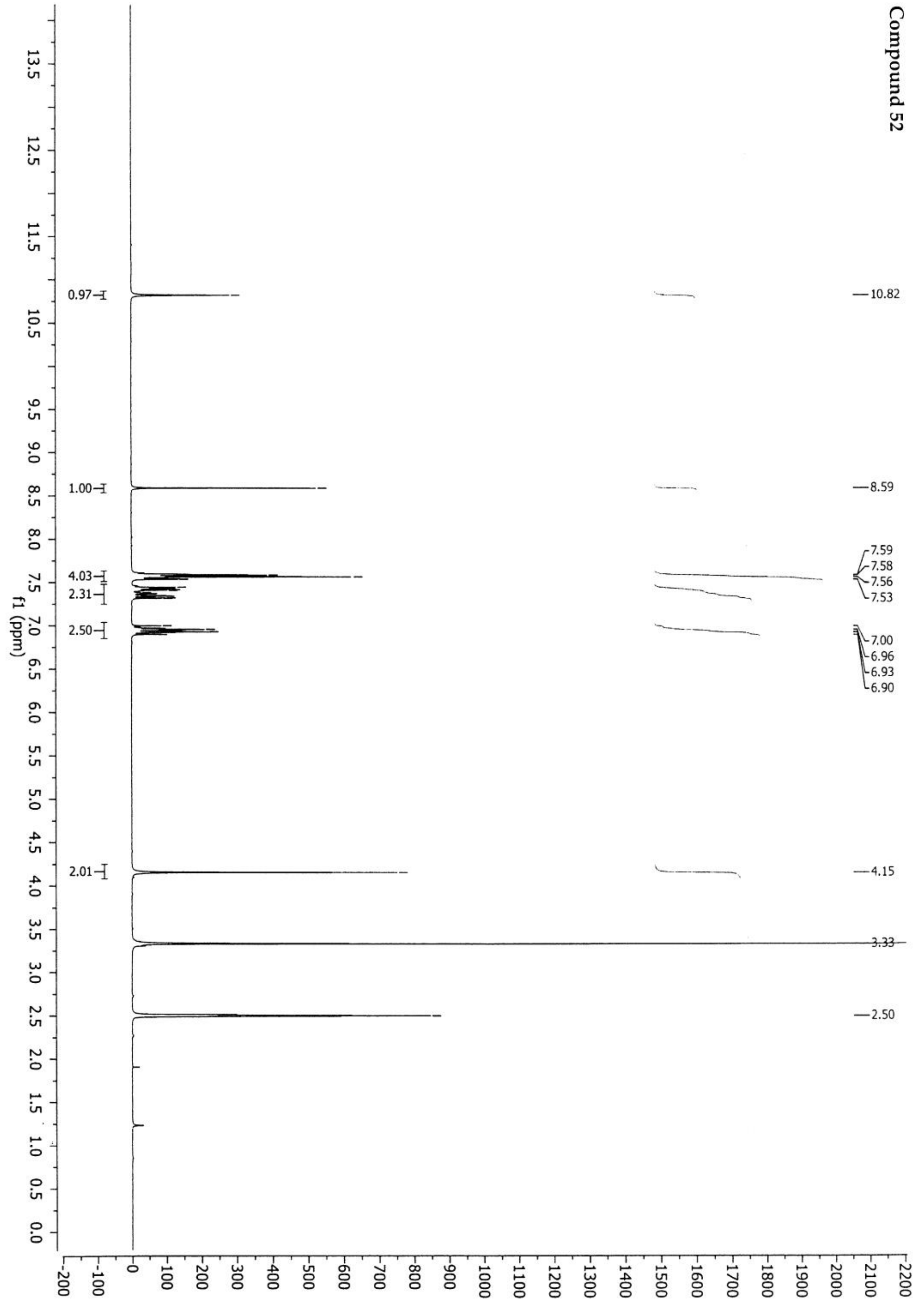

Fig. S17. 1H NMR spectrum for compound **52**

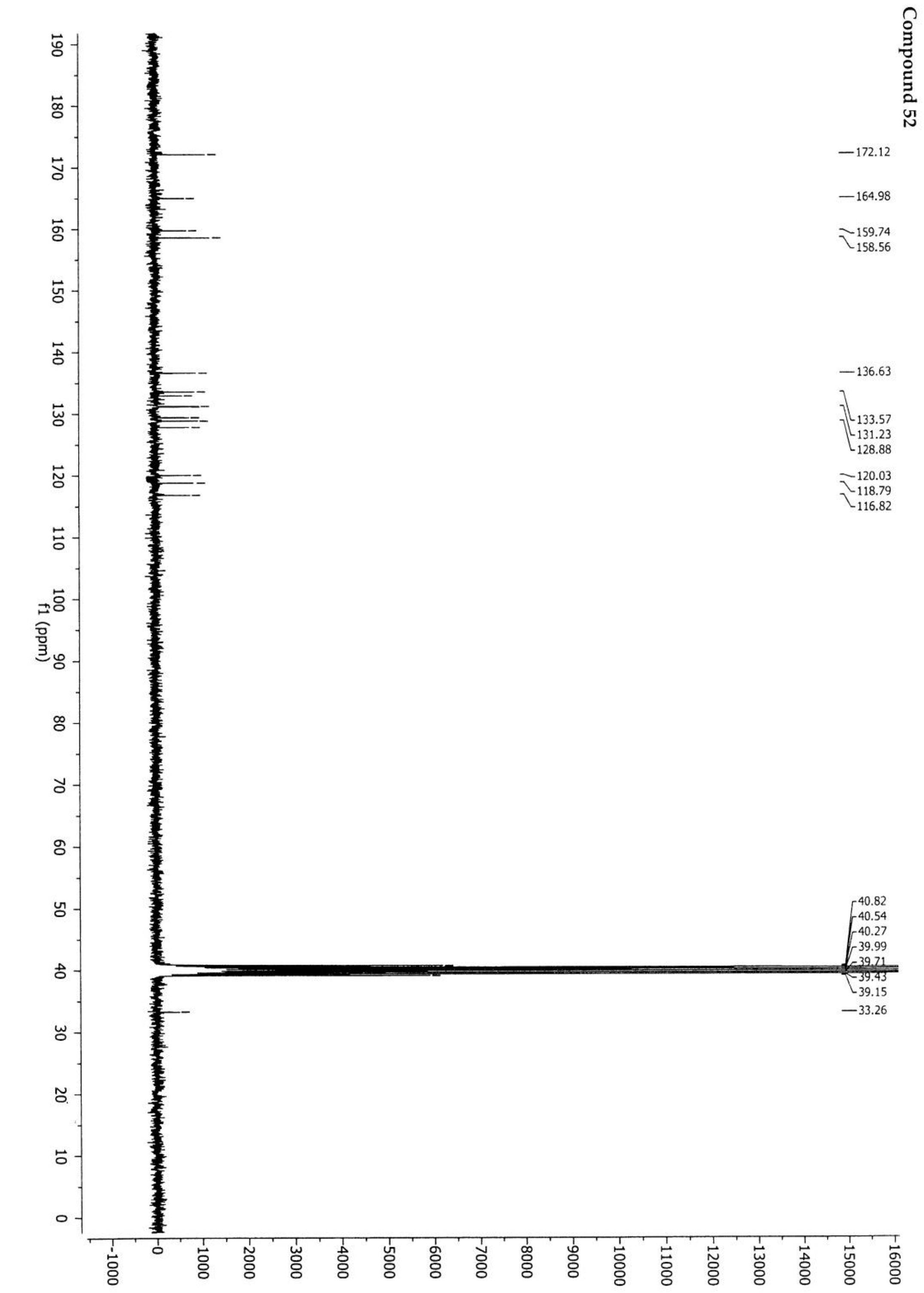

Fig. S18. ^{13}C NMR spectrum for compound **52**

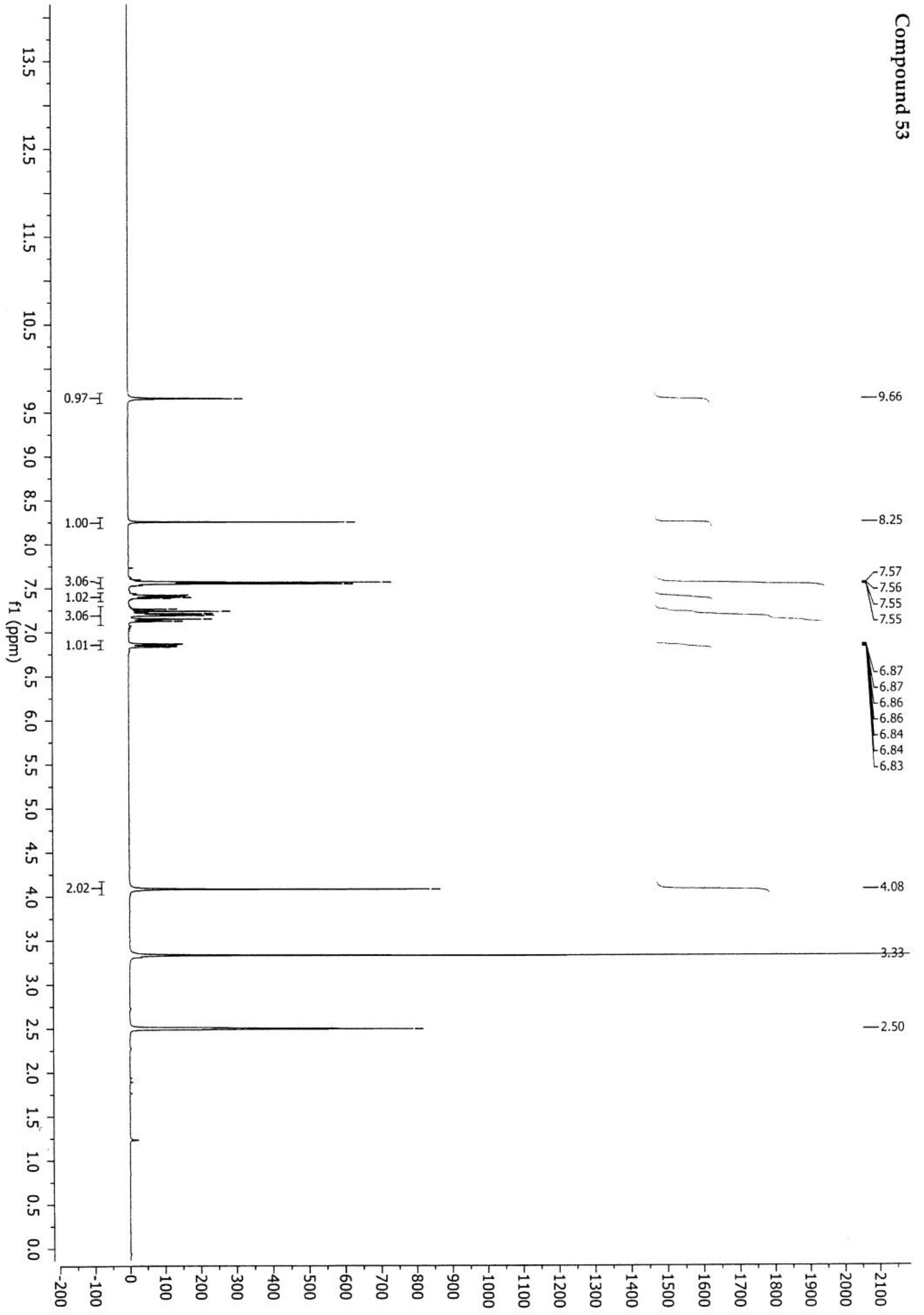

Fig. S19. ^{1}H NMR spectrum for compound **53**

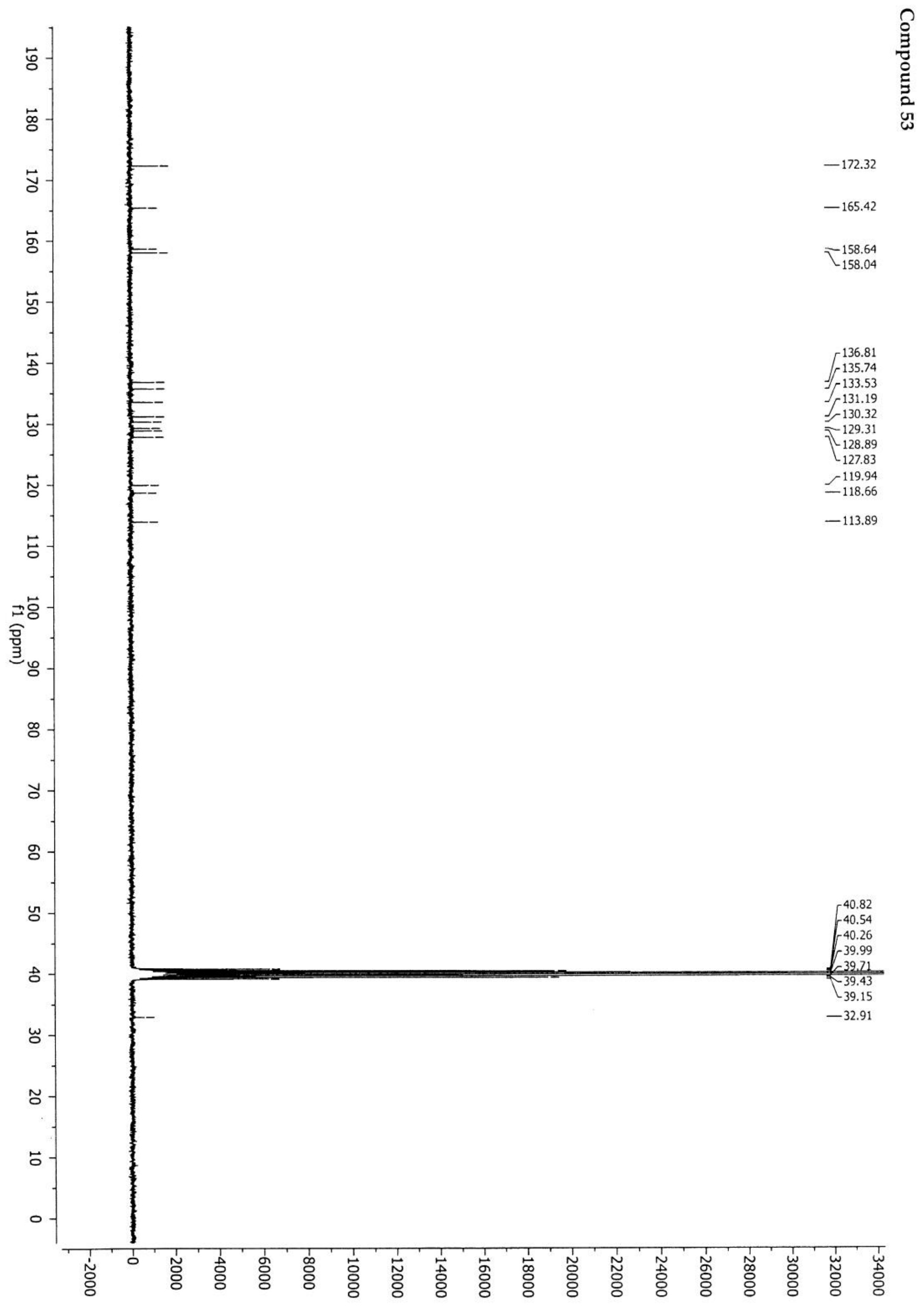

Fig. S20. ^{13}C NMR spectrum for compound **53**

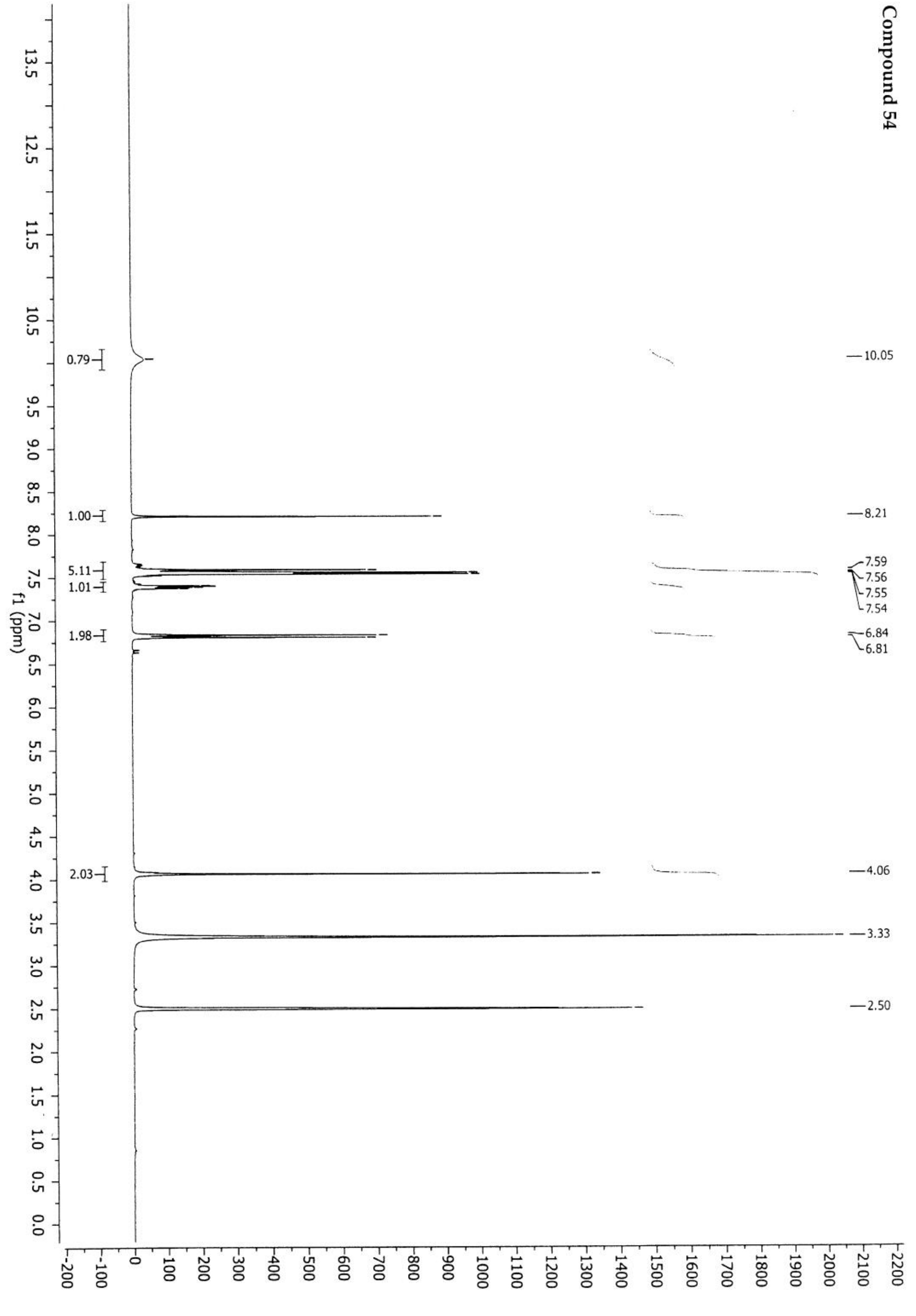

Fig. S21. ¹H NMR spectrum for compound **54**

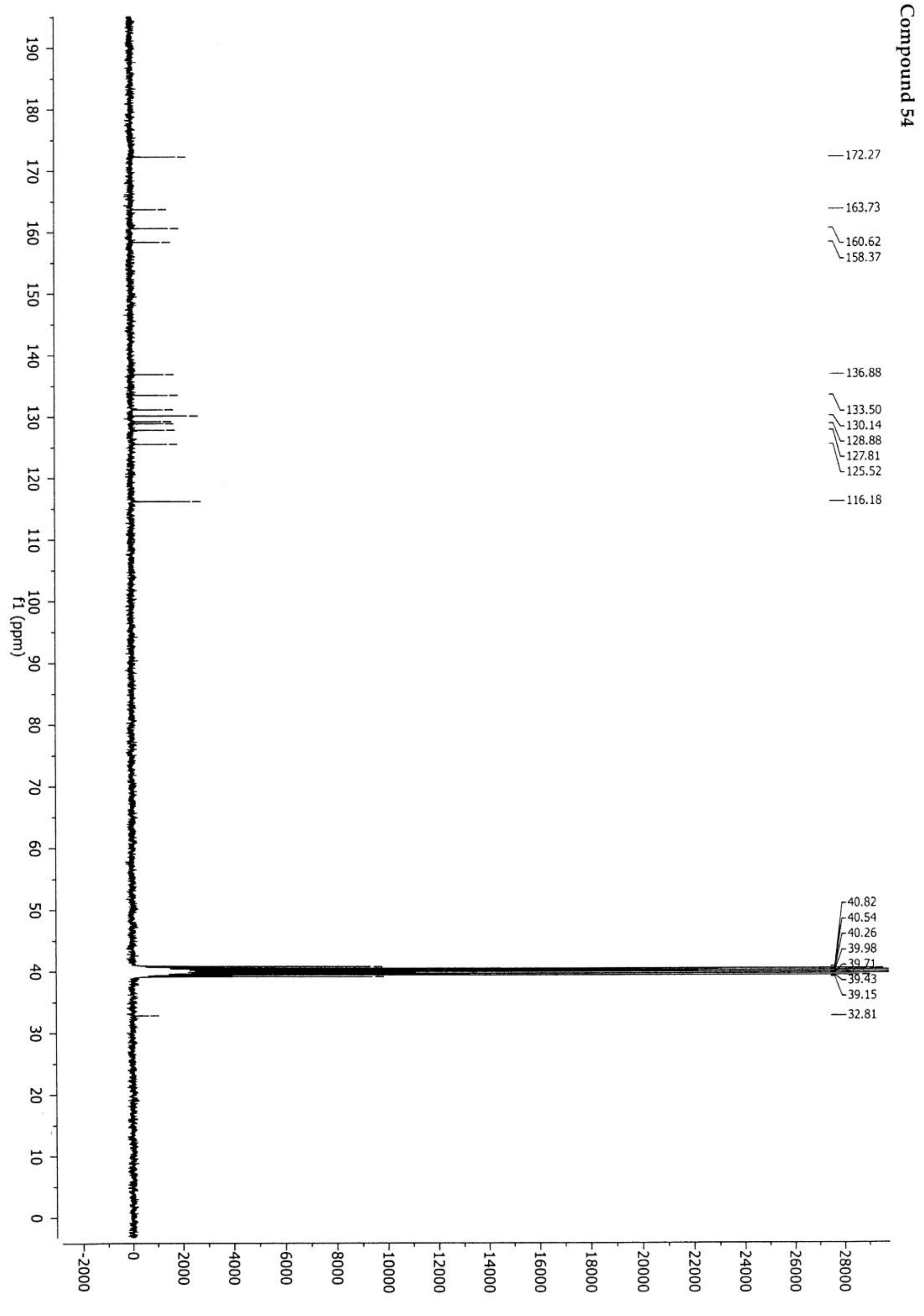

Fig. S22. ^{13}C NMR spectrum for compound **54**

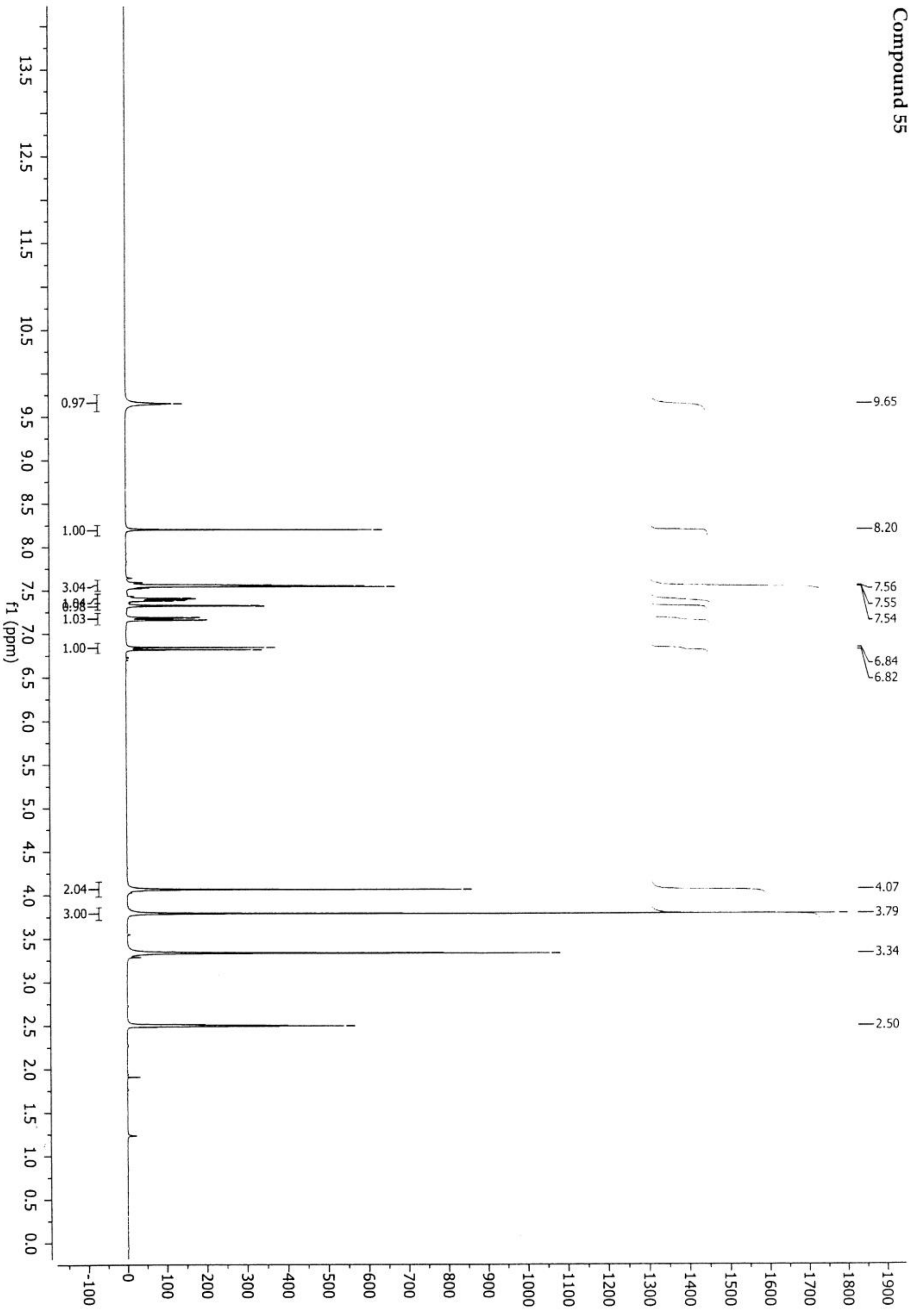

Fig. S23. ^{1}H NMR spectrum for compound **55**

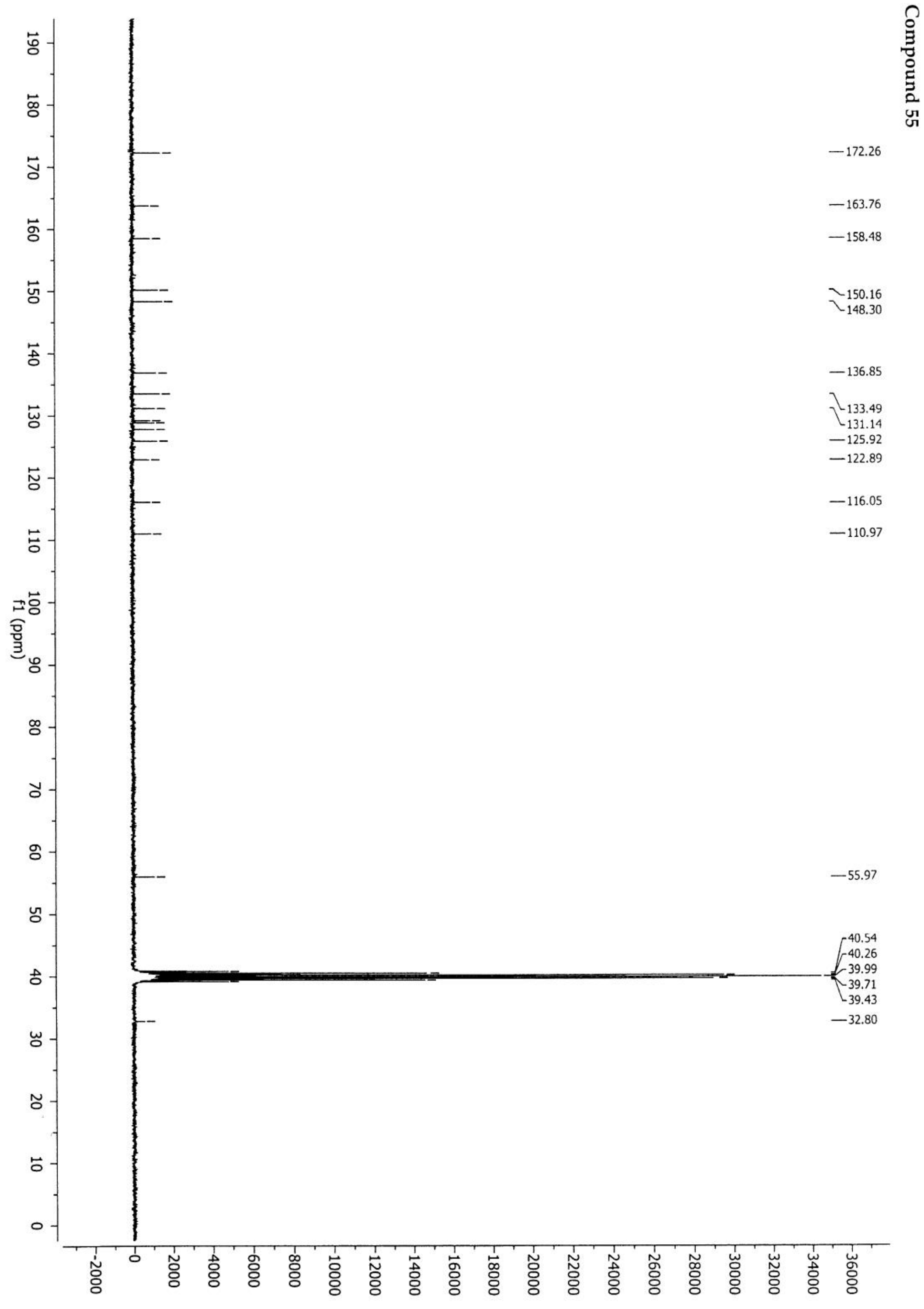

Fig. S24. ^{13}C NMR spectrum for compound **55**

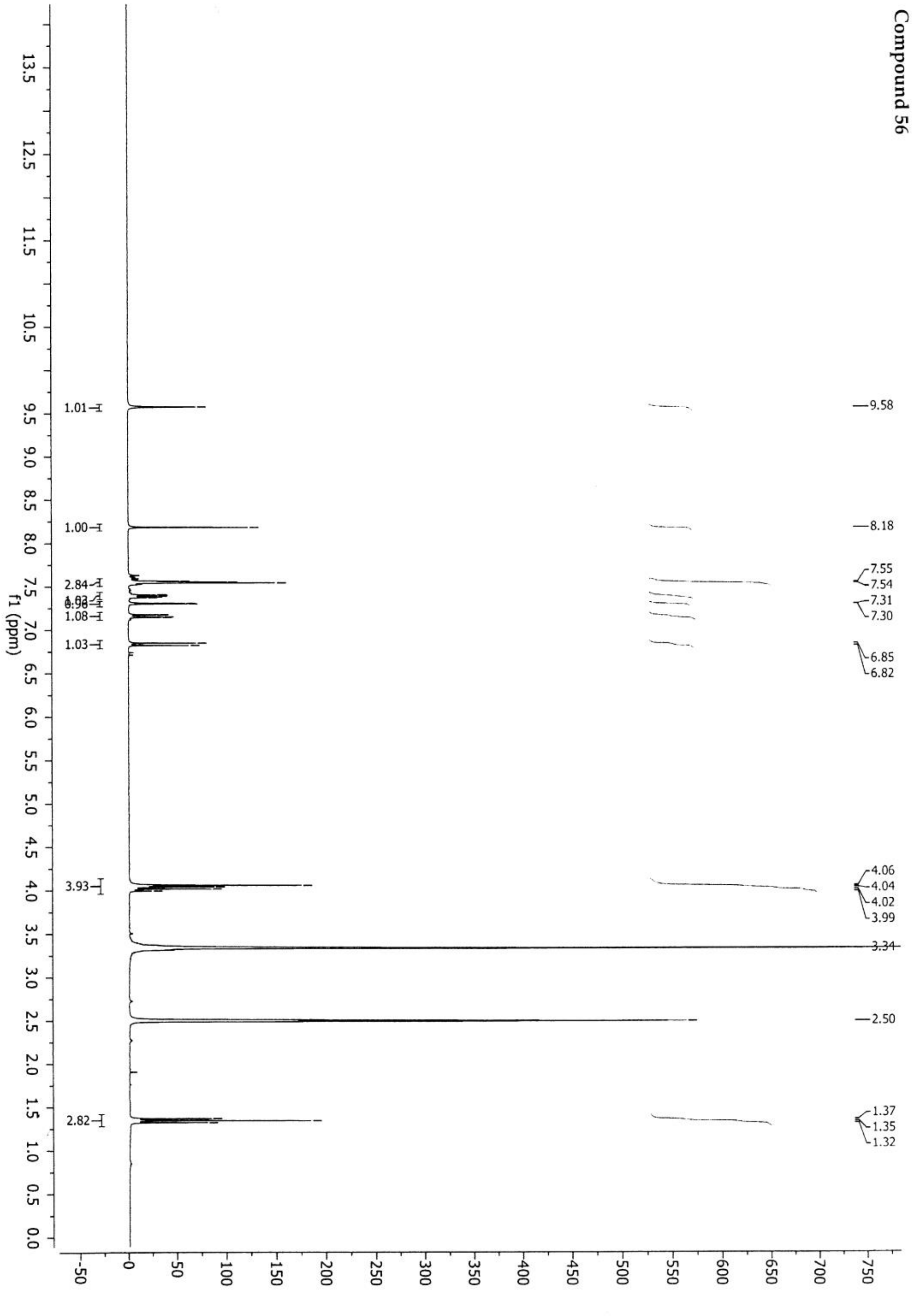

Fig. S25. ^{1}H NMR spectrum for compound **56**

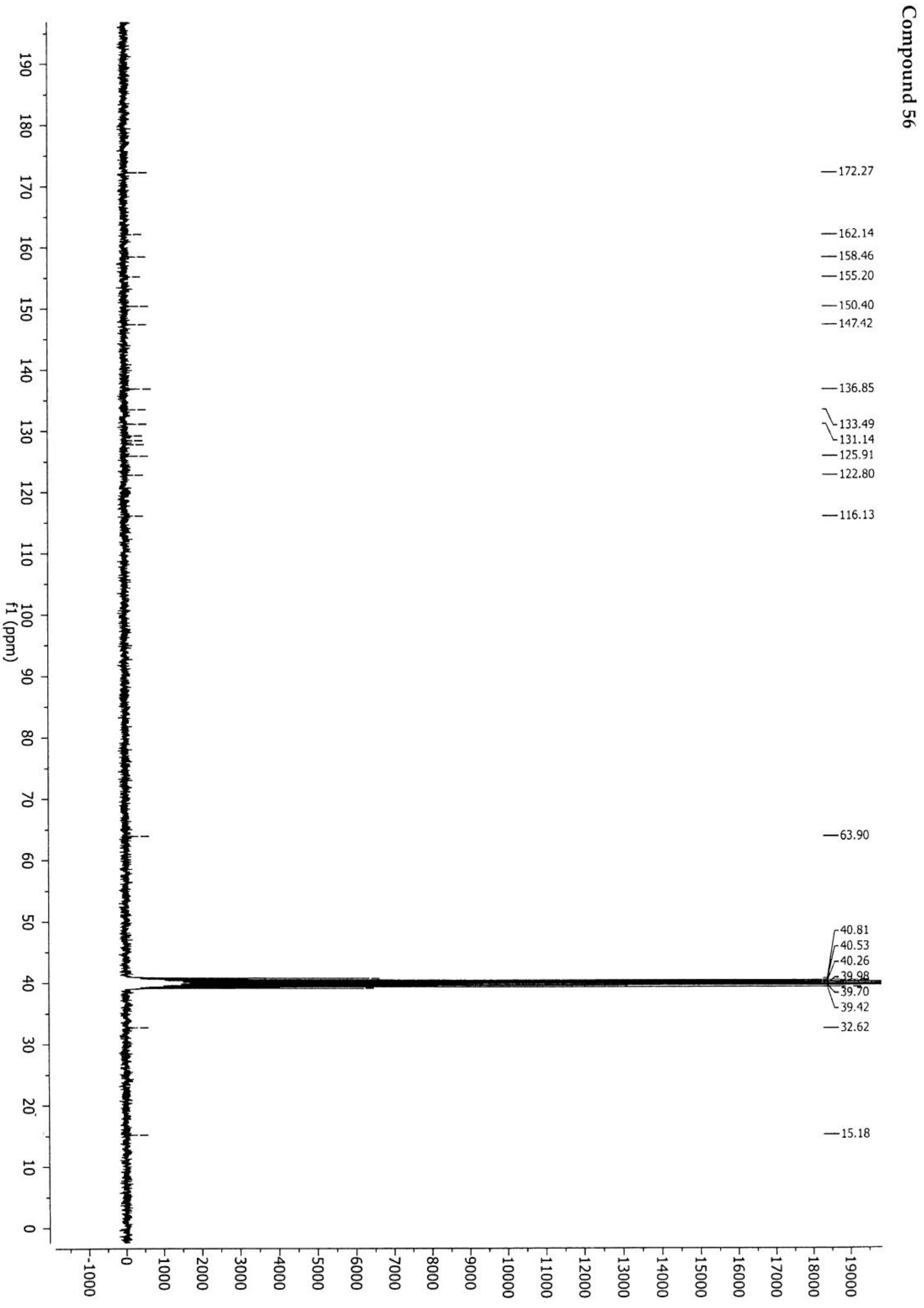

Fig. S26. ^{13}C NMR spectrum for compound **56**

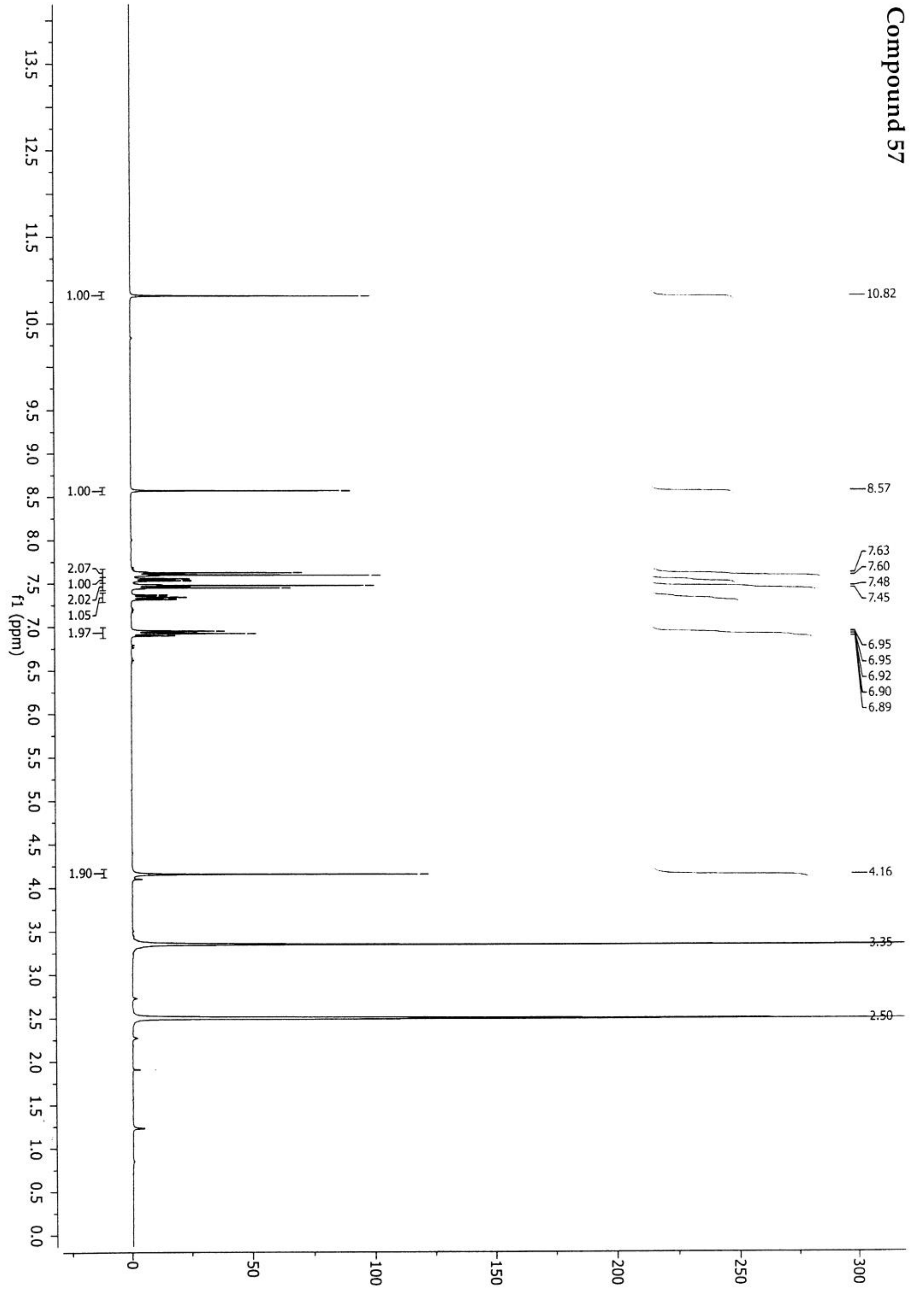

Fig. S27. ^{1}H NMR spectrum for compound **57**

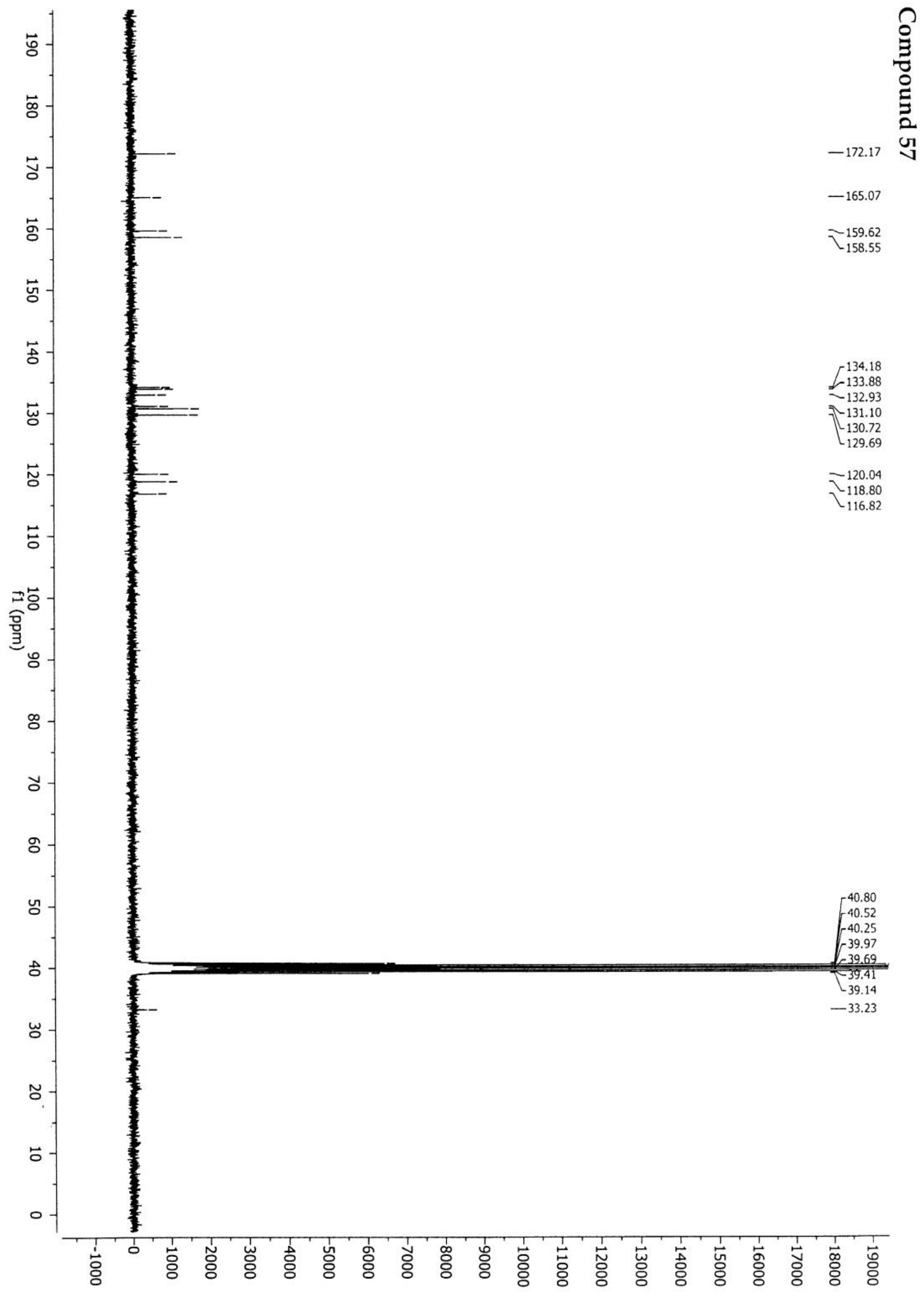

Fig. S28. ^{13}C NMR spectrum for compound **57**

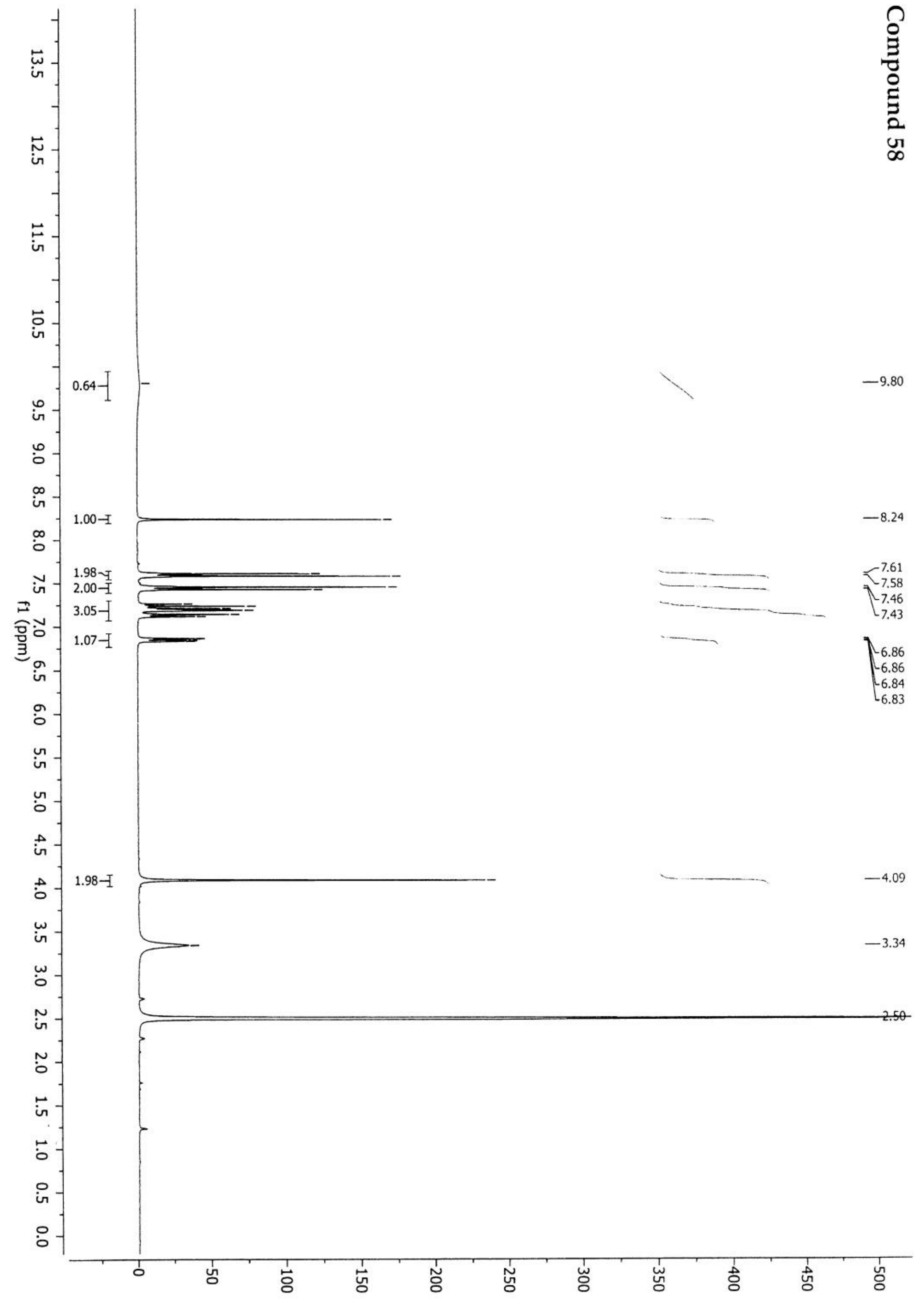

Fig. S29. ¹H NMR spectrum for compound **58**

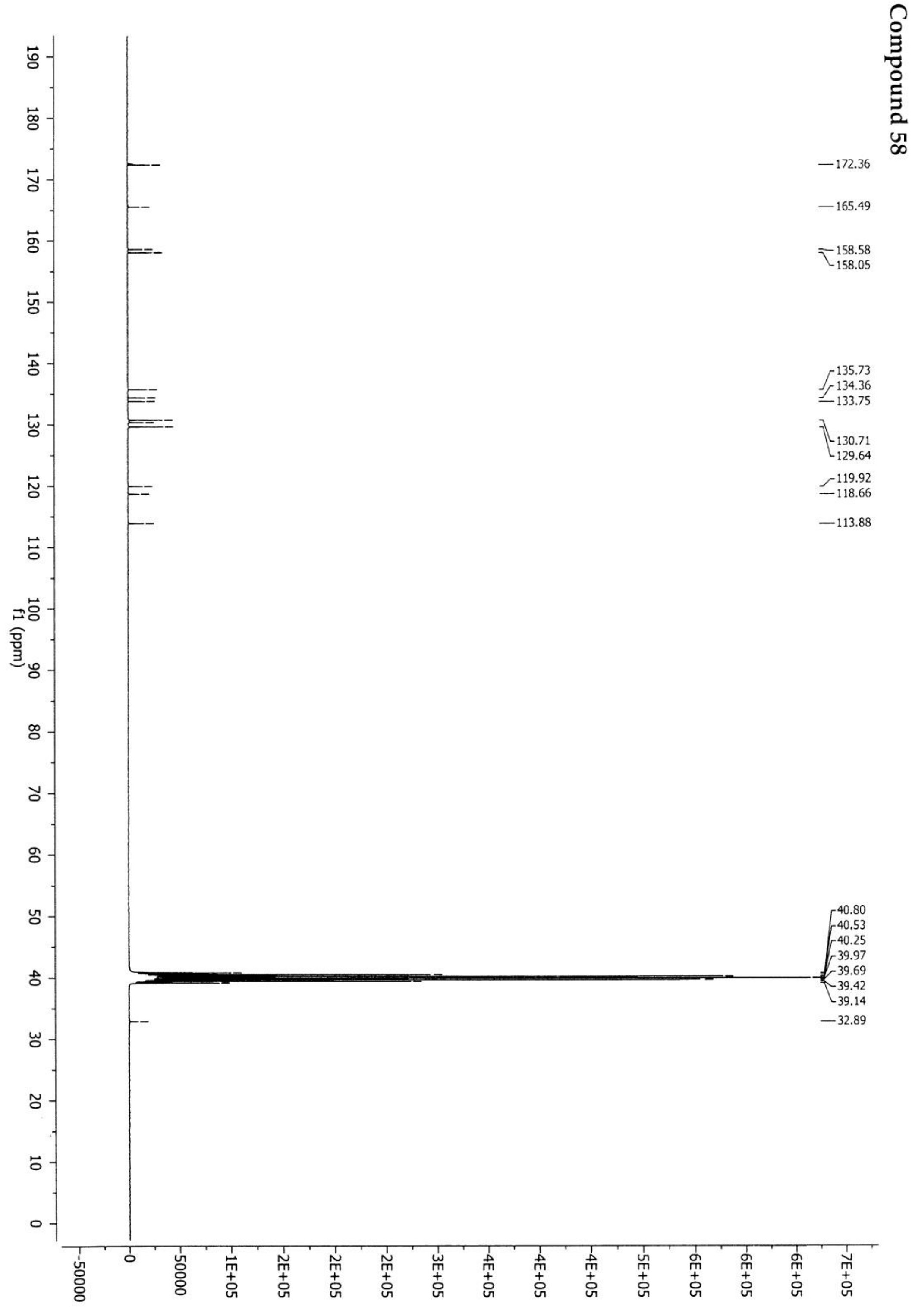

Fig. S30. ^{13}C NMR spectrum for compound **58**

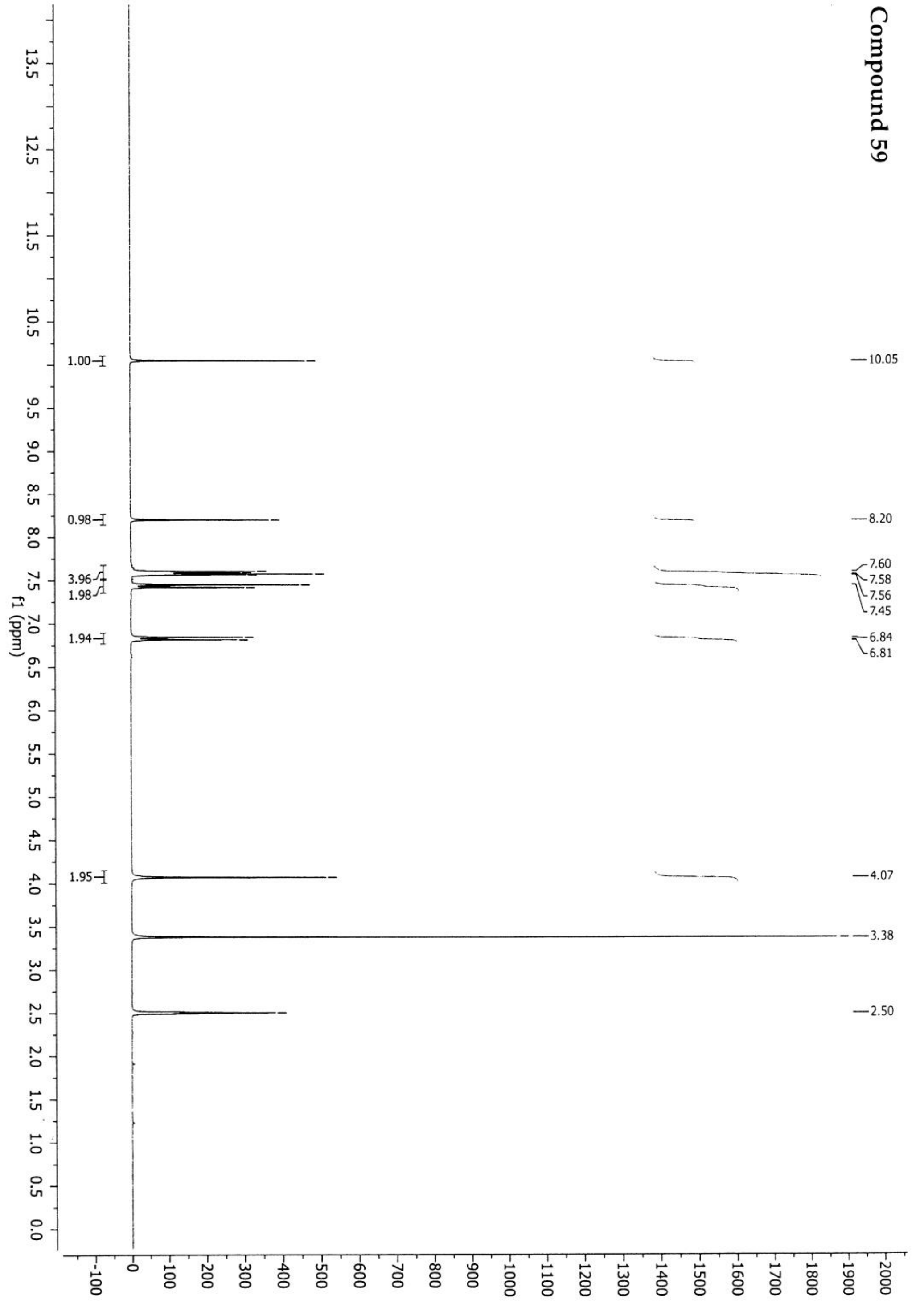

Fig. S31. ^{1}H NMR spectrum for compound **59**

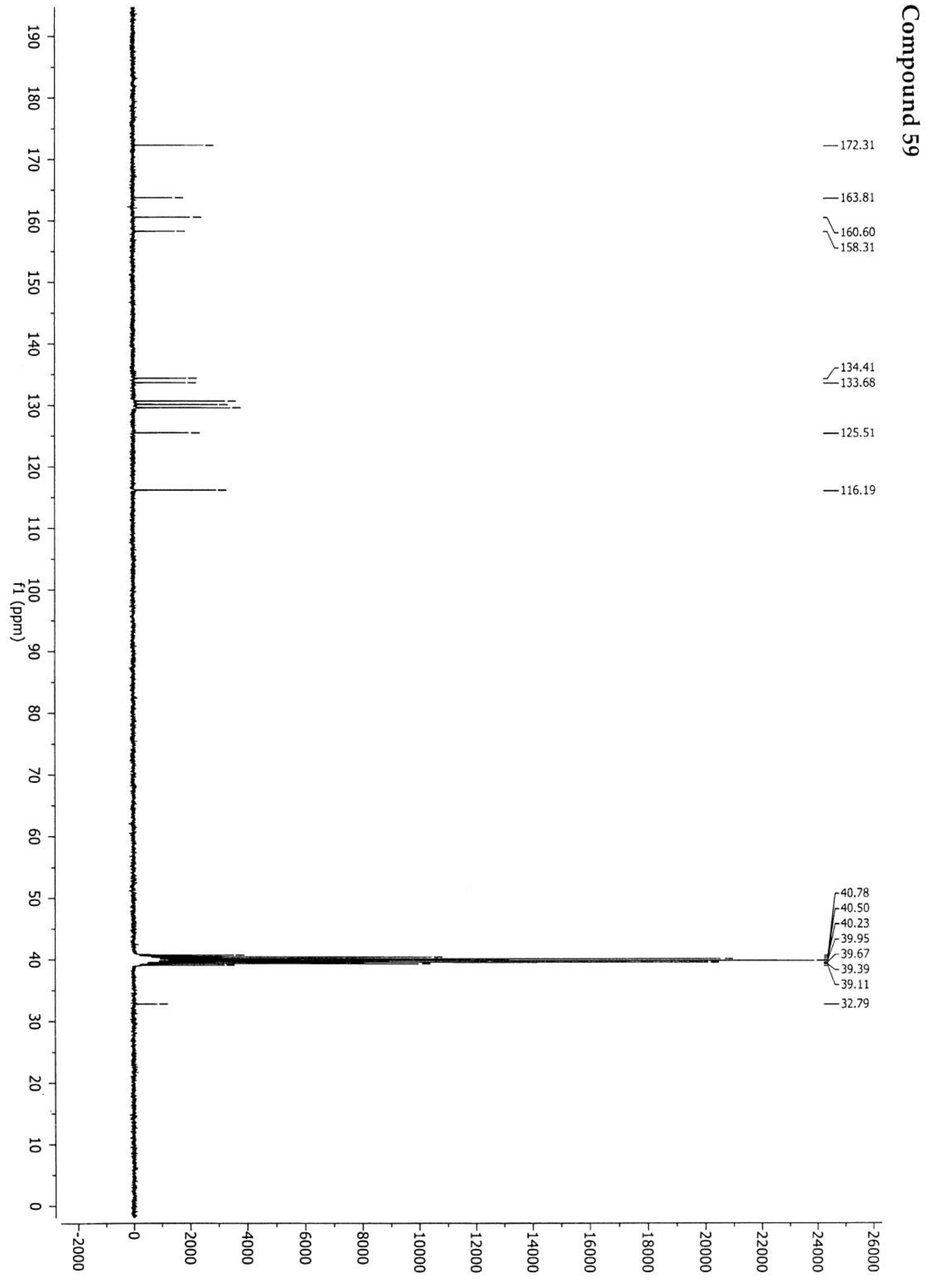

Fig. S32. ^{13}C NMR spectrum for compound **59**

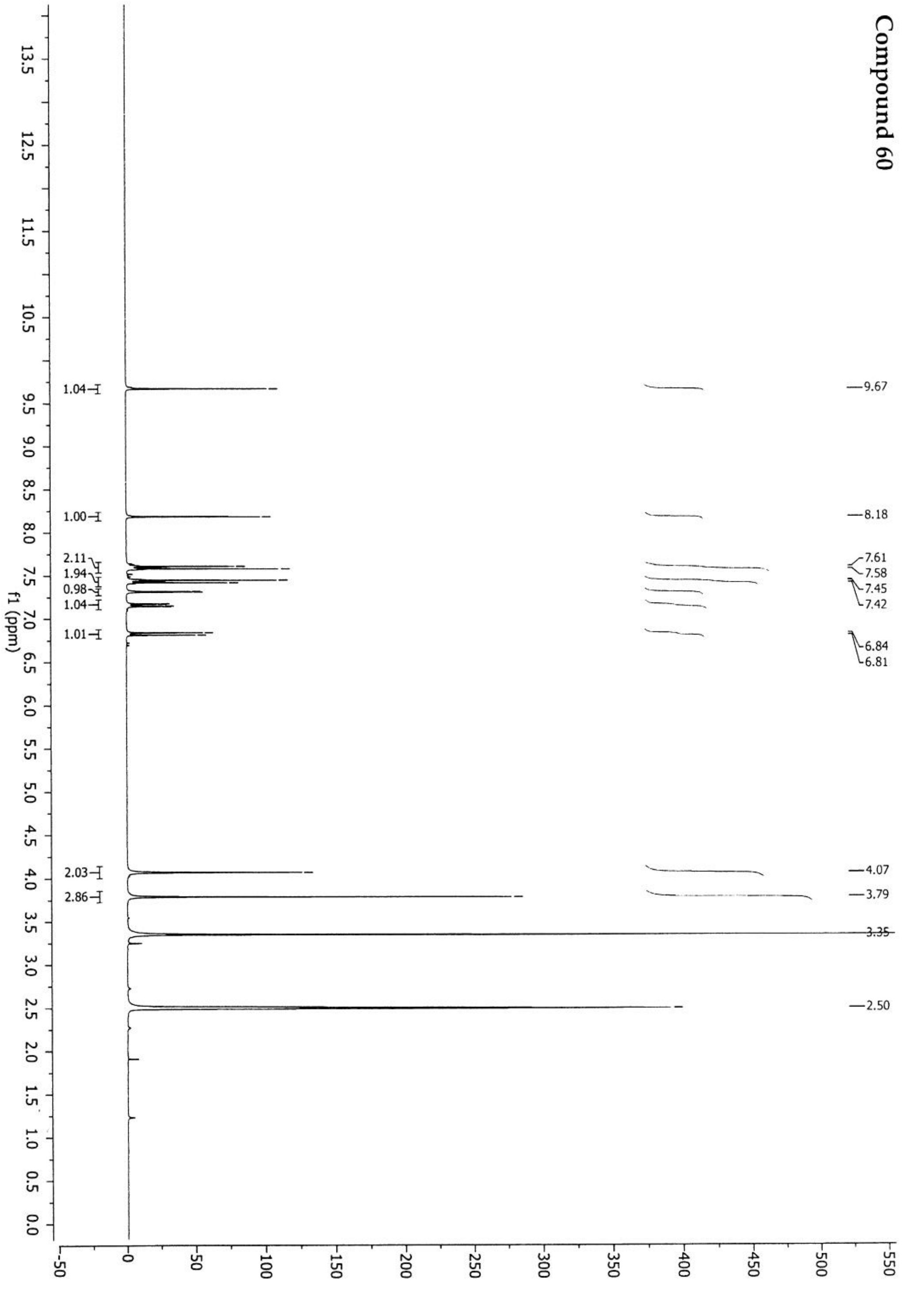

Fig. S33. ^{1}H NMR spectrum for compound **60**

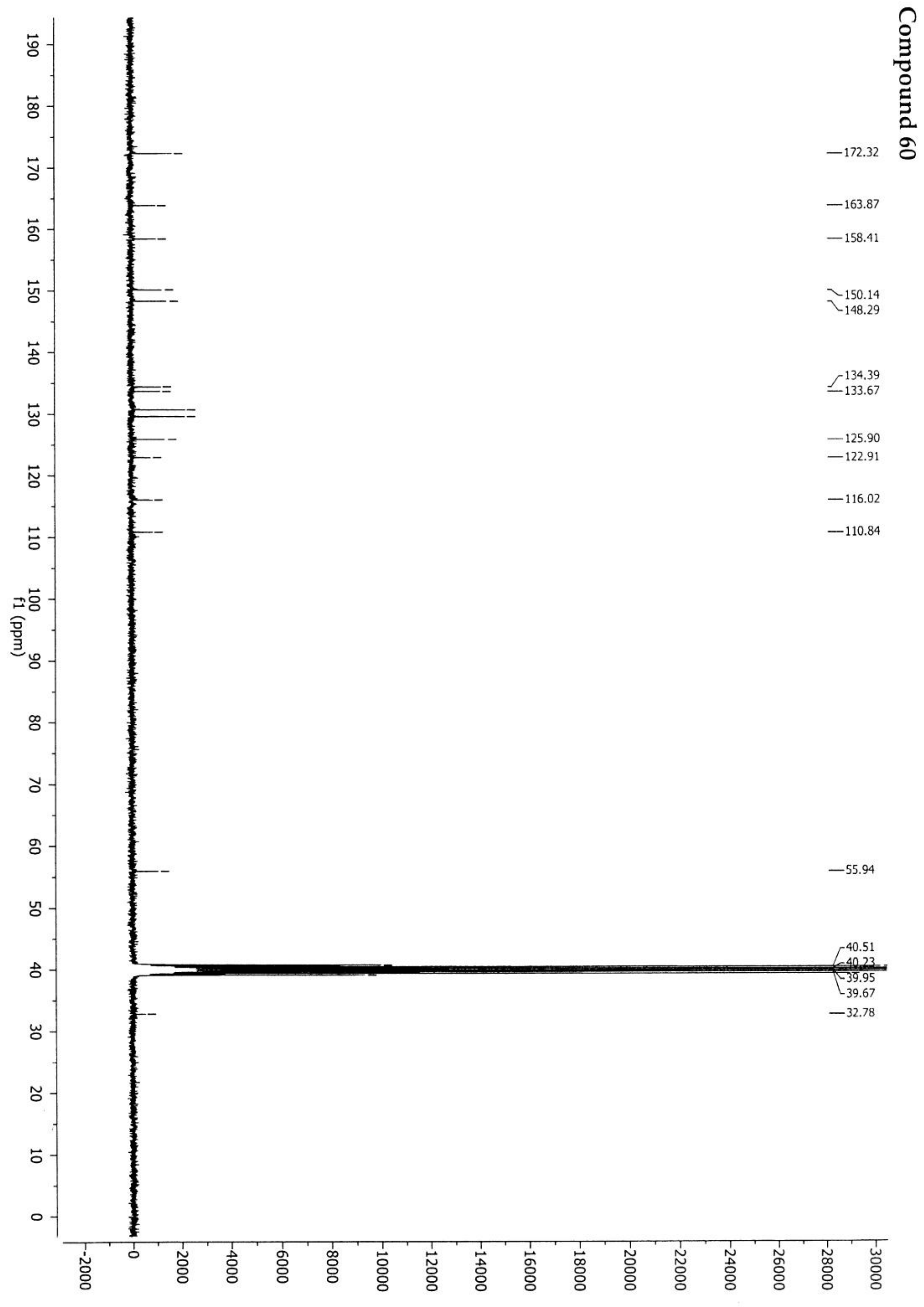

Fig. S34. ^{13}C NMR spectrum for compound **60**

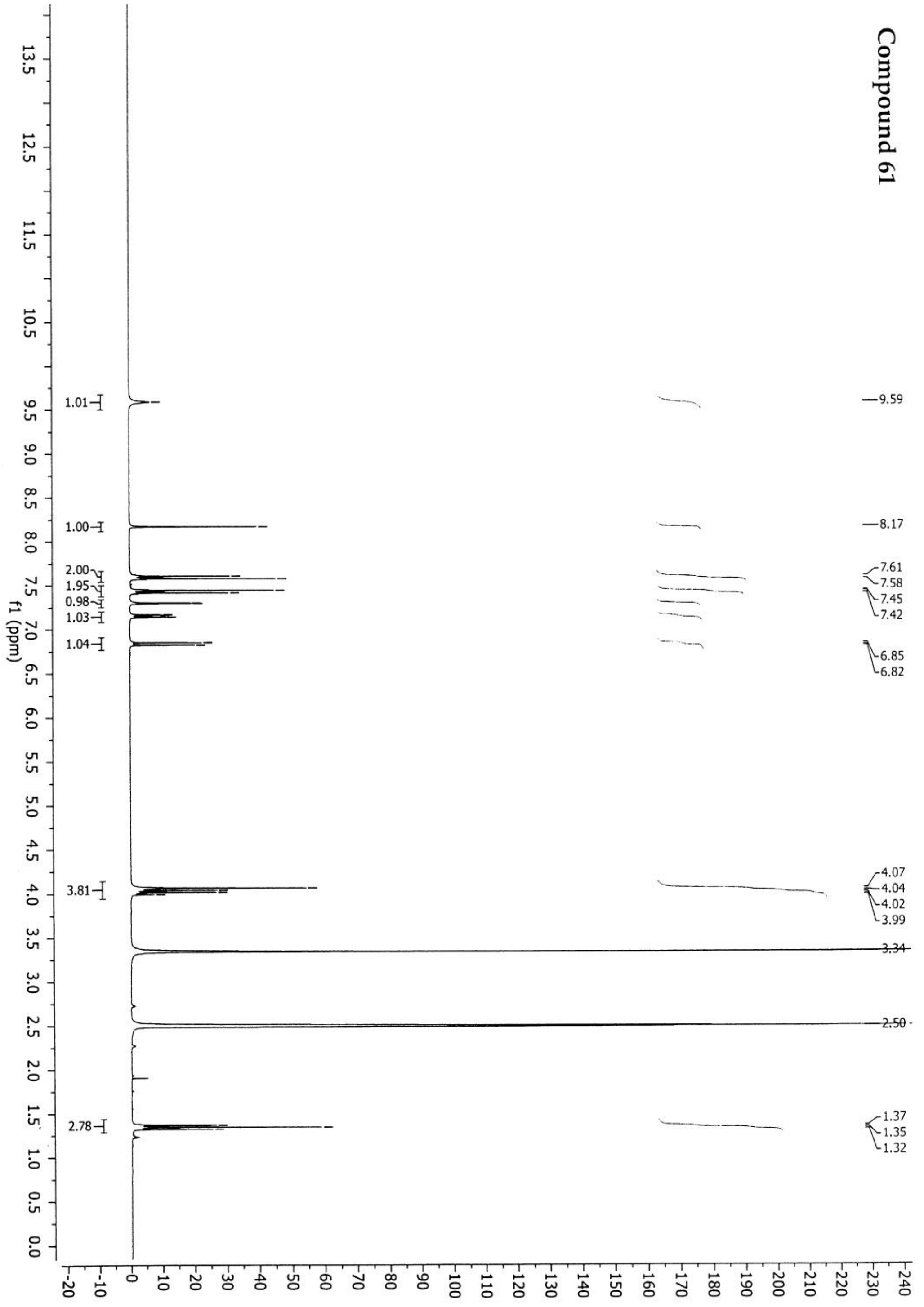

Fig. S35. ^{1}H NMR spectrum for compound **61**

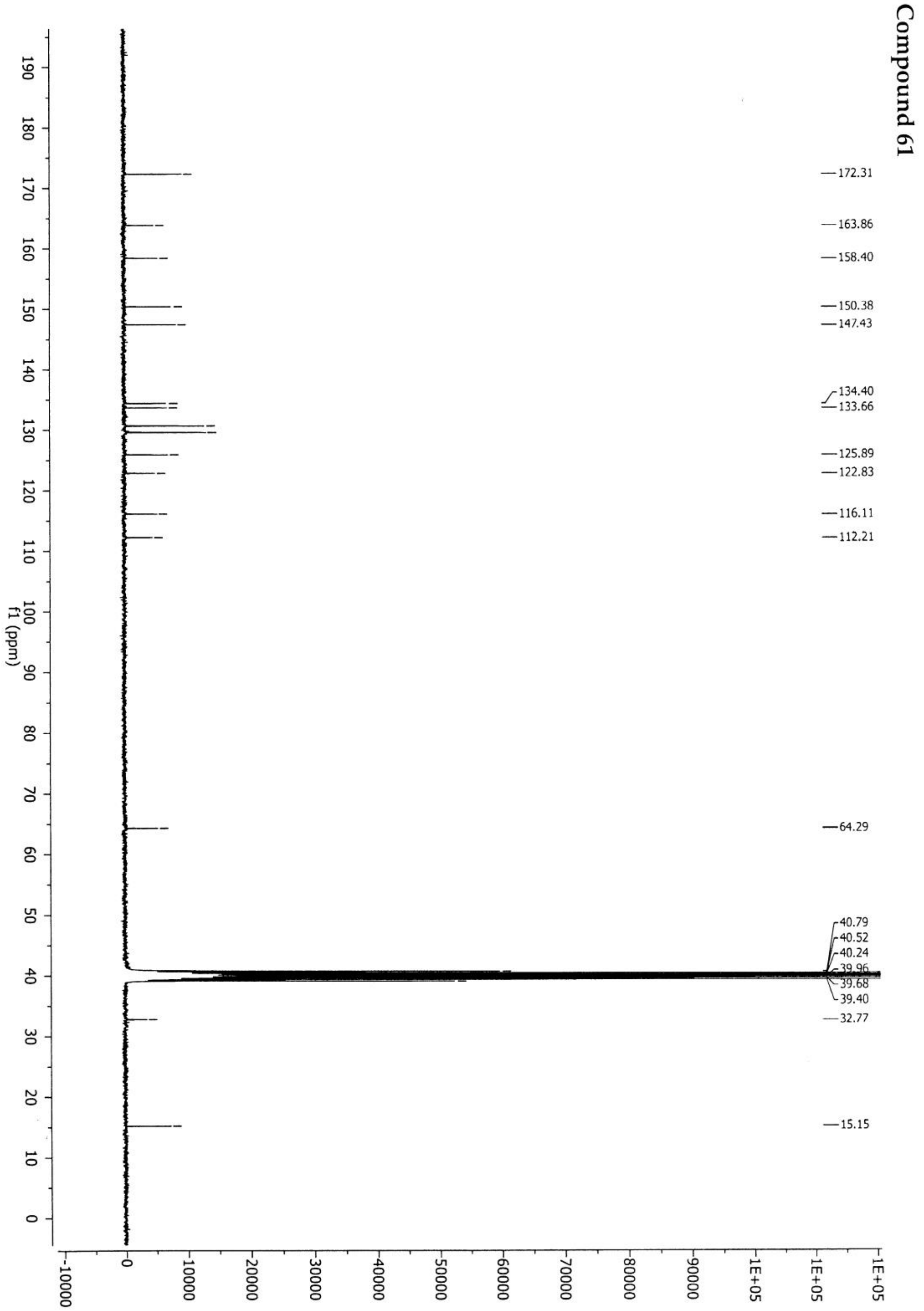

Fig. S36. ^{13}C NMR spectrum for compound **61**

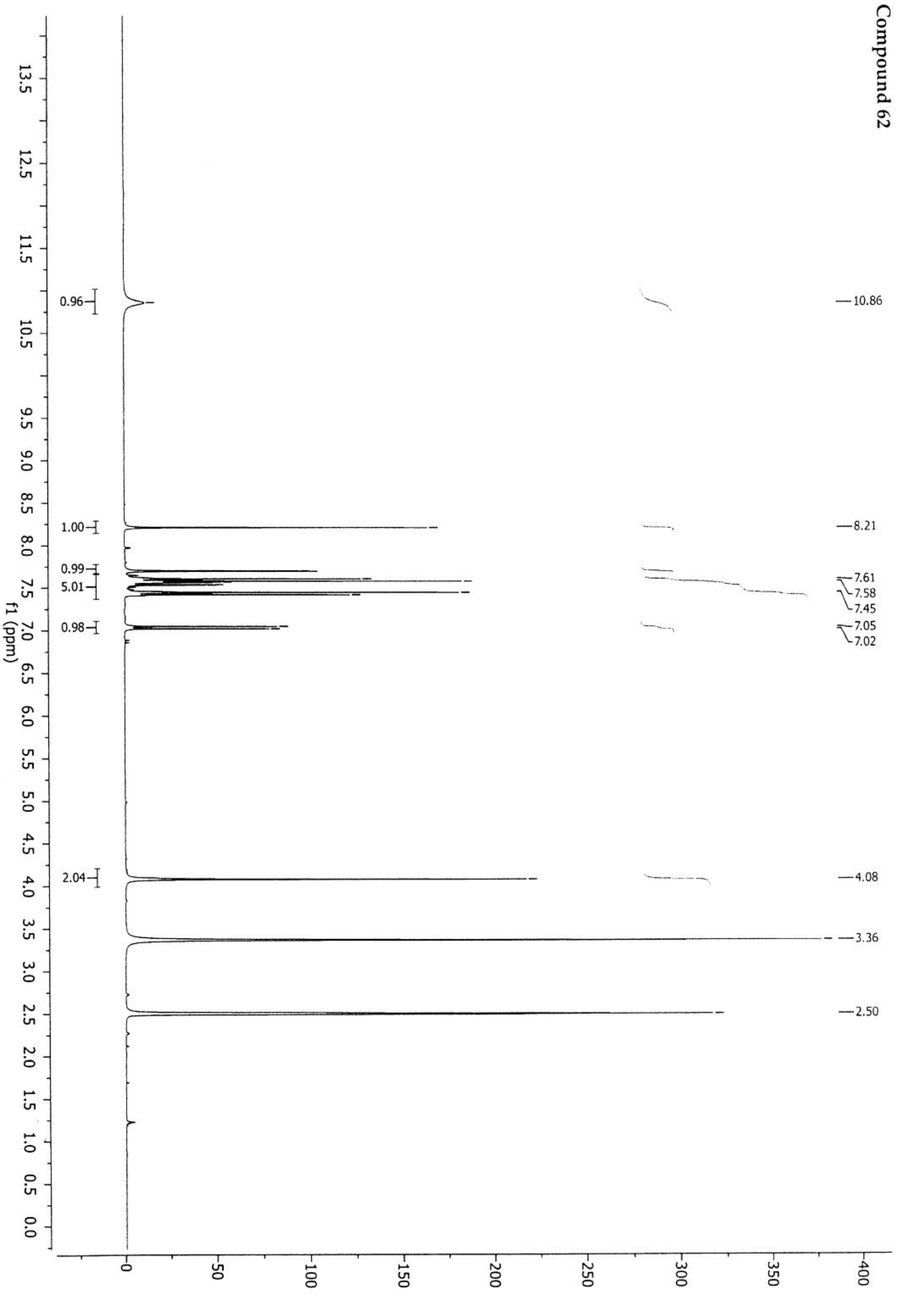

Fig. S37. ¹H NMR spectrum for compound **62**

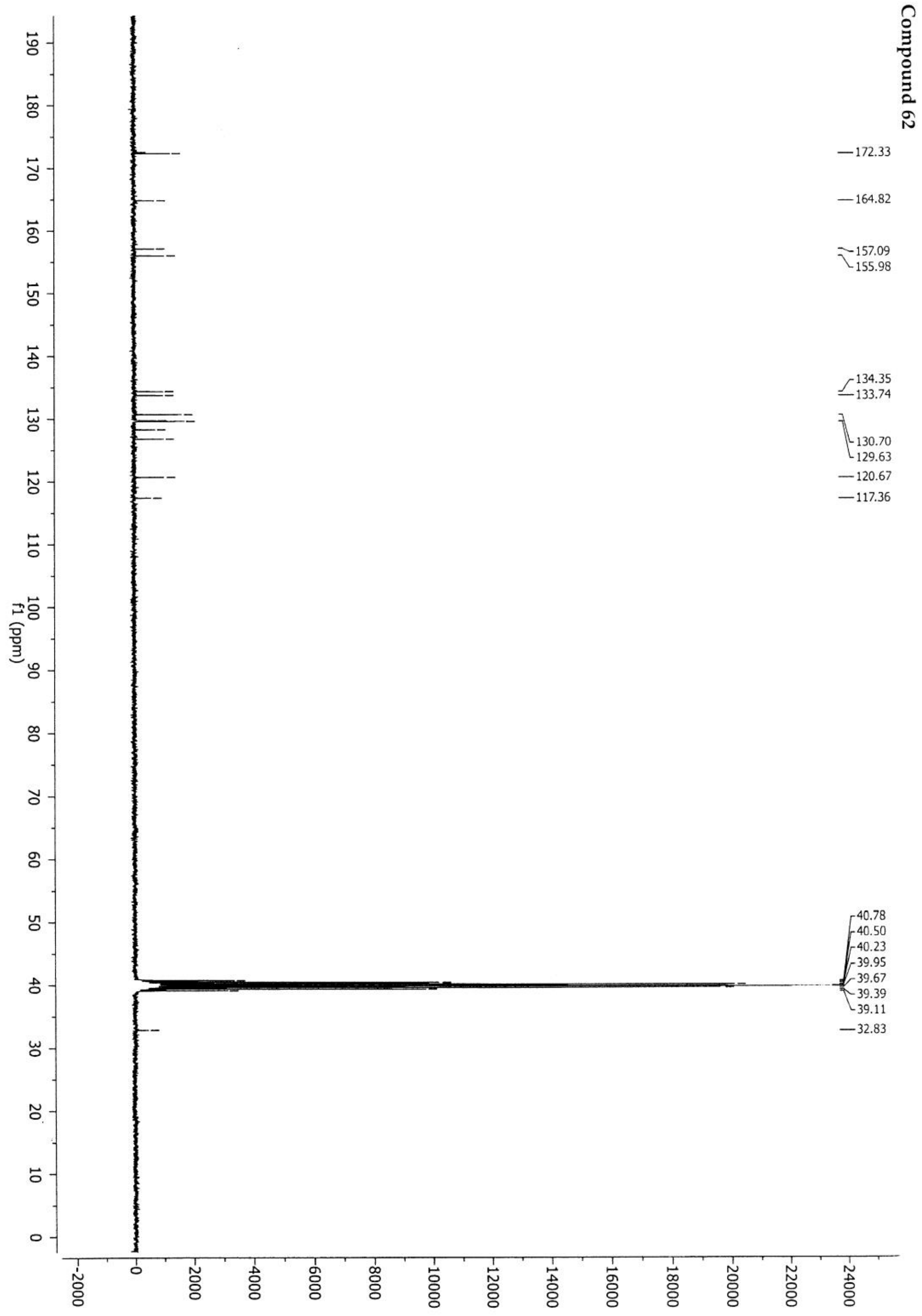

Fig. S38. ^{13}C NMR spectrum for compound **62**

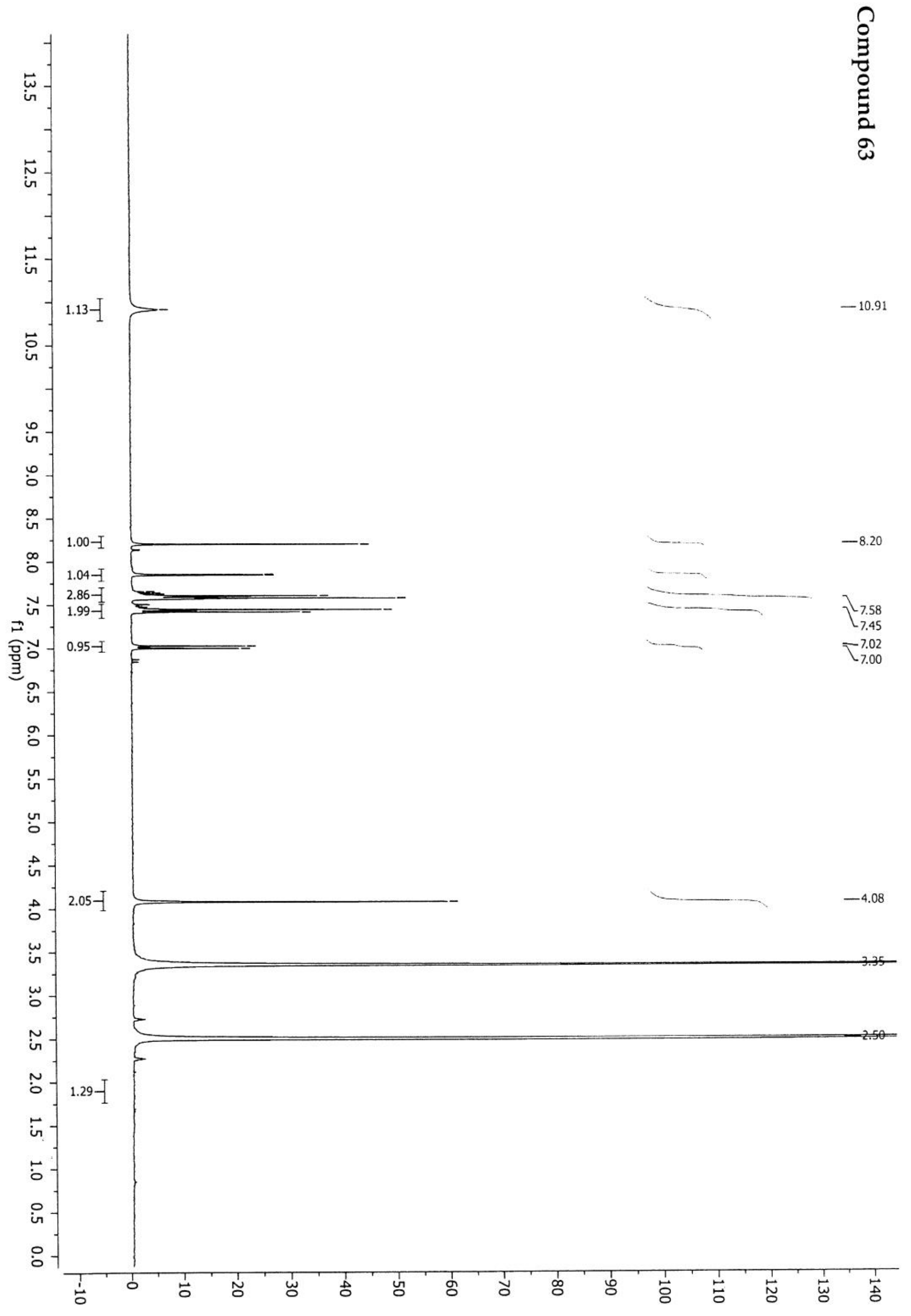

Fig. S39. ¹H NMR spectrum for compound **63**

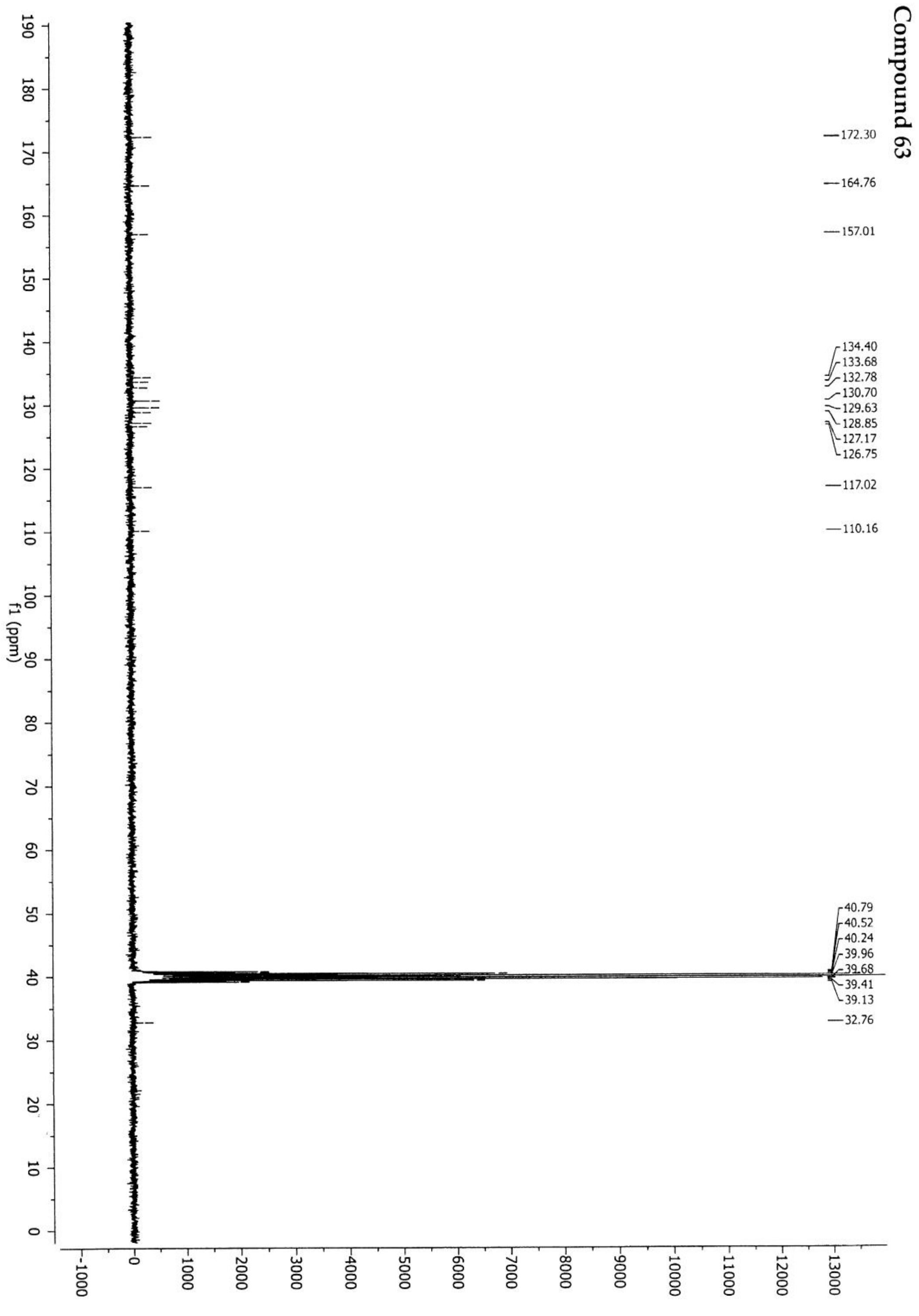

Fig. S40. ^{13}C NMR spectrum for compound **63**

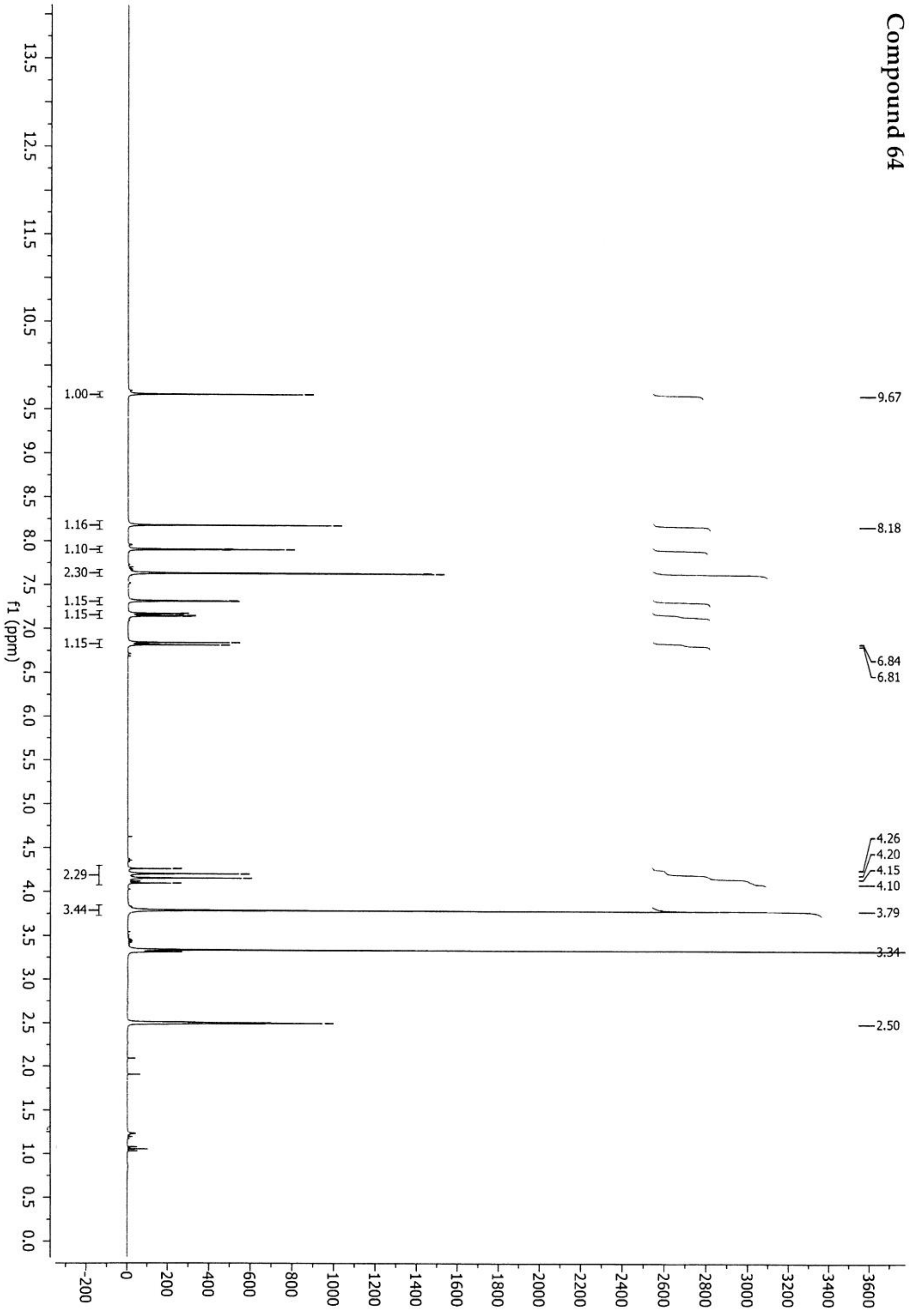

Fig. S41. ^{1}H NMR spectrum for compound **64**

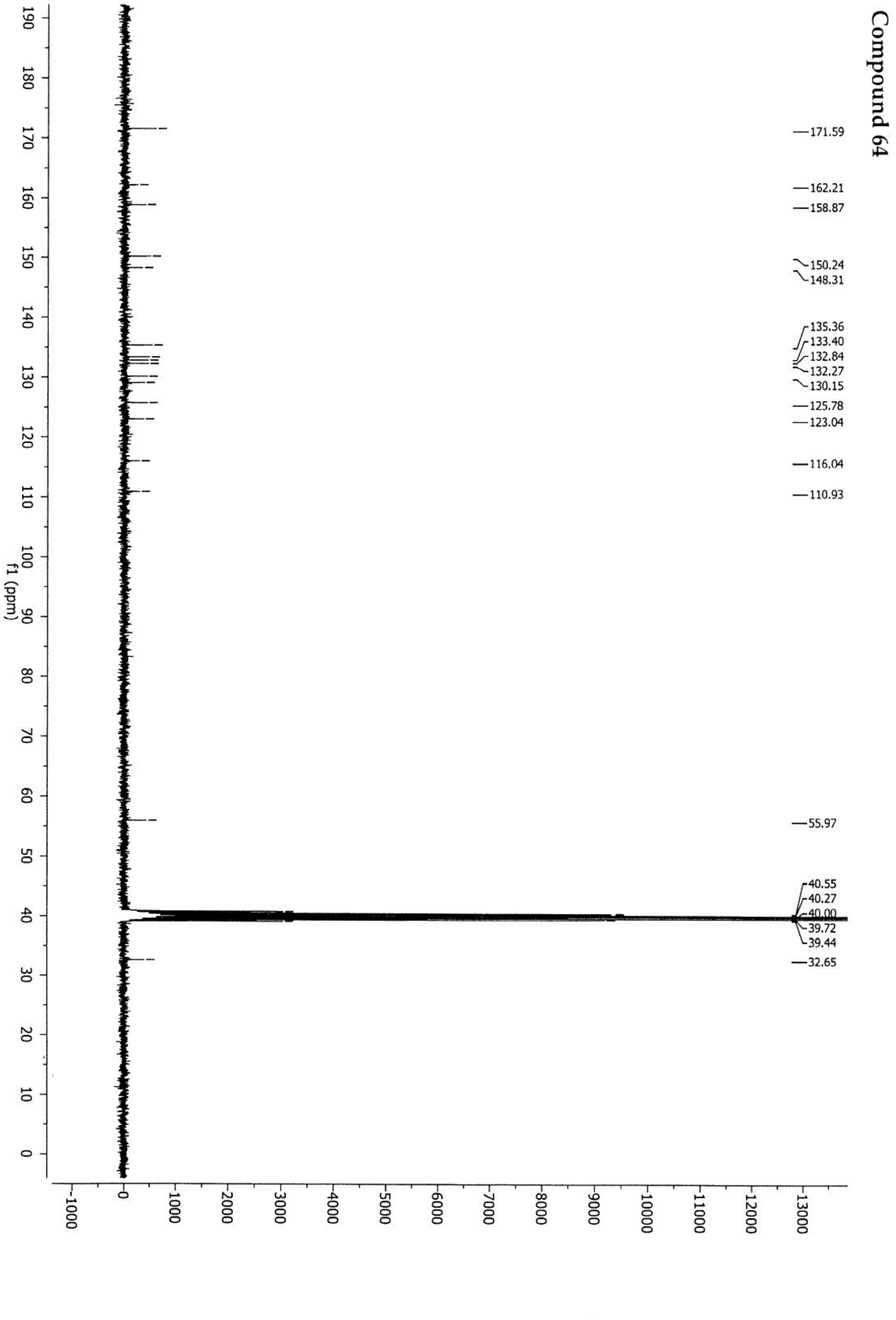

Fig. S42. ^{13}C NMR spectrum for compound **64**

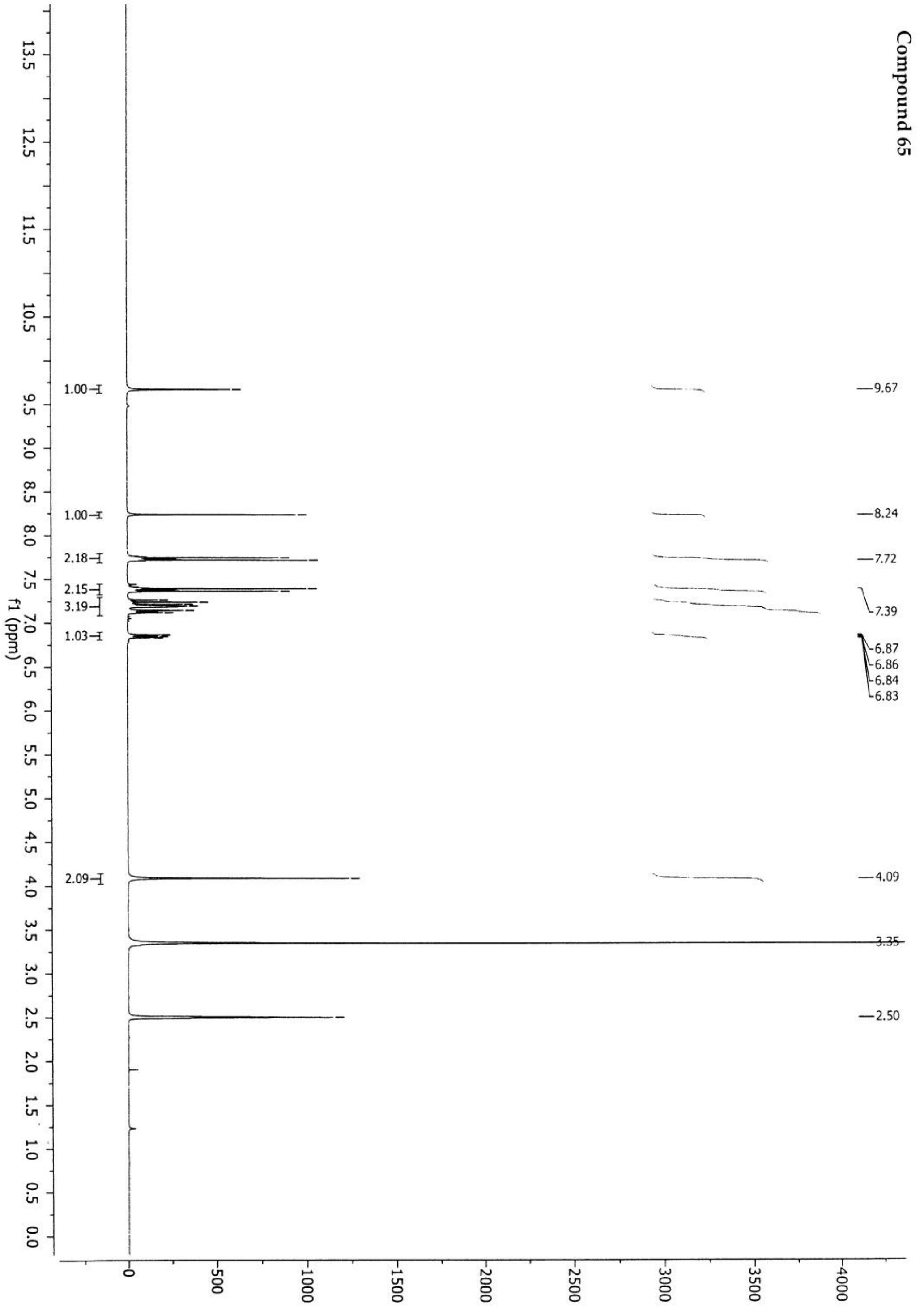

Fig. S43. ¹H NMR spectrum for compound **65**

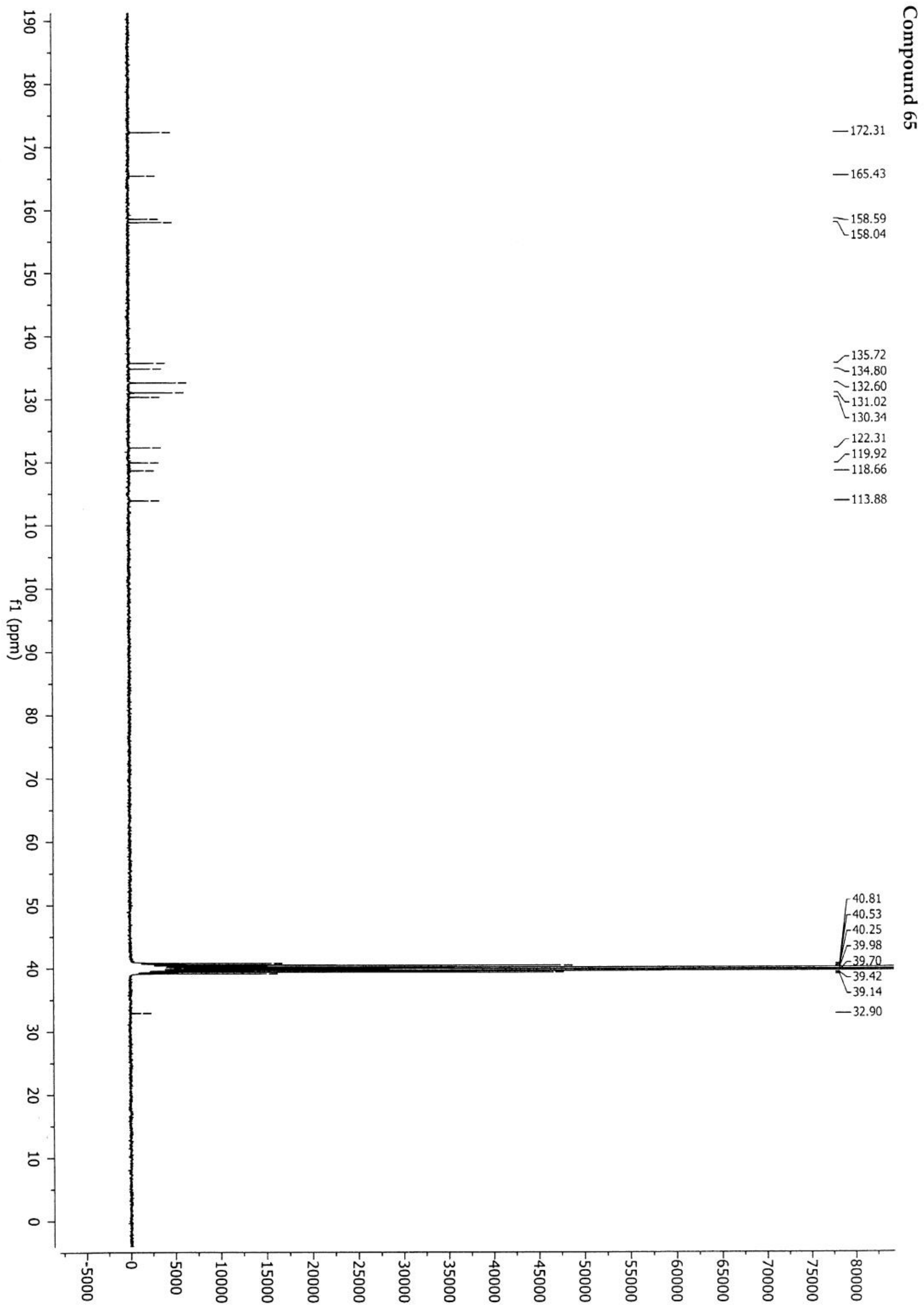

Fig. S44. ^{13}C NMR spectrum for compound **65**

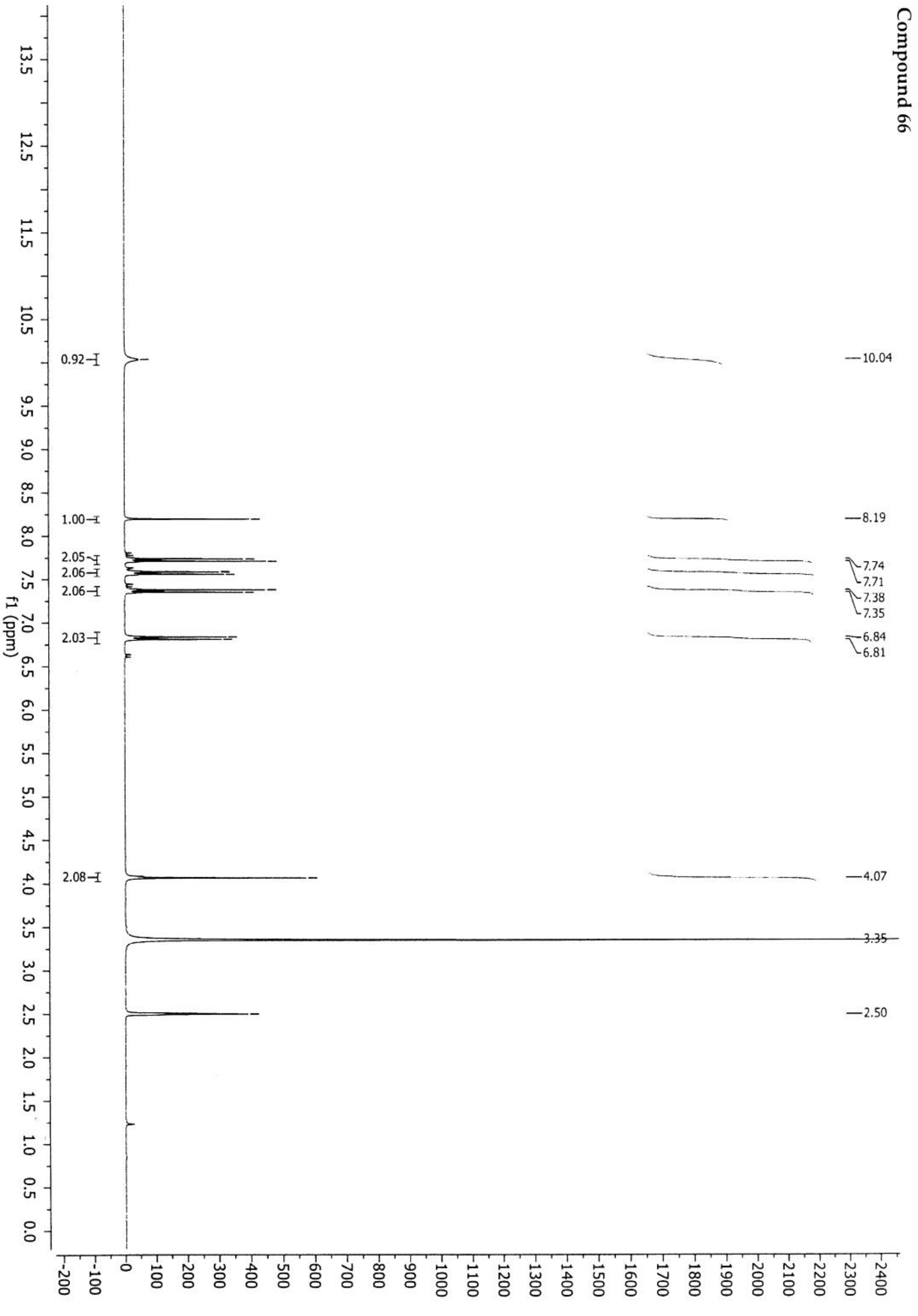

Fig. S45. ^{1}H NMR spectrum for compound **66**

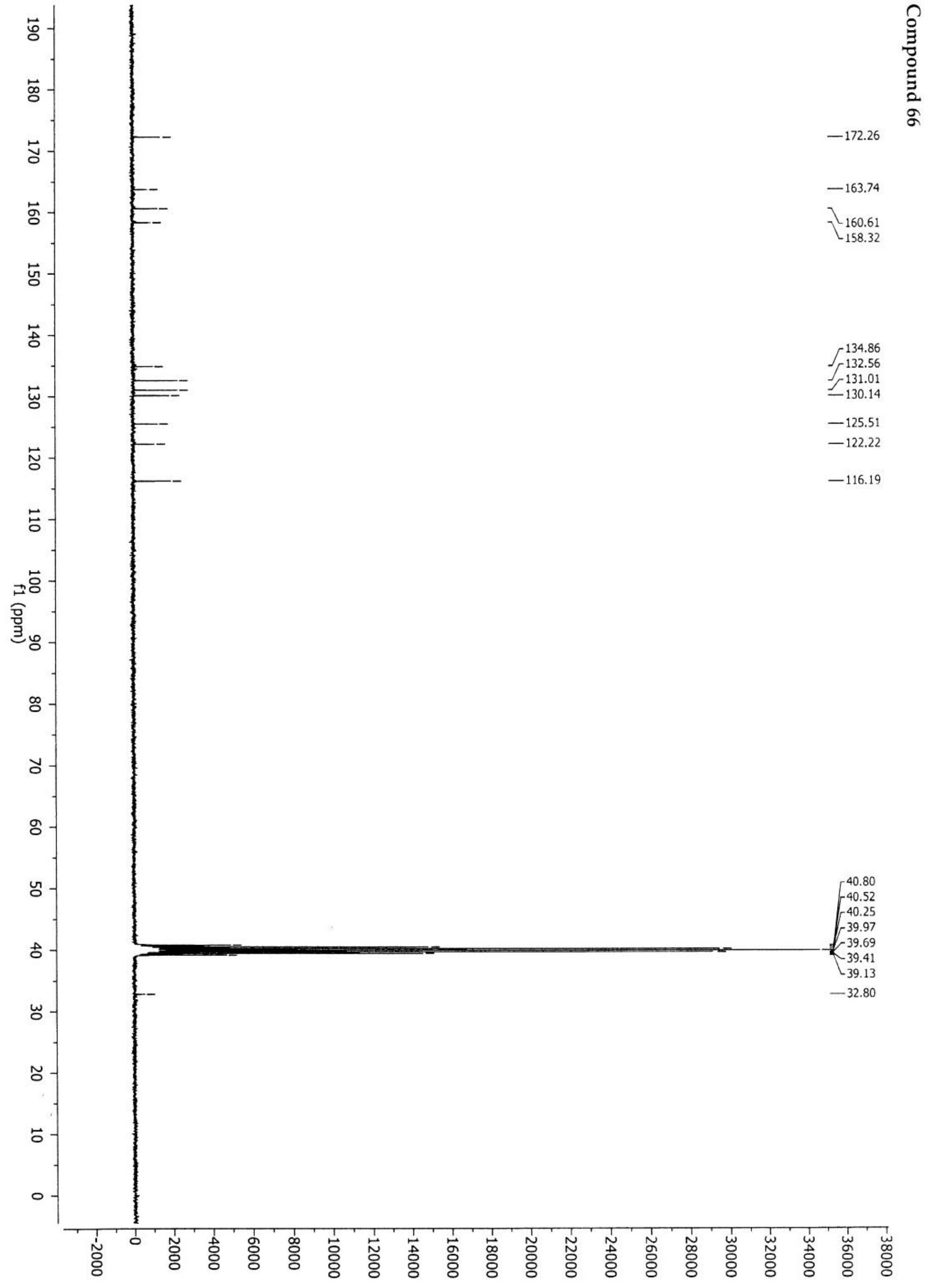

Fig. S46. ^{13}C NMR spectrum for compound **66**

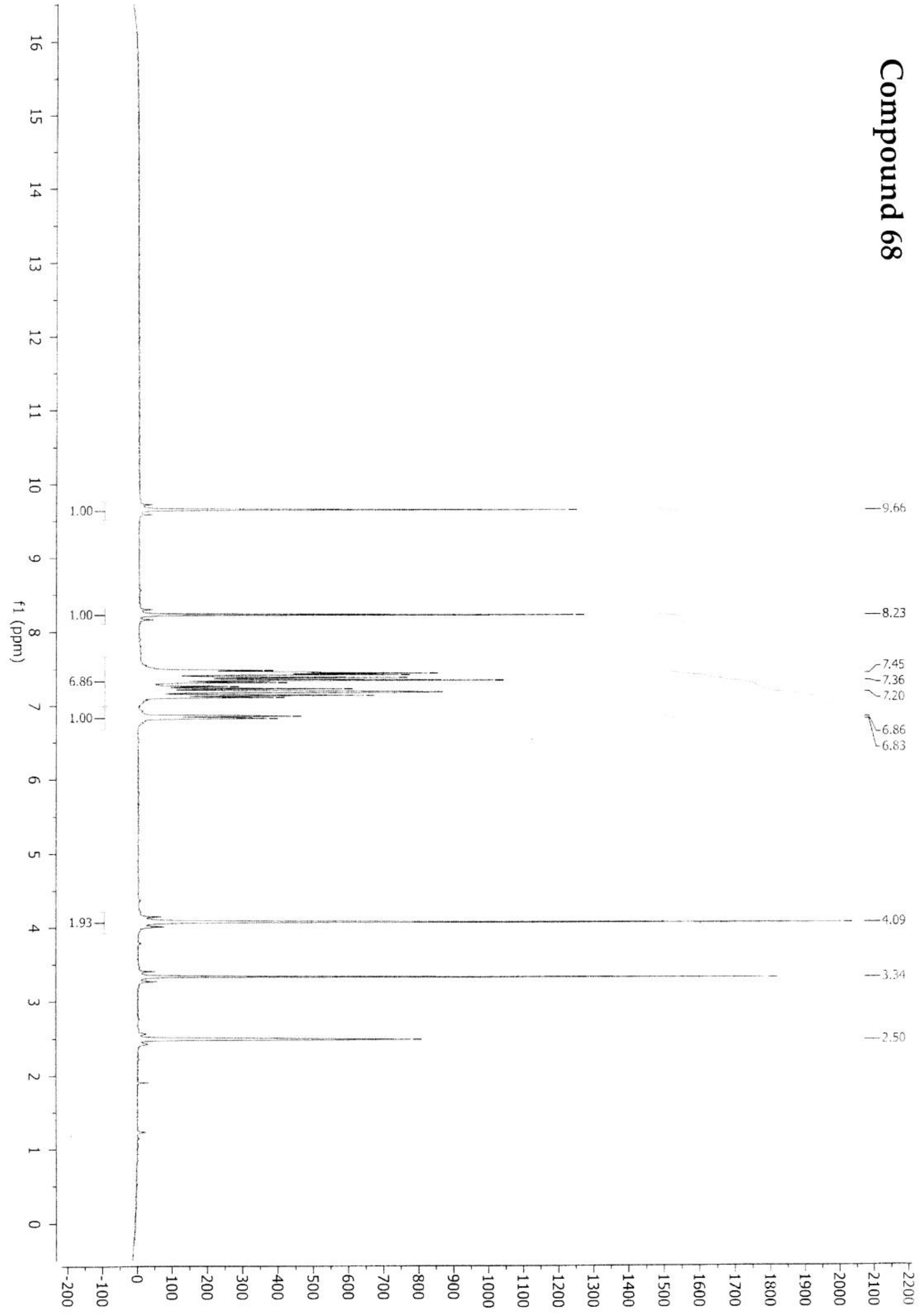

Fig. S47. ^{1}H NMR spectrum for compound **68**

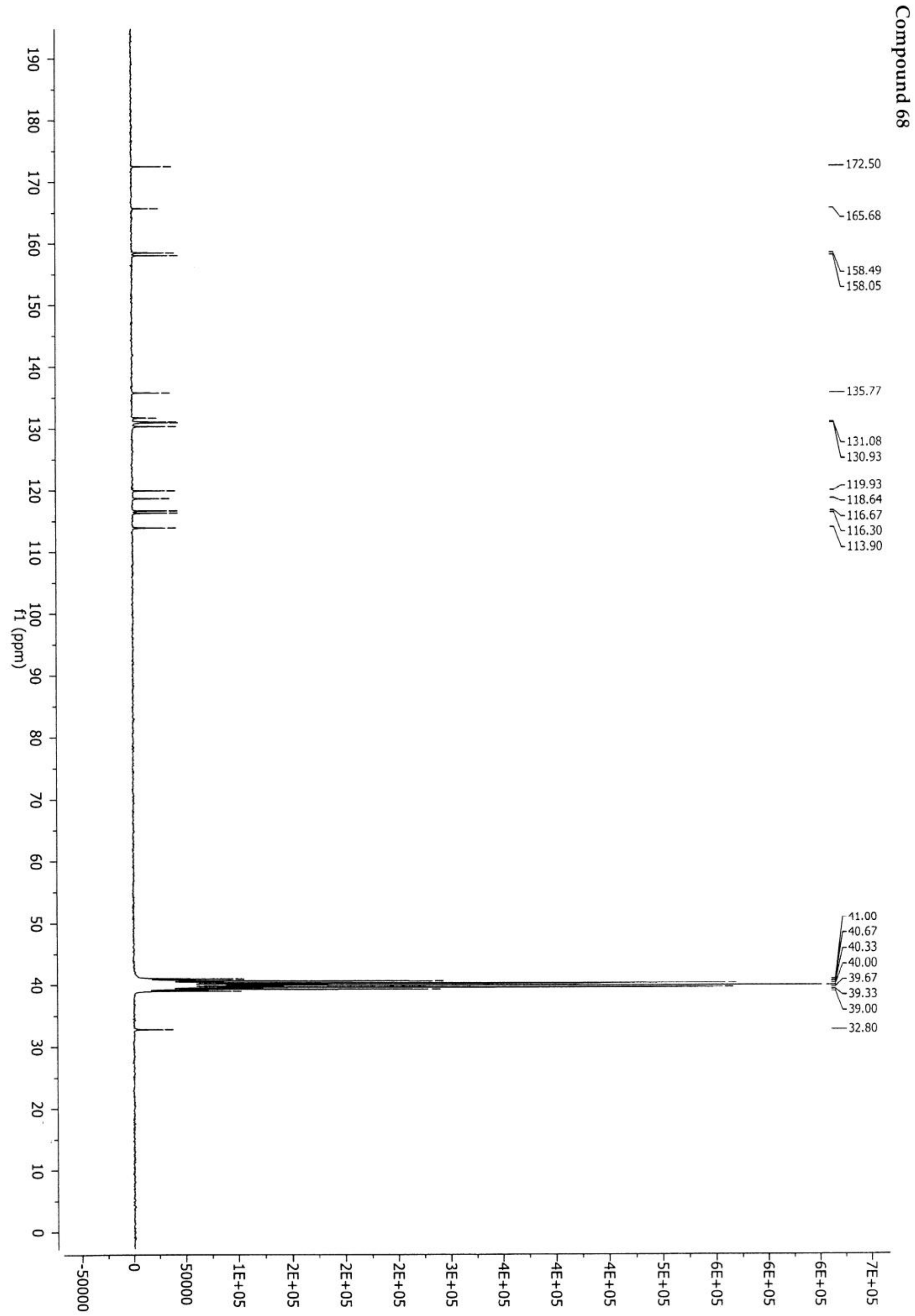

Fig. S48. ^{13}C NMR spectrum for compound **68**

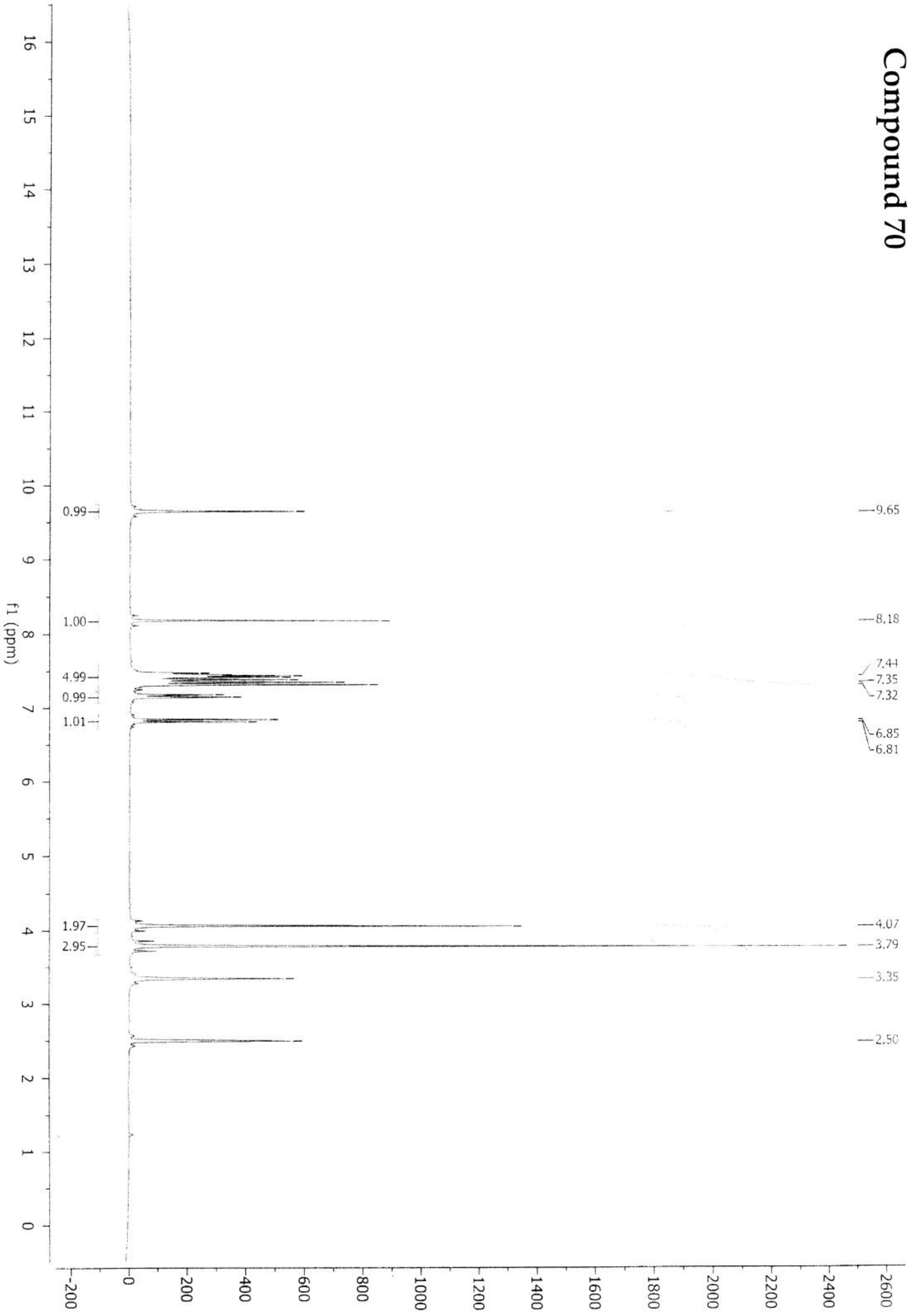

Fig. S49. ^{1}H NMR spectrum for compound **70**

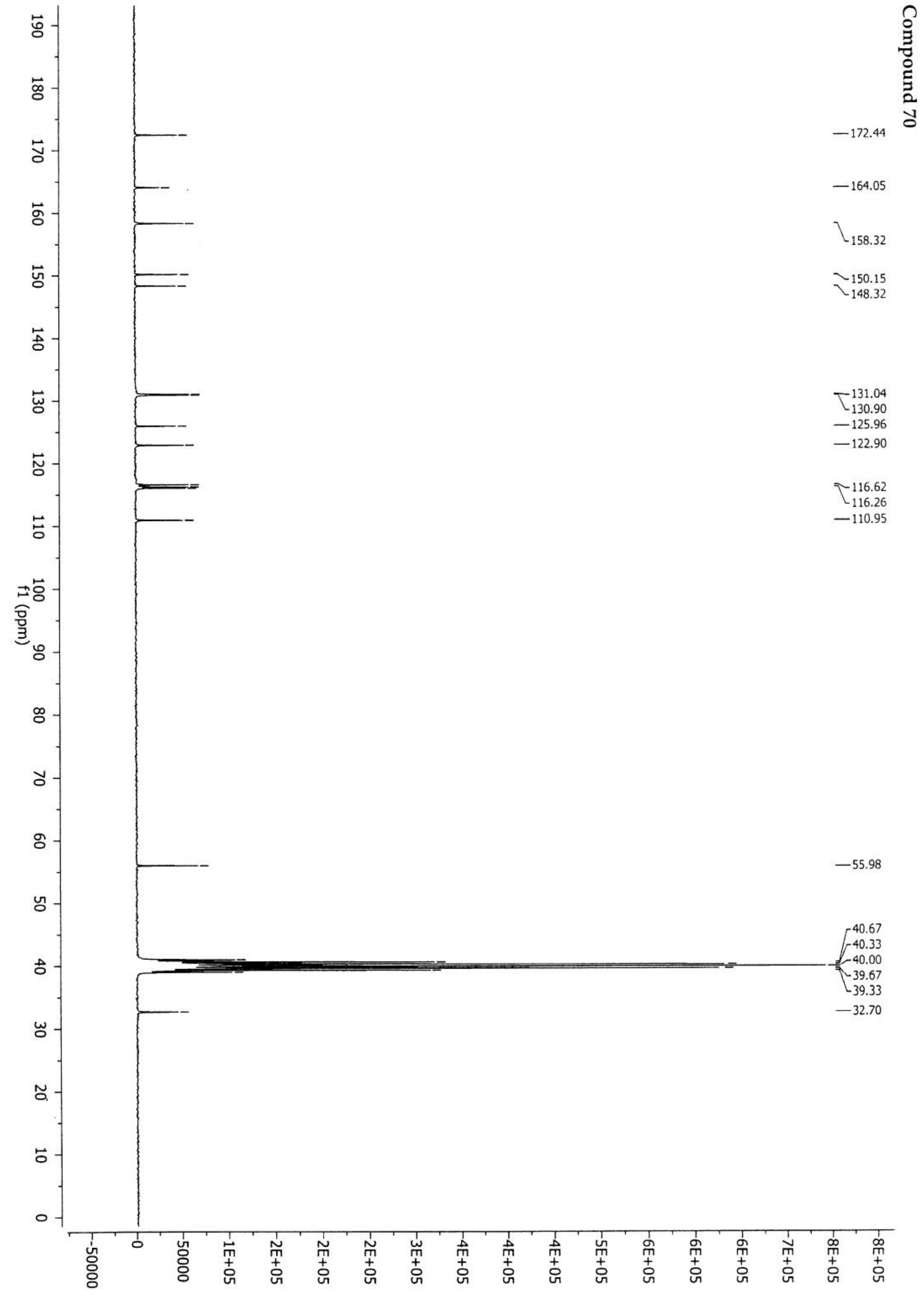

Fig. S50. ^{13}C NMR spectrum for compound **70**

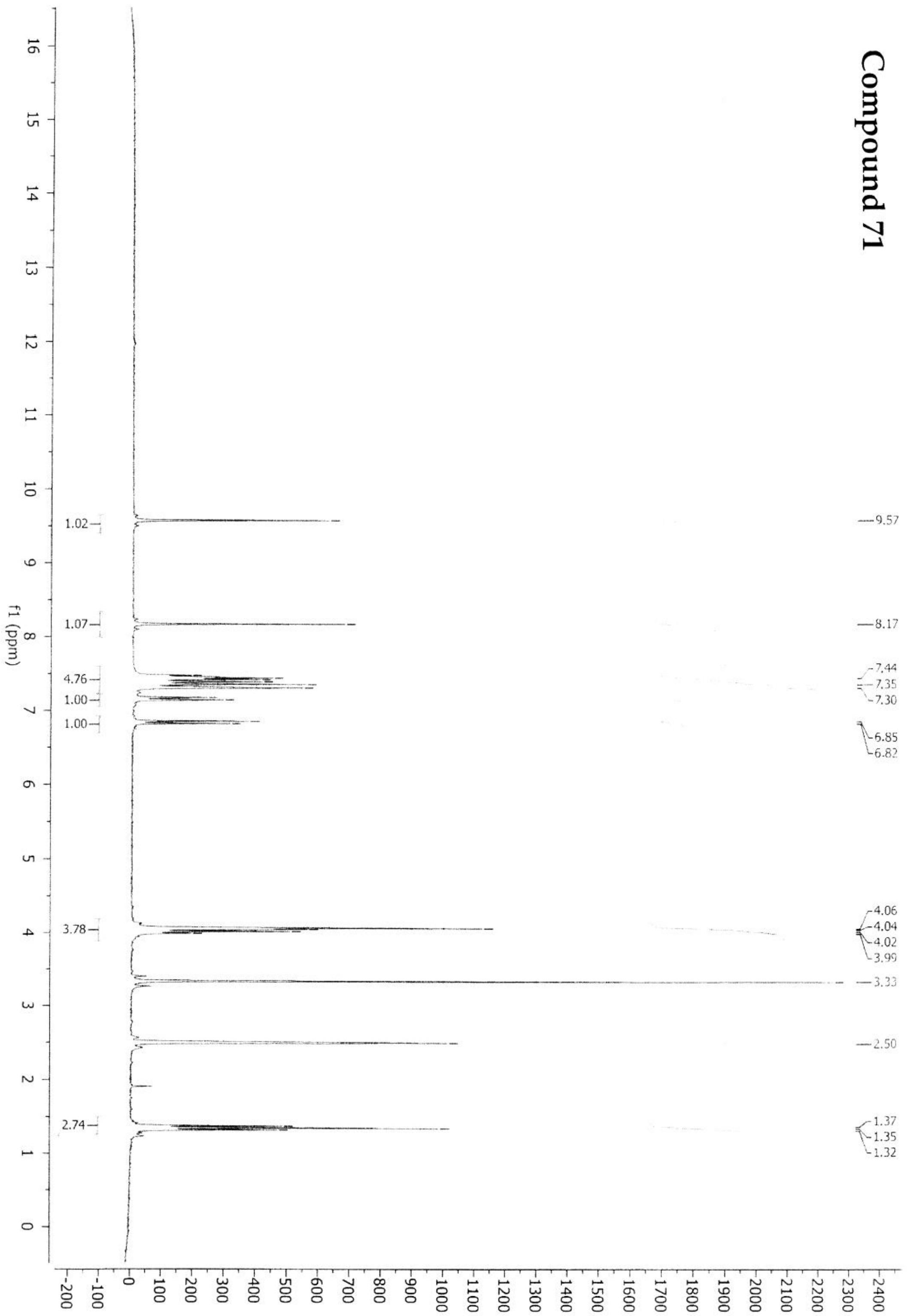

Fig. S51. ¹H NMR spectrum for compound **71**

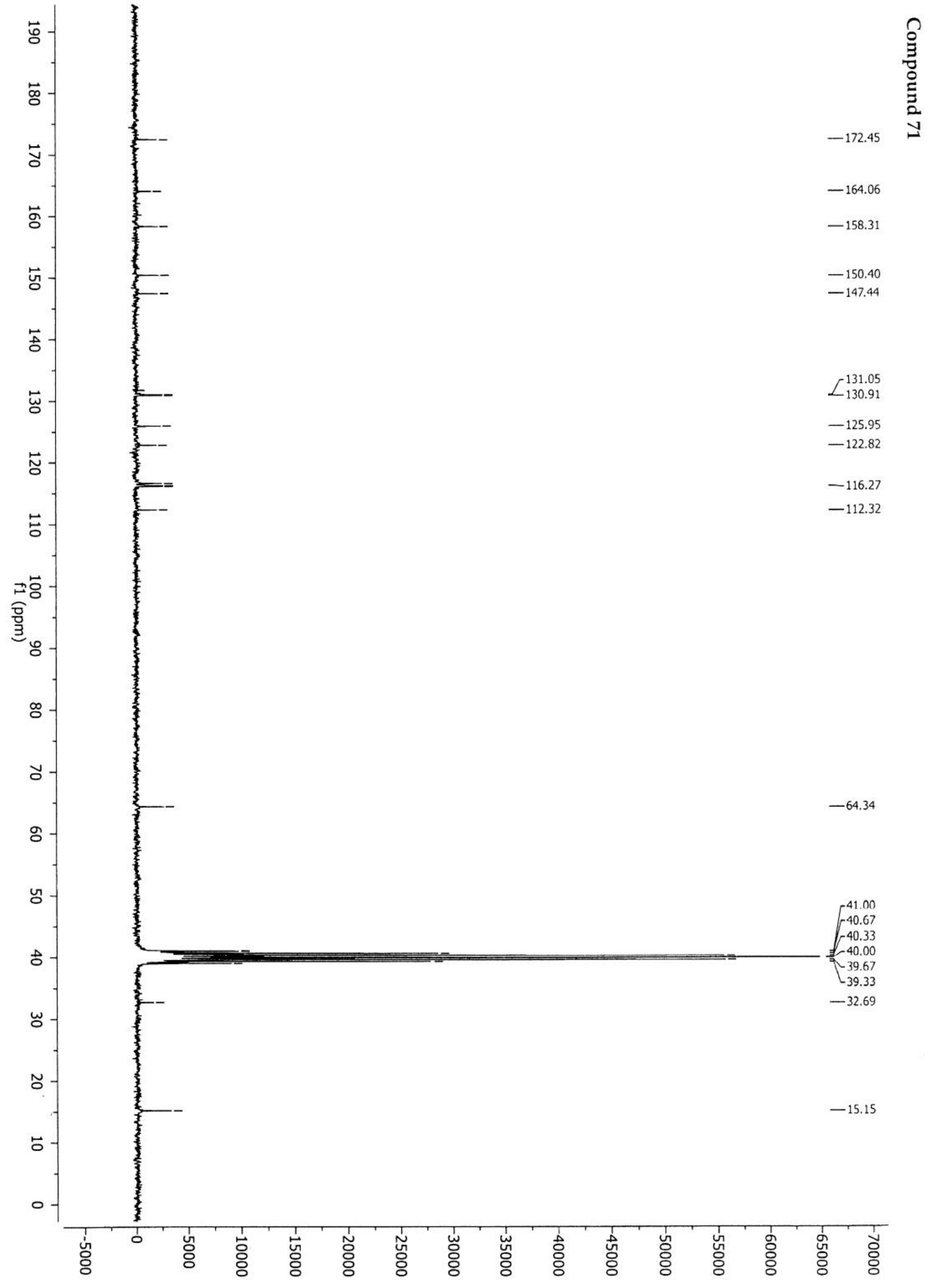

Fig. S52. ^{13}C NMR spectrum for compound **71**

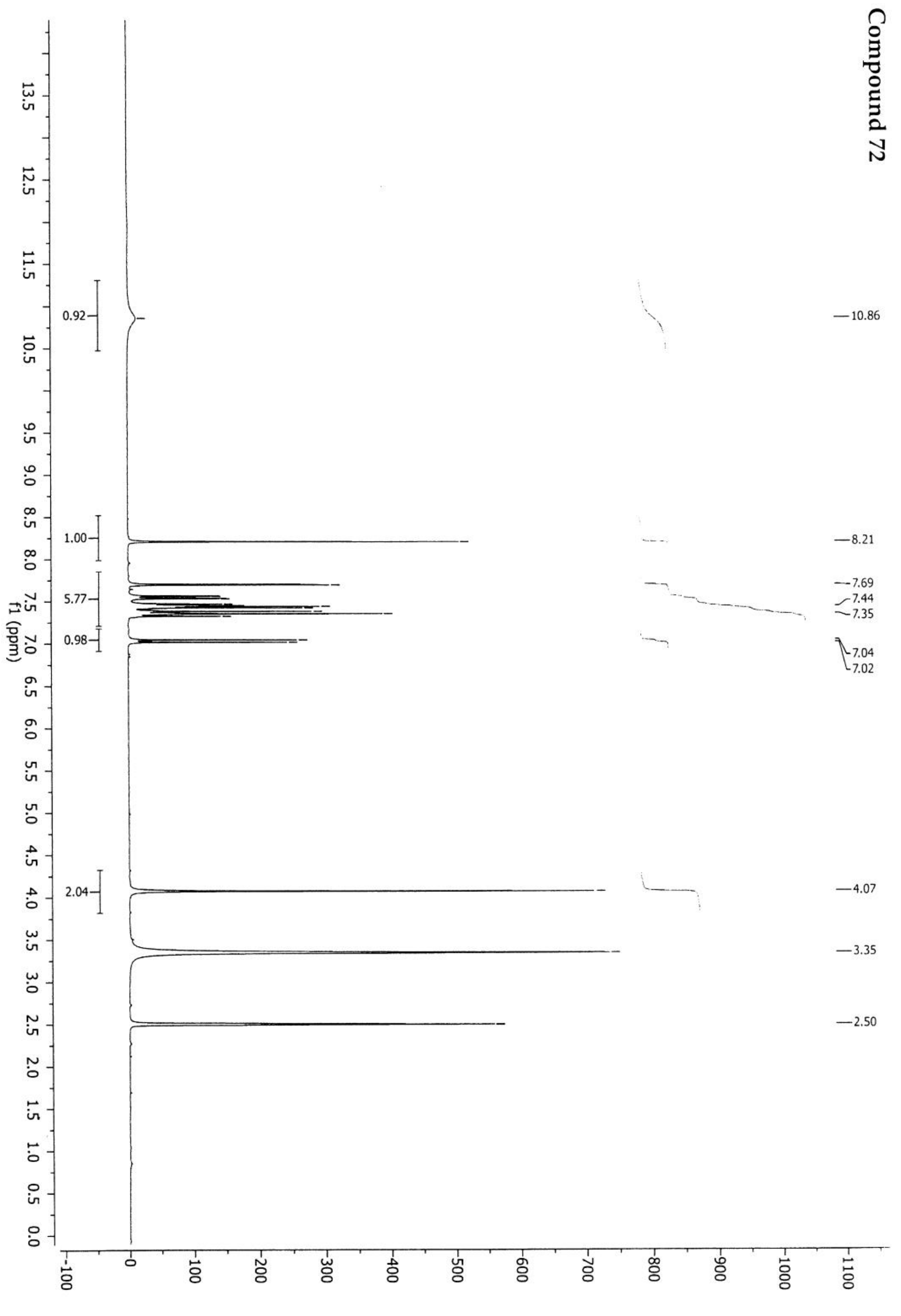

Fig. S53. ¹H NMR spectrum for compound **72**

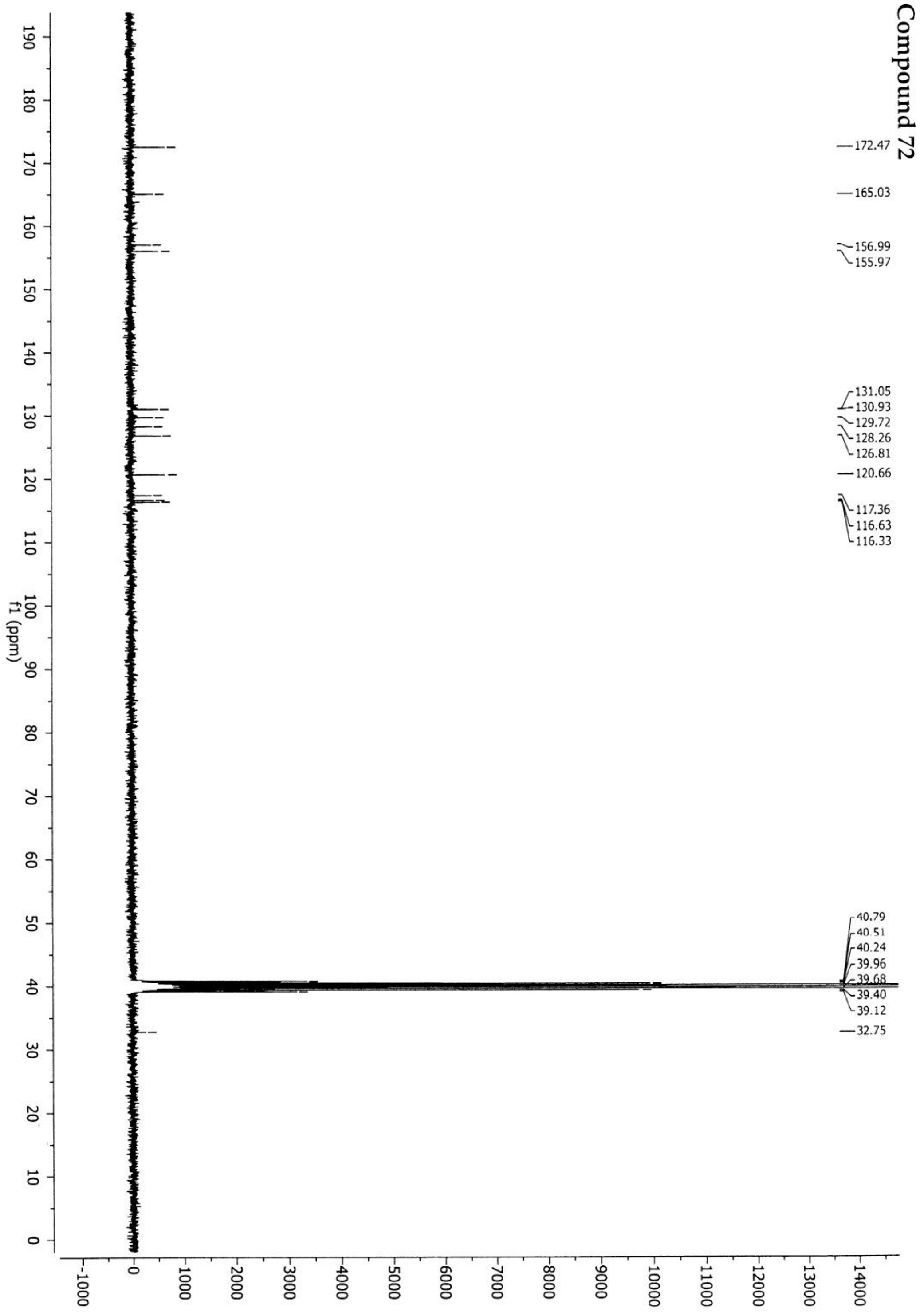

Fig. S54. ^{13}C NMR spectrum for compound **72**

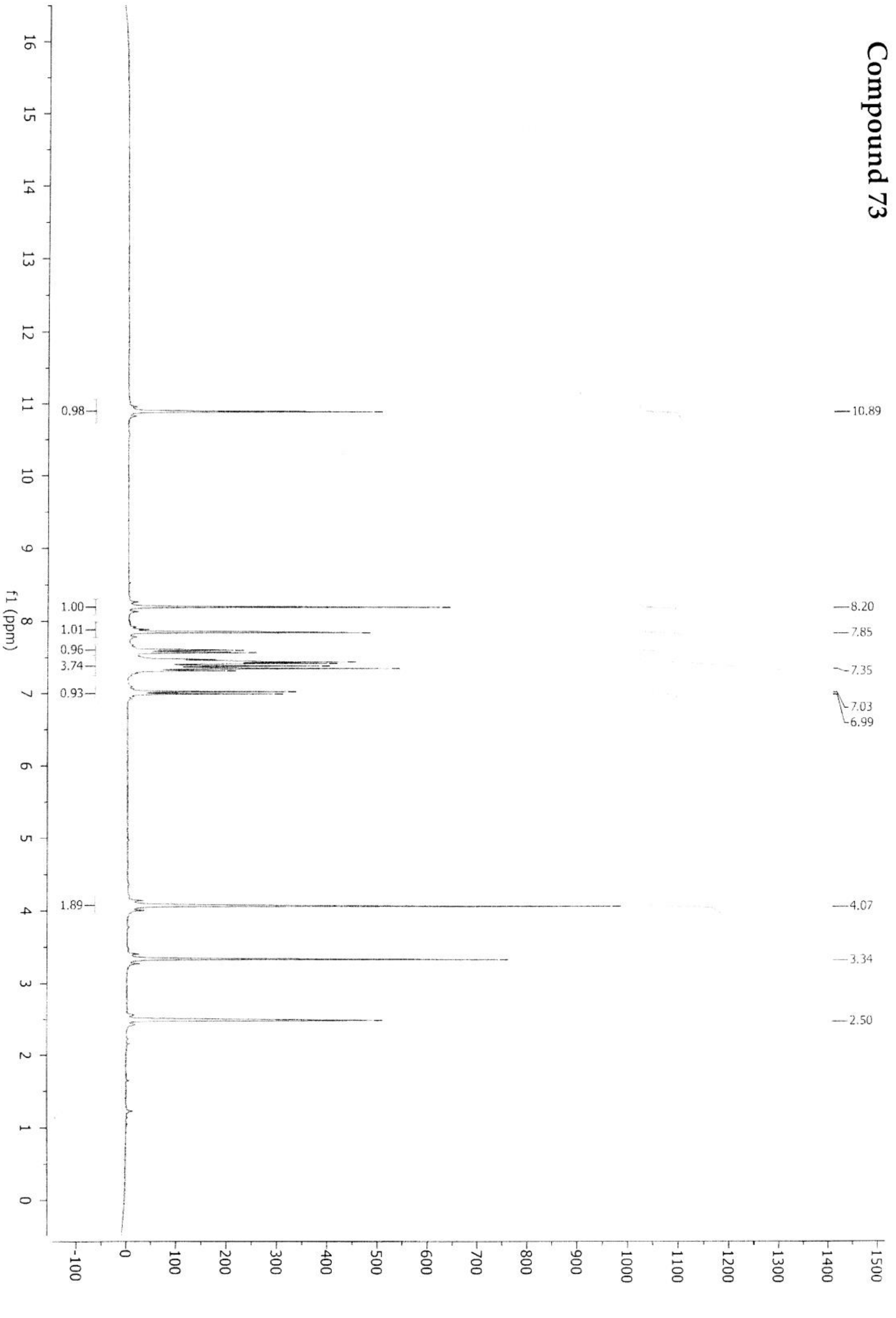

Fig. S55. ^{1}H NMR spectrum for compound **73**

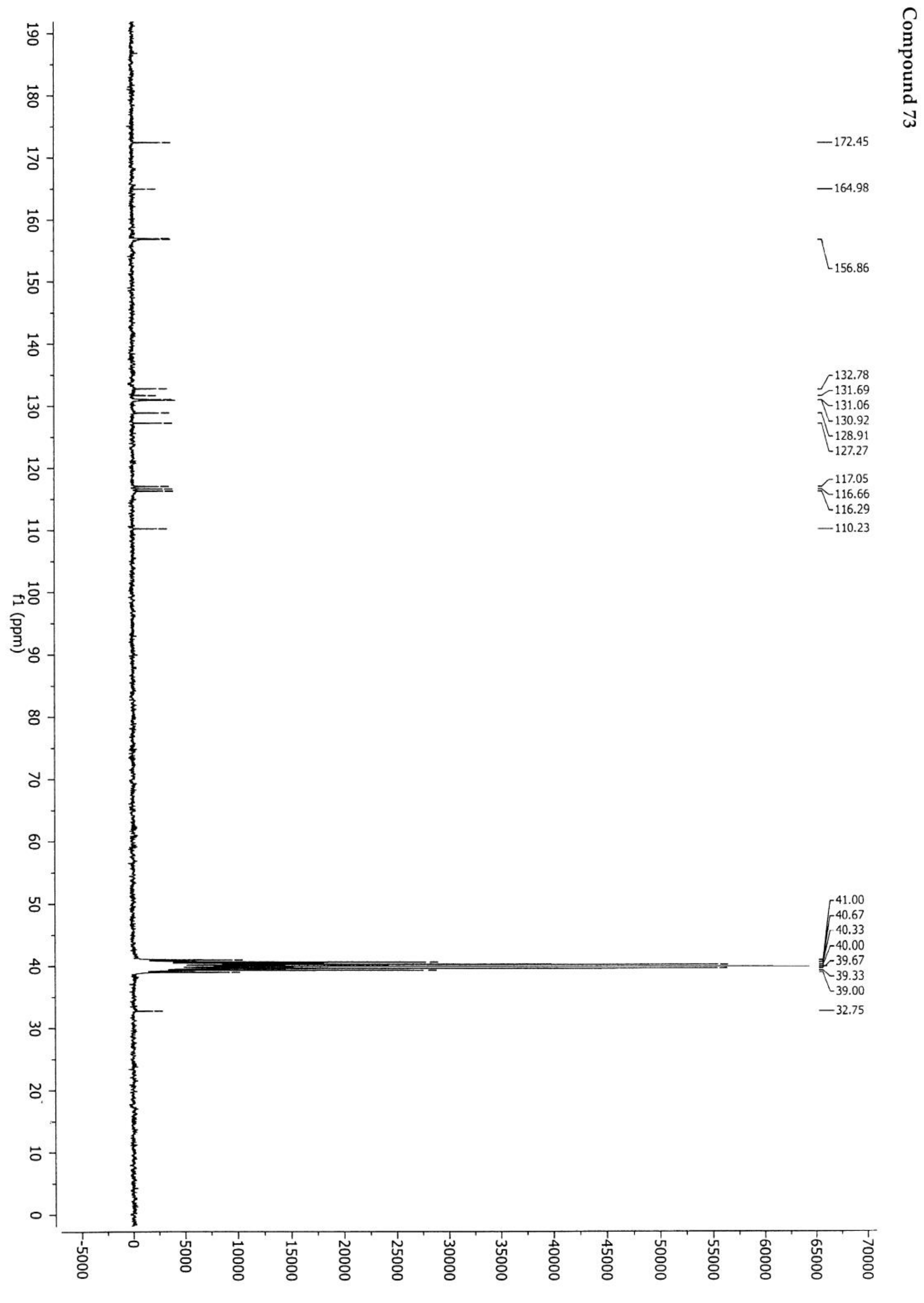

Fig. S56. ^{13}C NMR spectrum for compound **73**

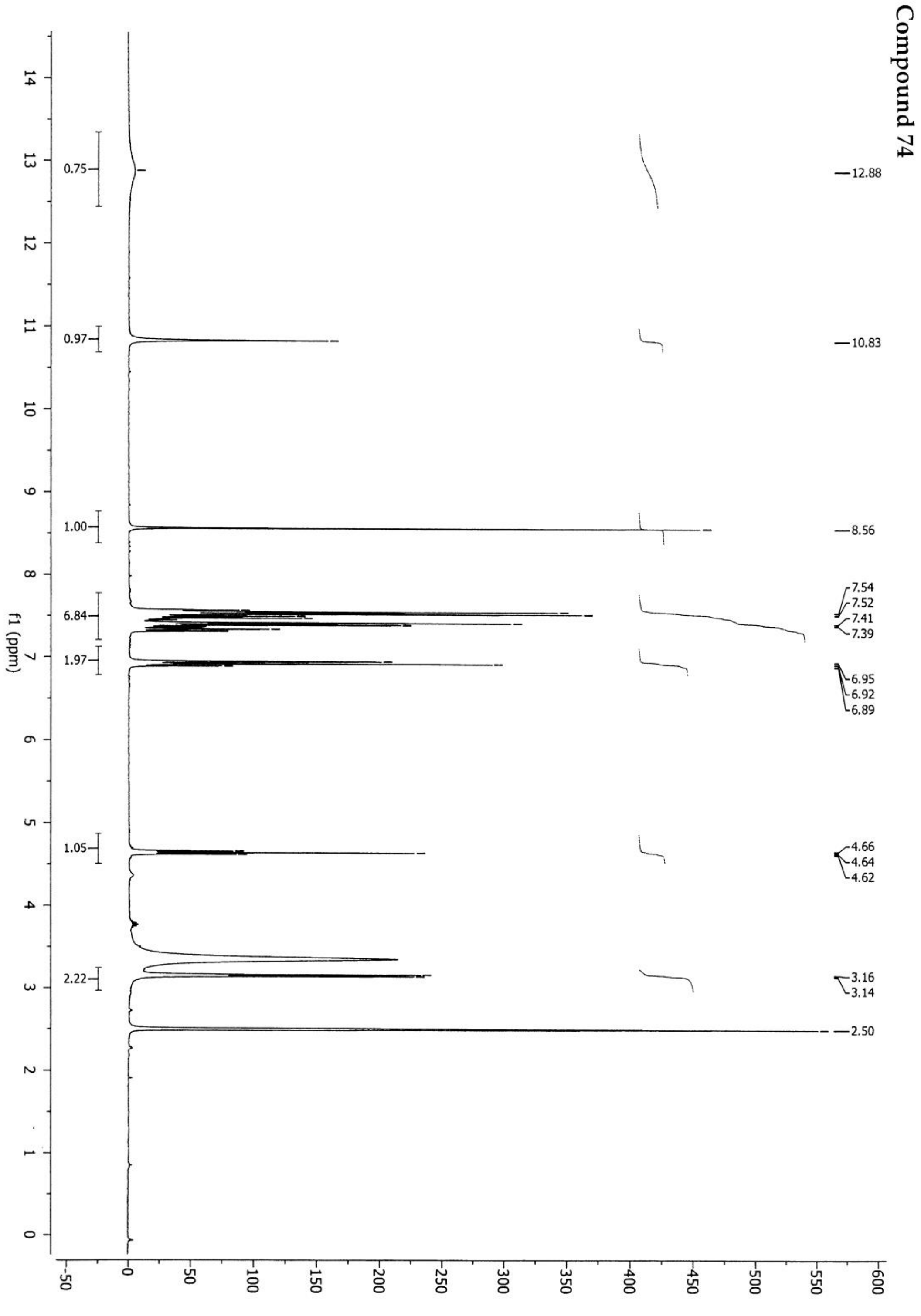

Fig. S57. ^{1}H NMR spectrum for compound **74**

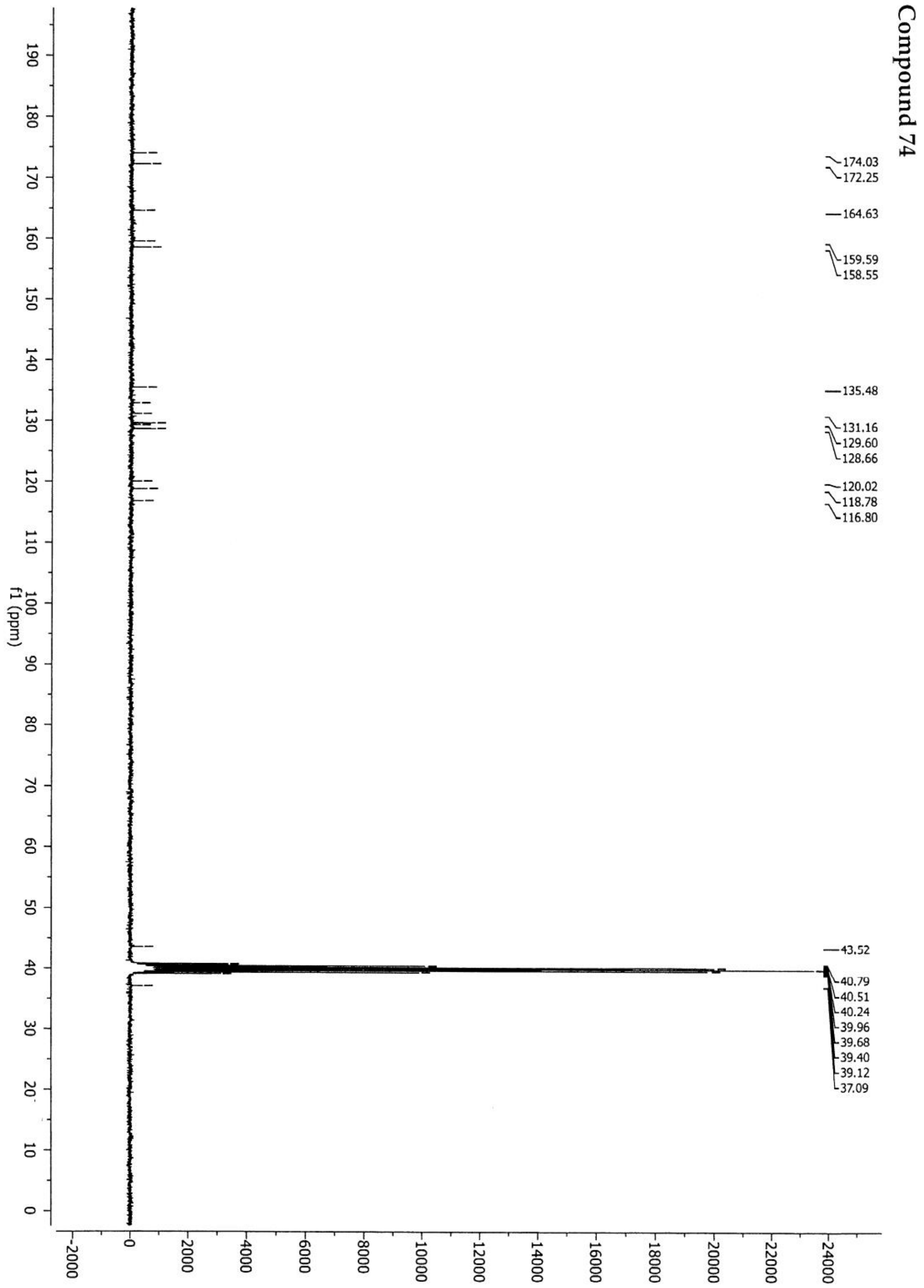

Fig. S58. ^{13}C NMR spectrum for compound **74**

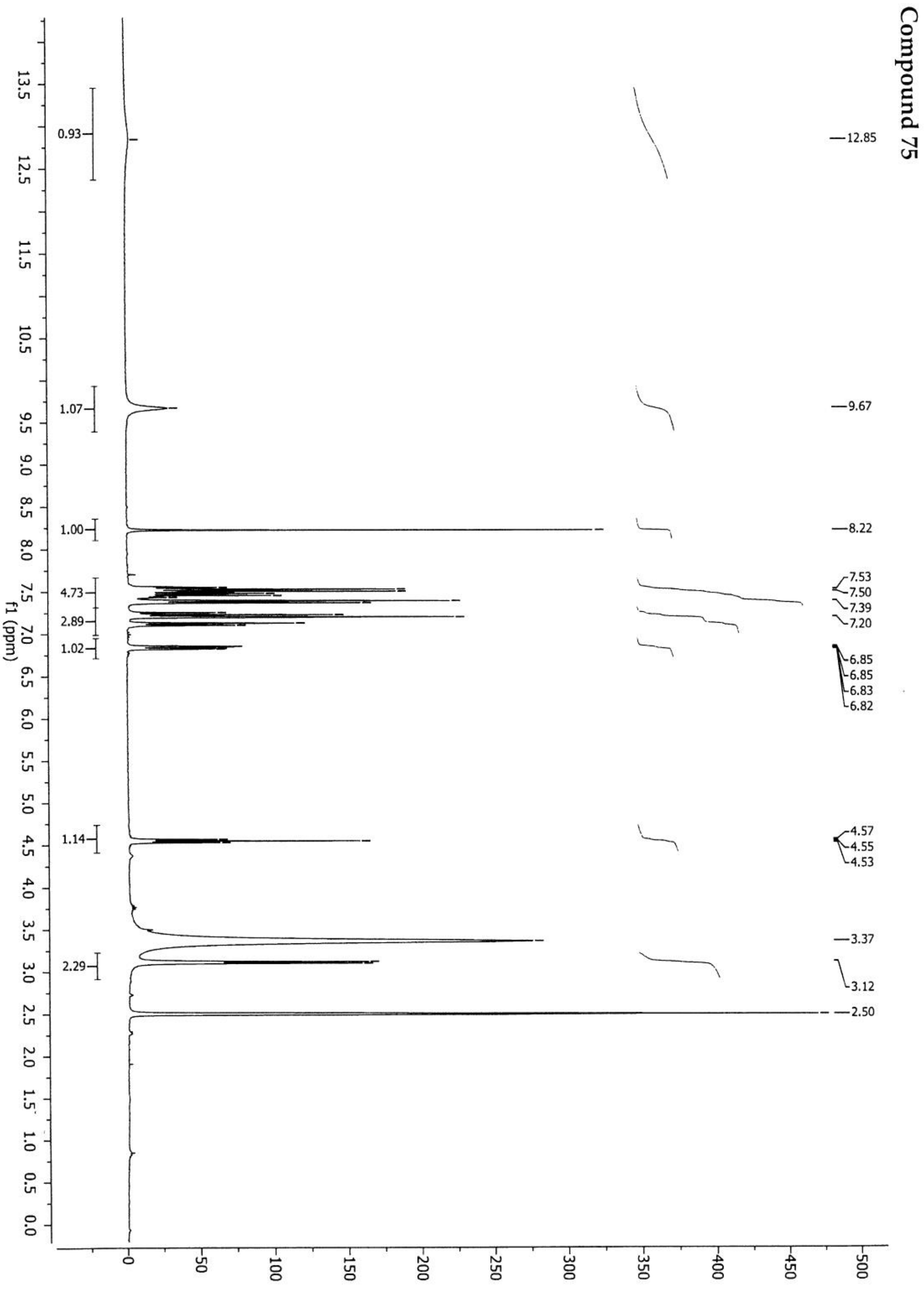

Fig. S59. ^{1}H NMR spectrum for compound **75**

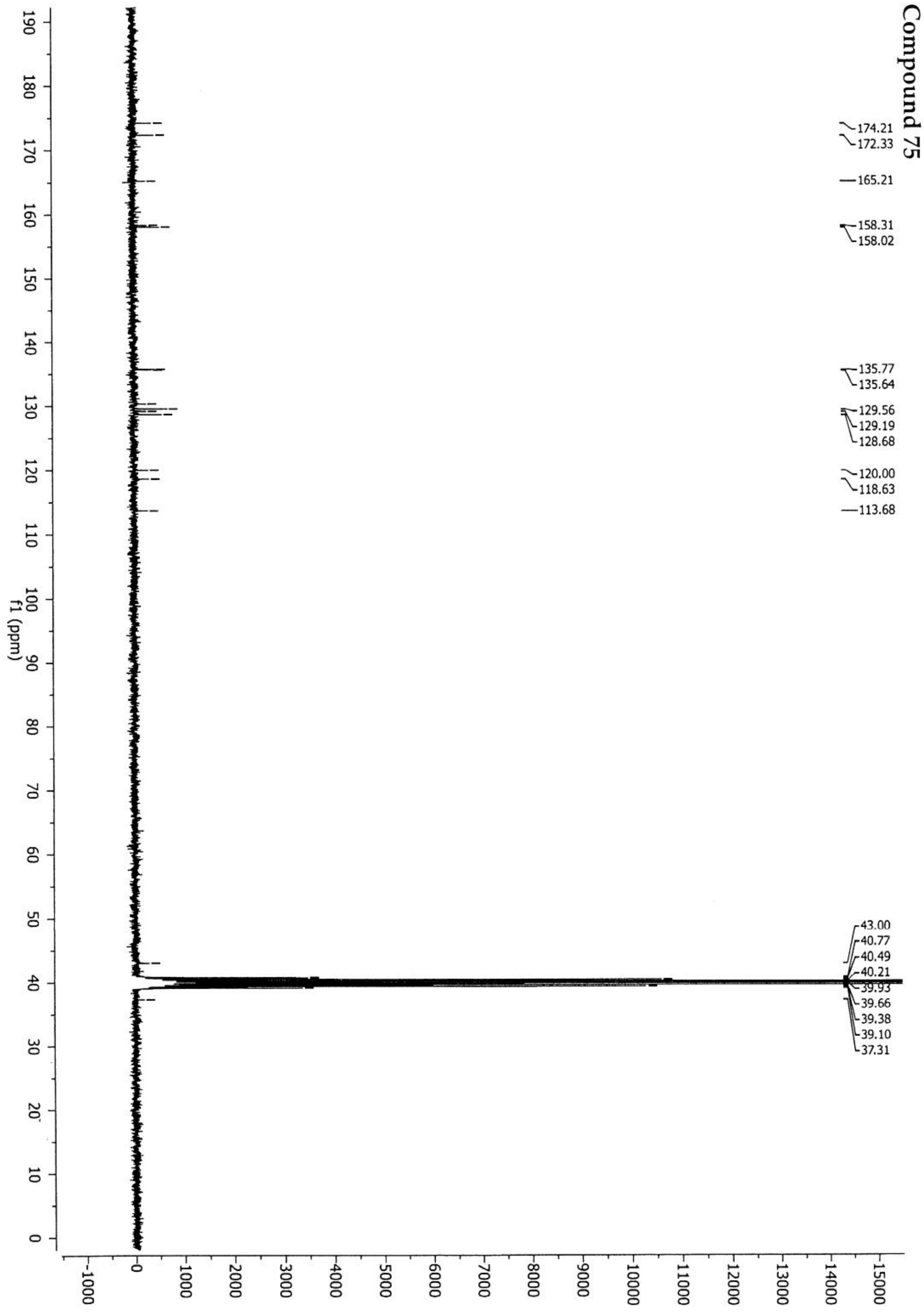

Fig. S60. ^{13}C NMR spectrum for compound **75**

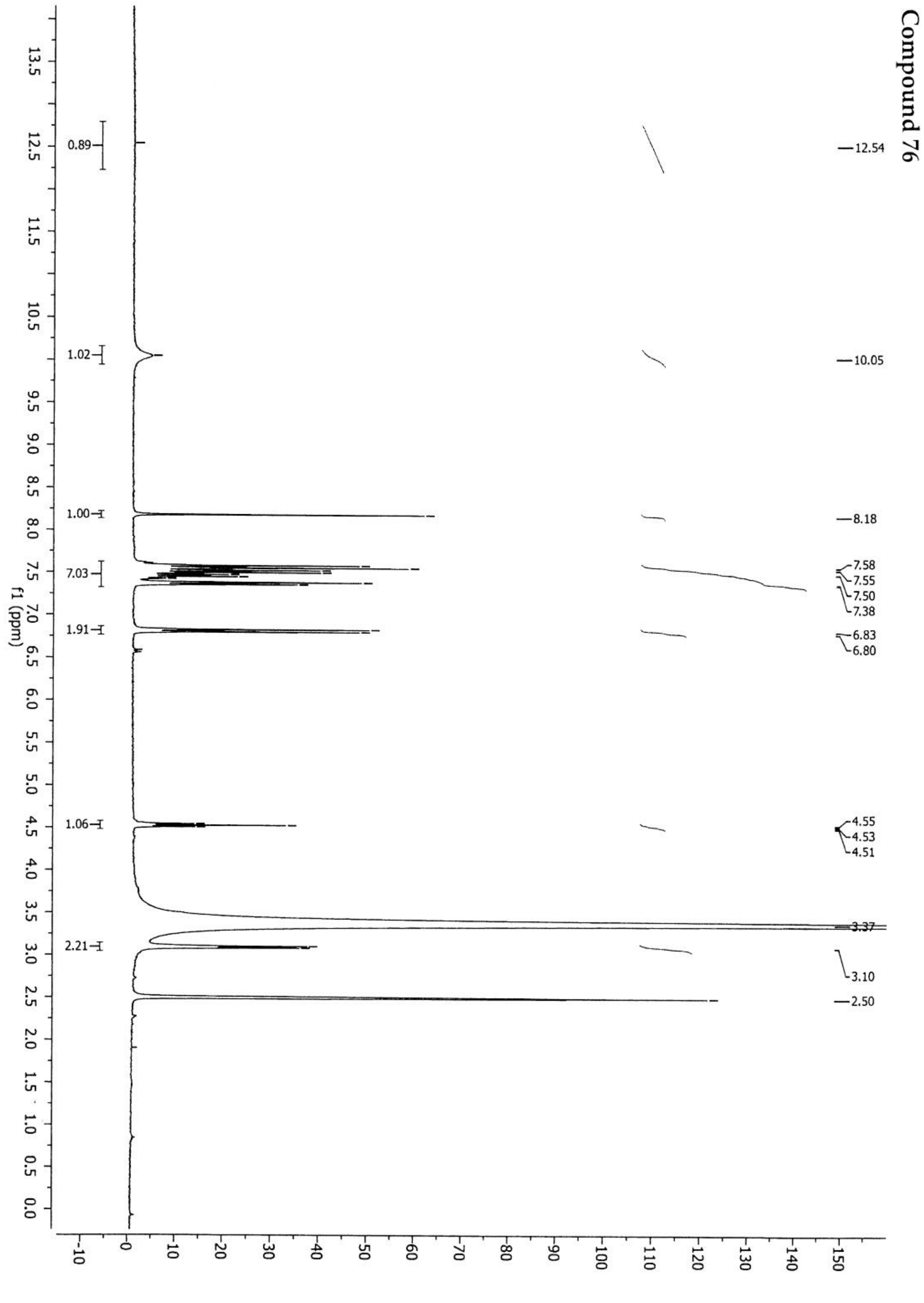

Fig. S61. ¹H NMR spectrum for compound **76**

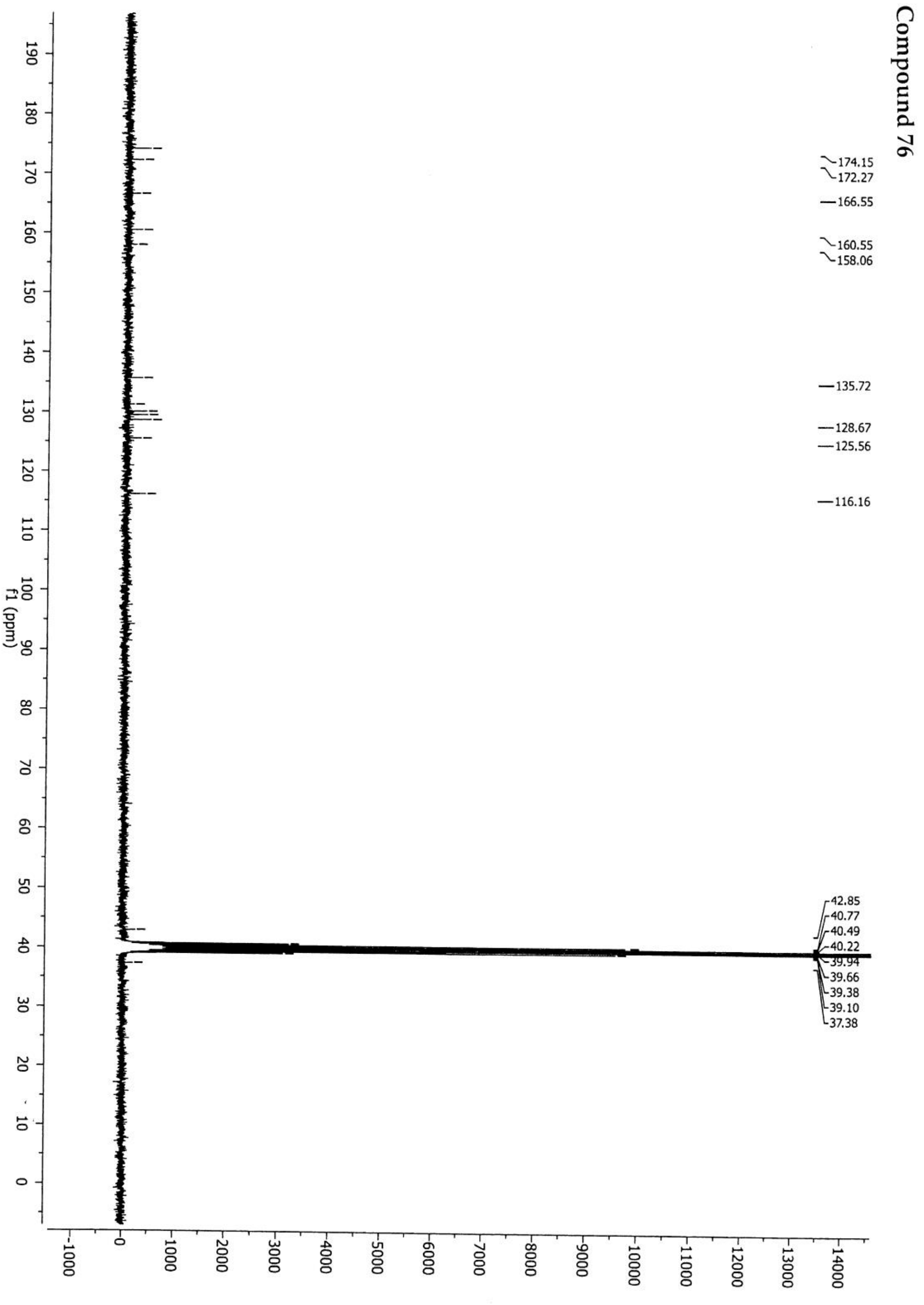

Fig. S62. ^{13}C NMR spectrum for compound **76**

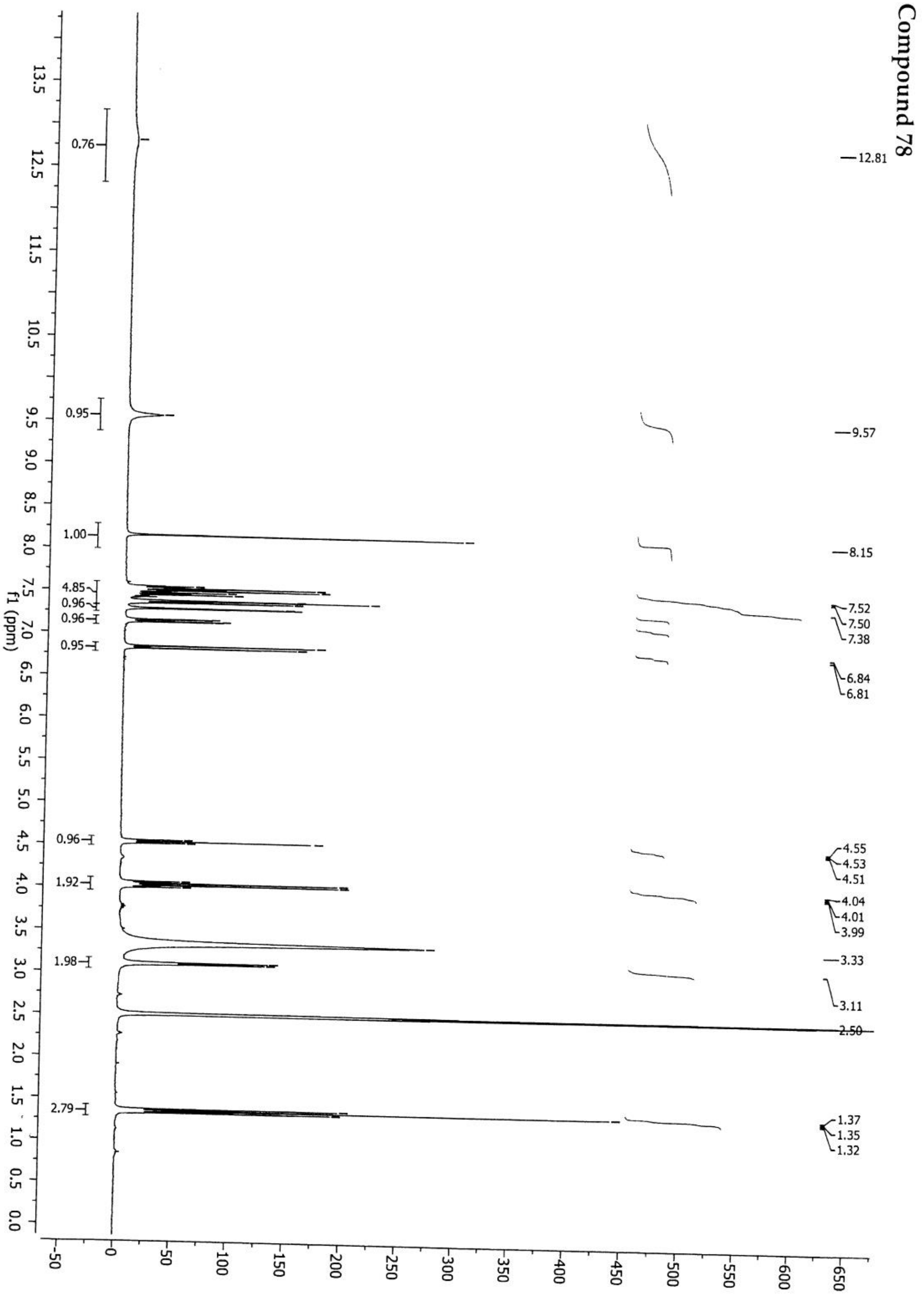

Fig. S63. ^{1}H NMR spectrum for compound **78**

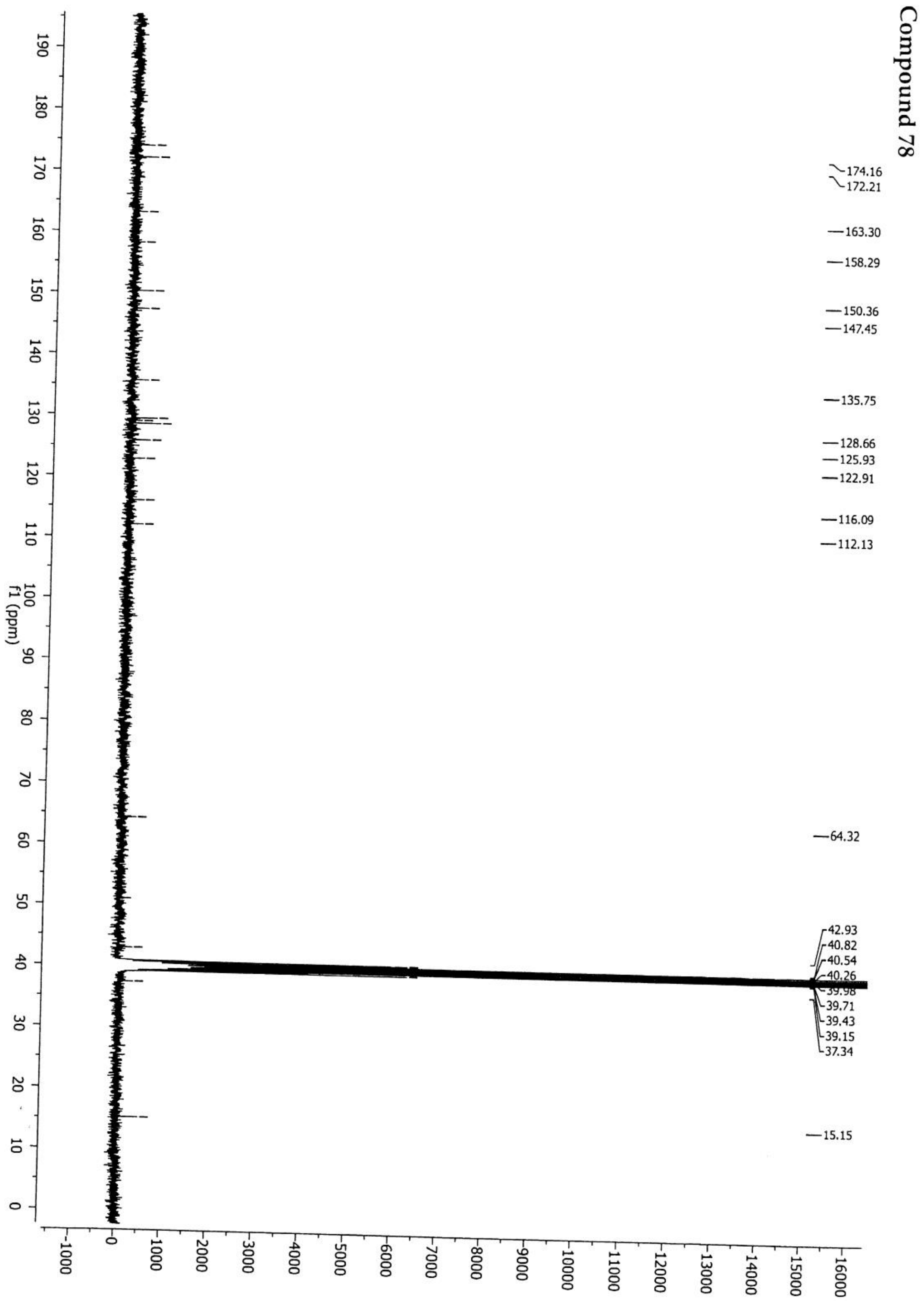

Fig. S64. ^{13}C NMR spectrum for compound **78**

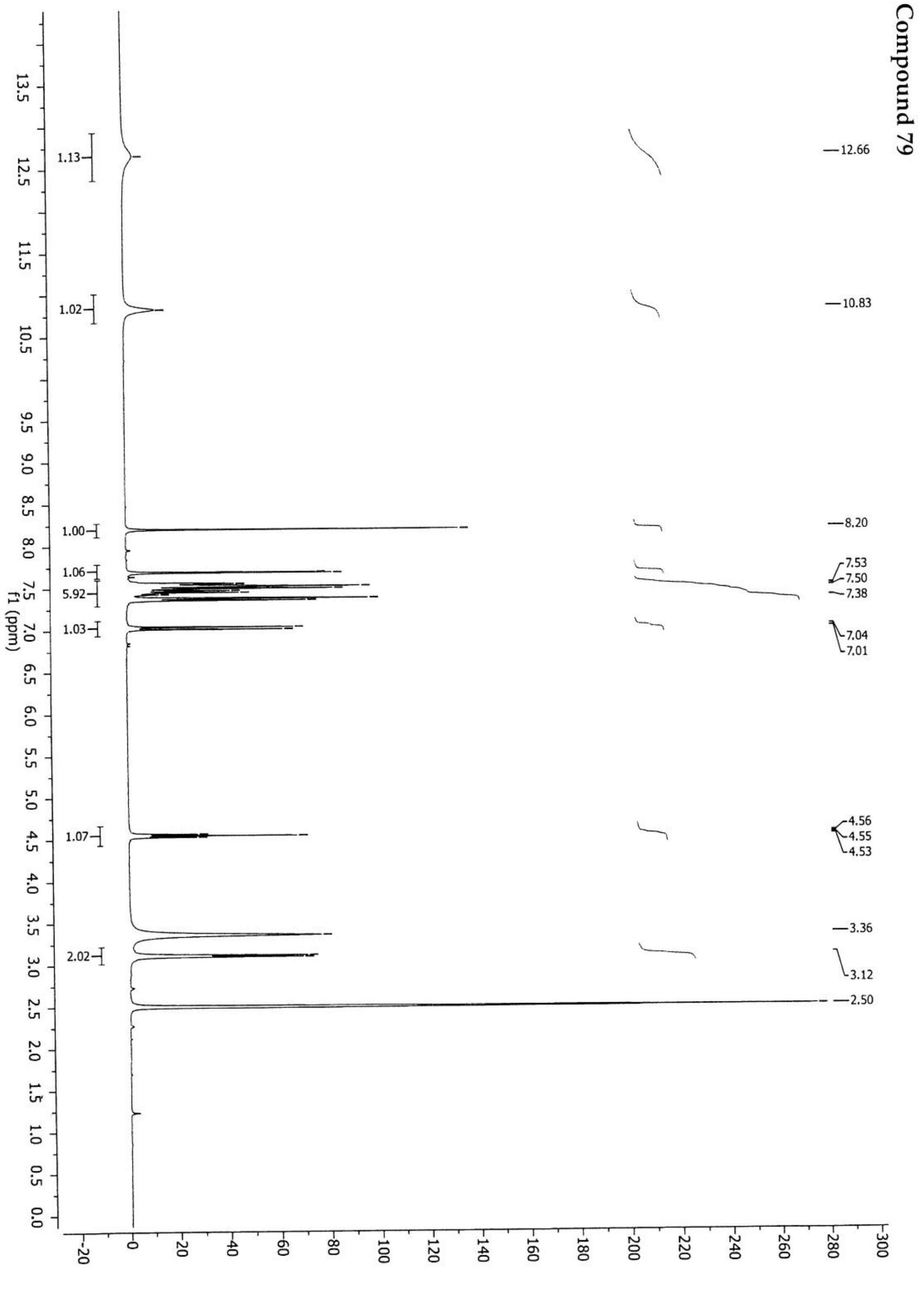

Fig. S65. ^1H NMR spectrum for compound **79**

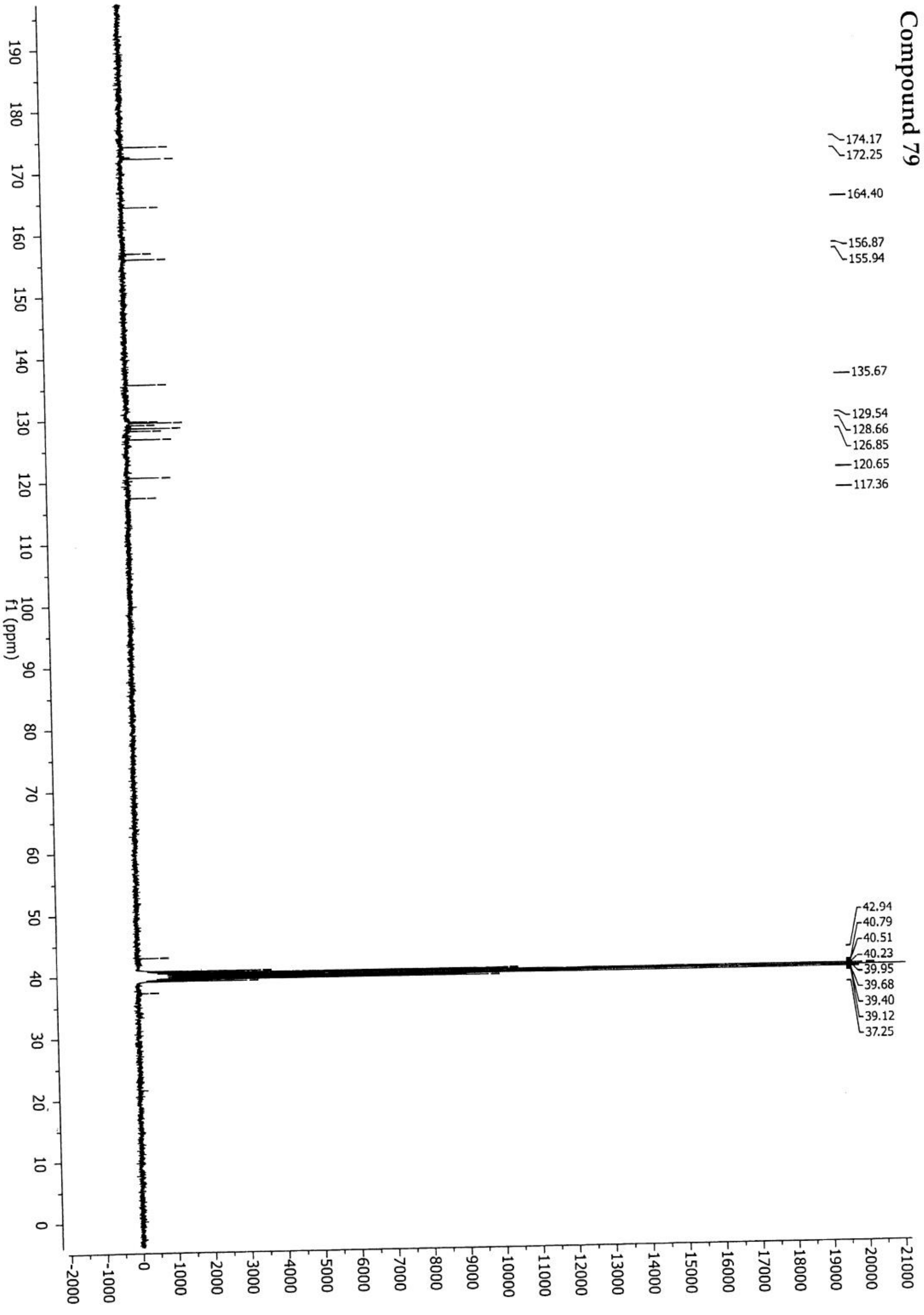

Fig. S66. ^{13}C NMR spectrum for compound **79**

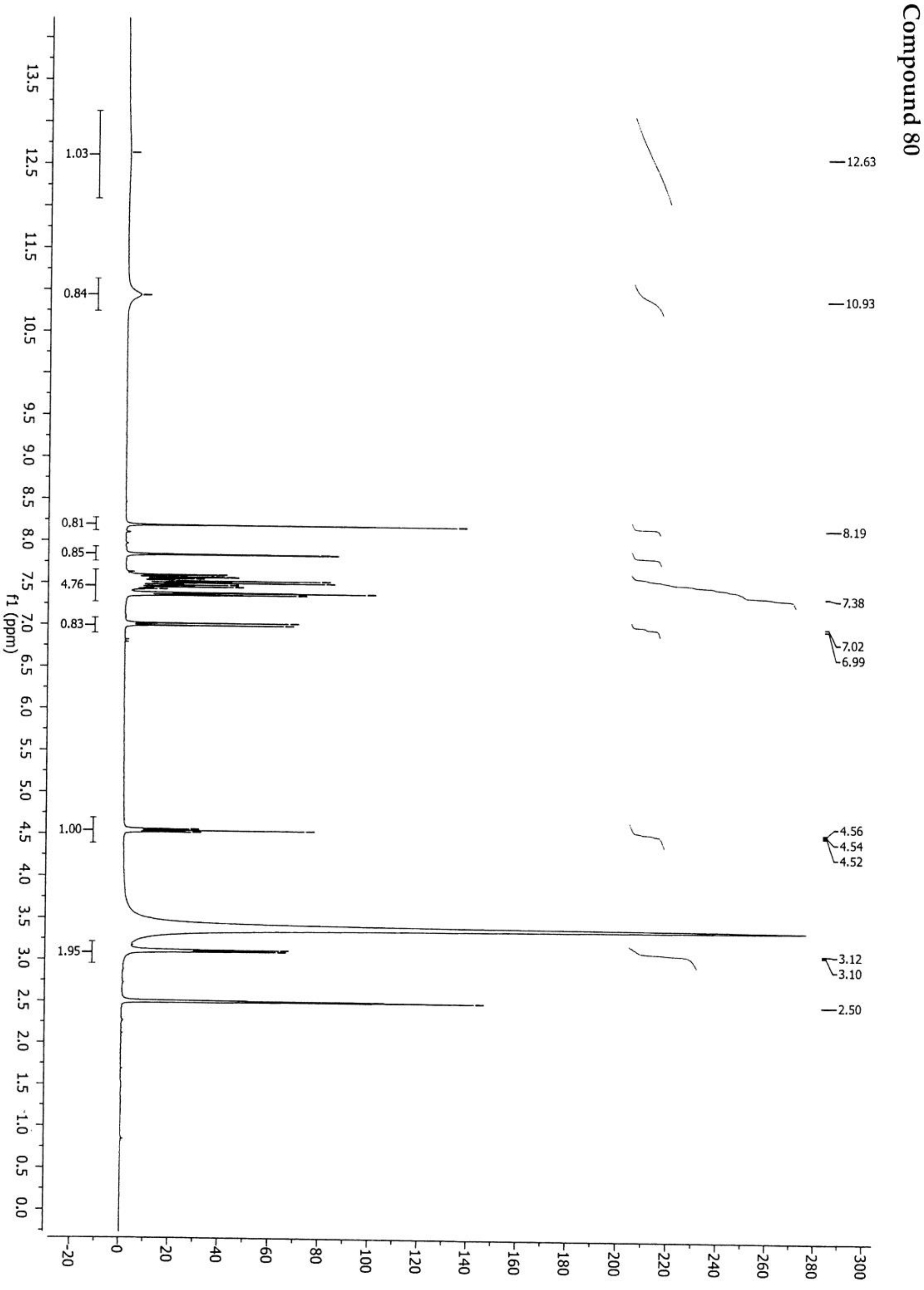

Fig. S67. ^{1}H NMR spectrum for compound **80**

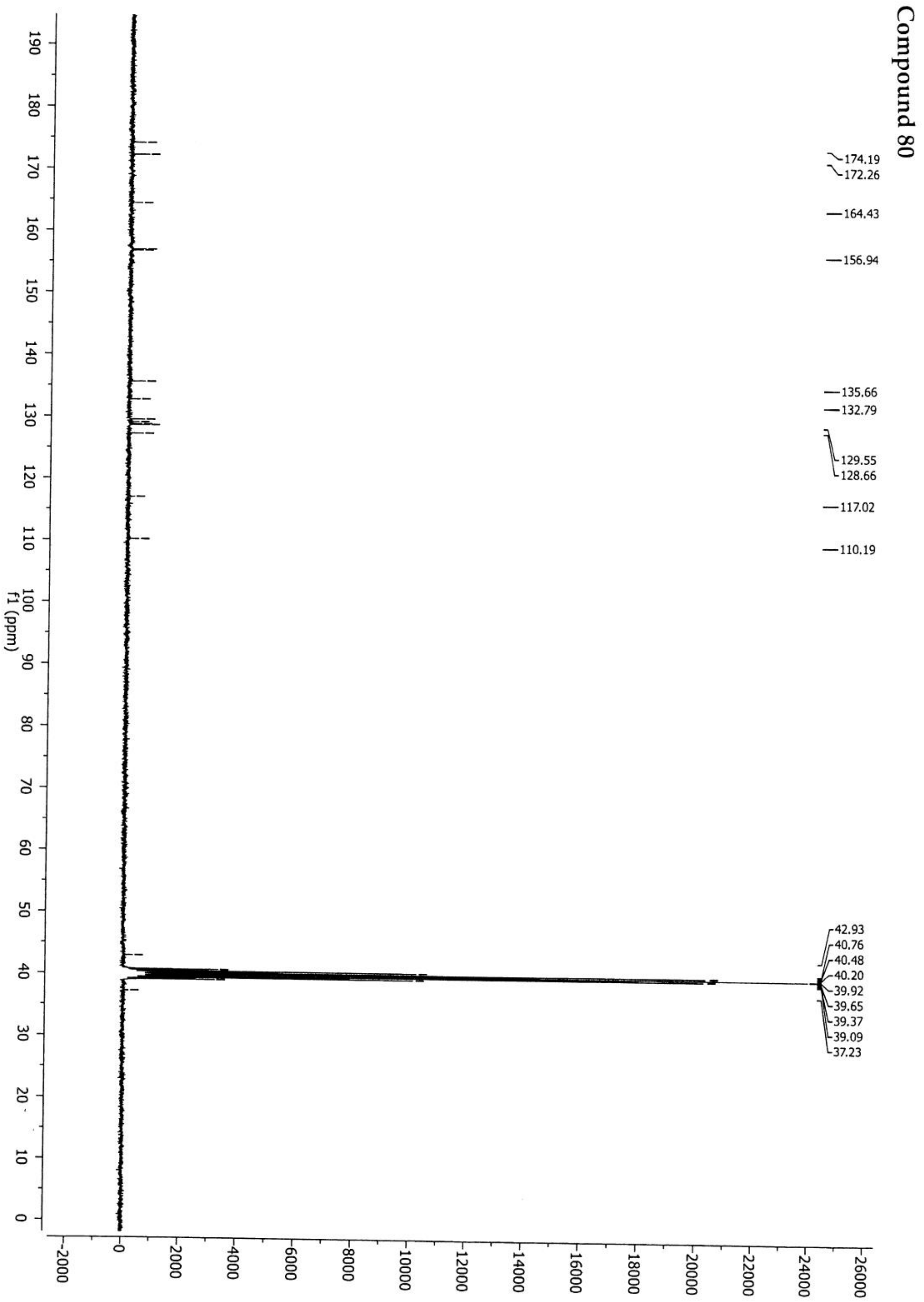

Fig. S68. ^{13}C NMR spectrum for compound **80**

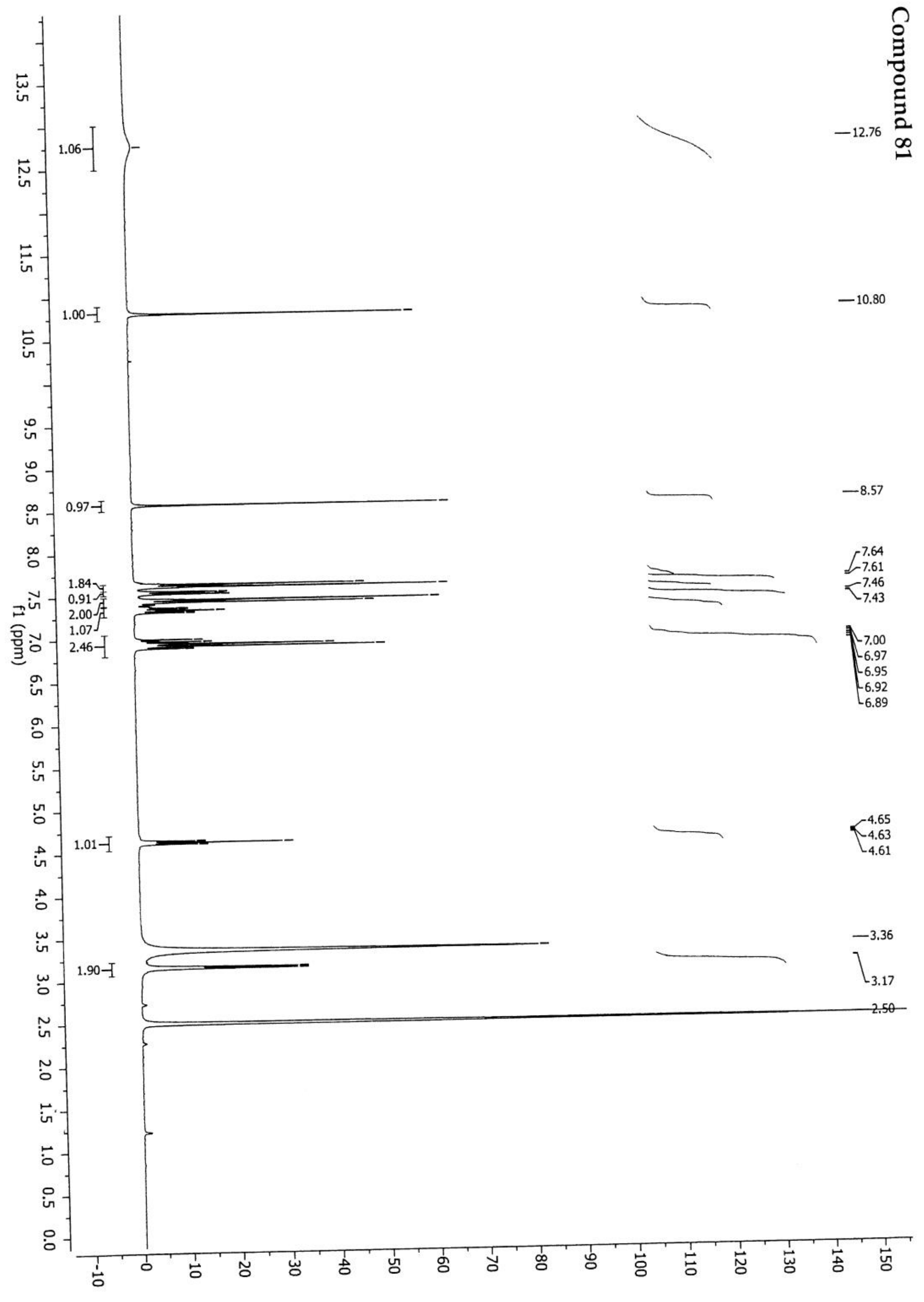

Fig. S69. ^{1}H NMR spectrum for compound **81**

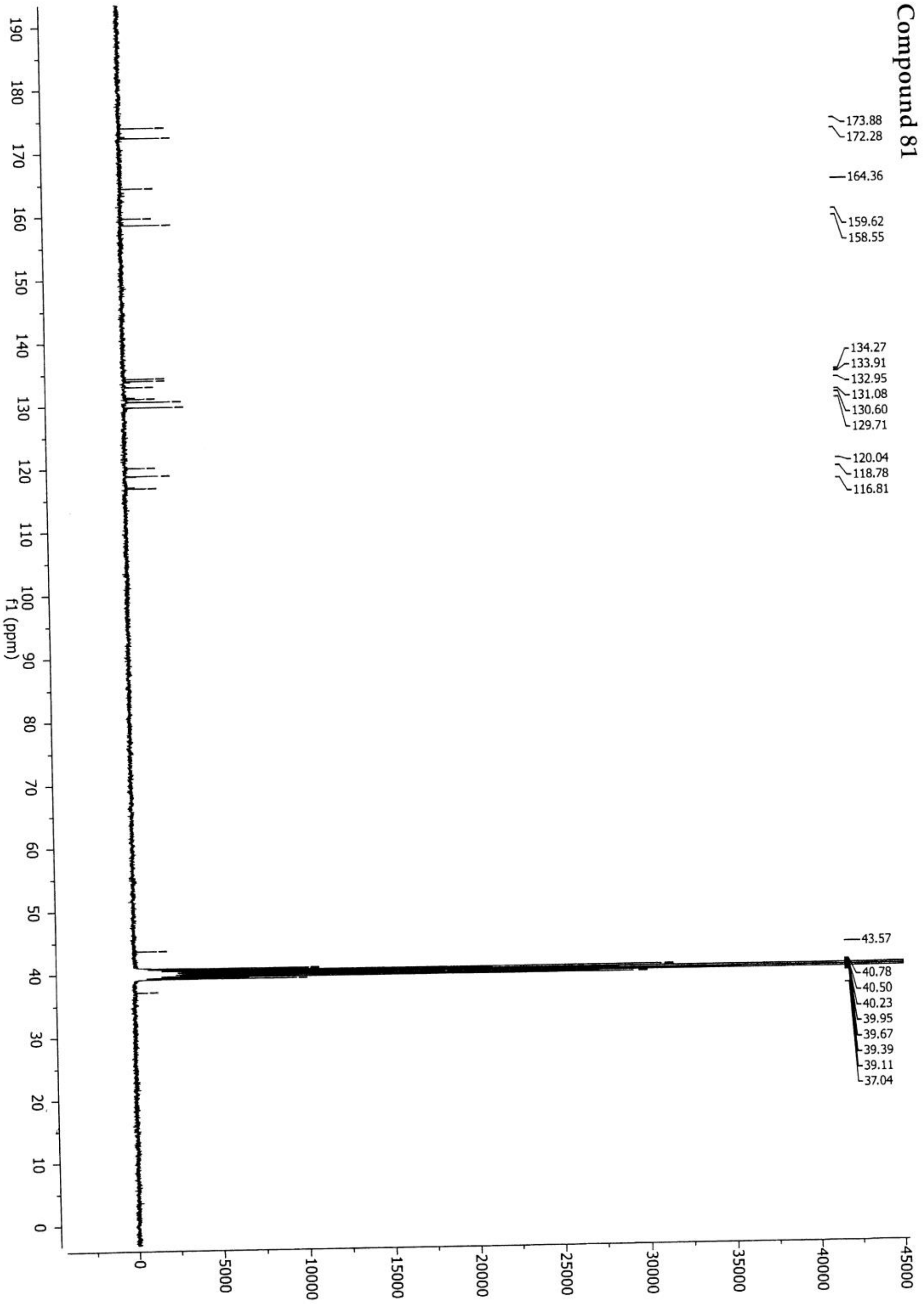

Fig. S70. ^{13}C NMR spectrum for compound **81**

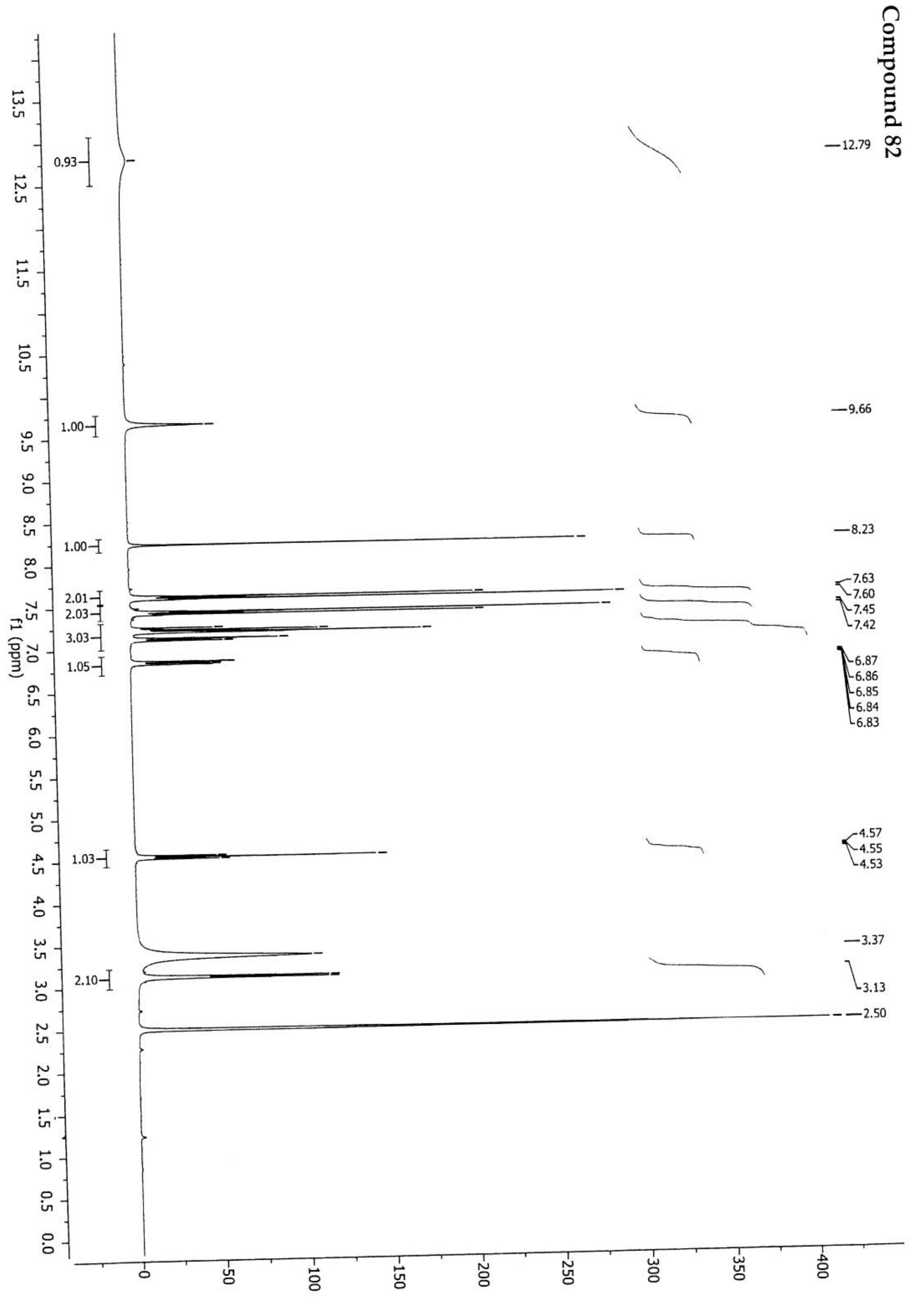

Fig. S71. ^{1}H NMR spectrum for compound **82**

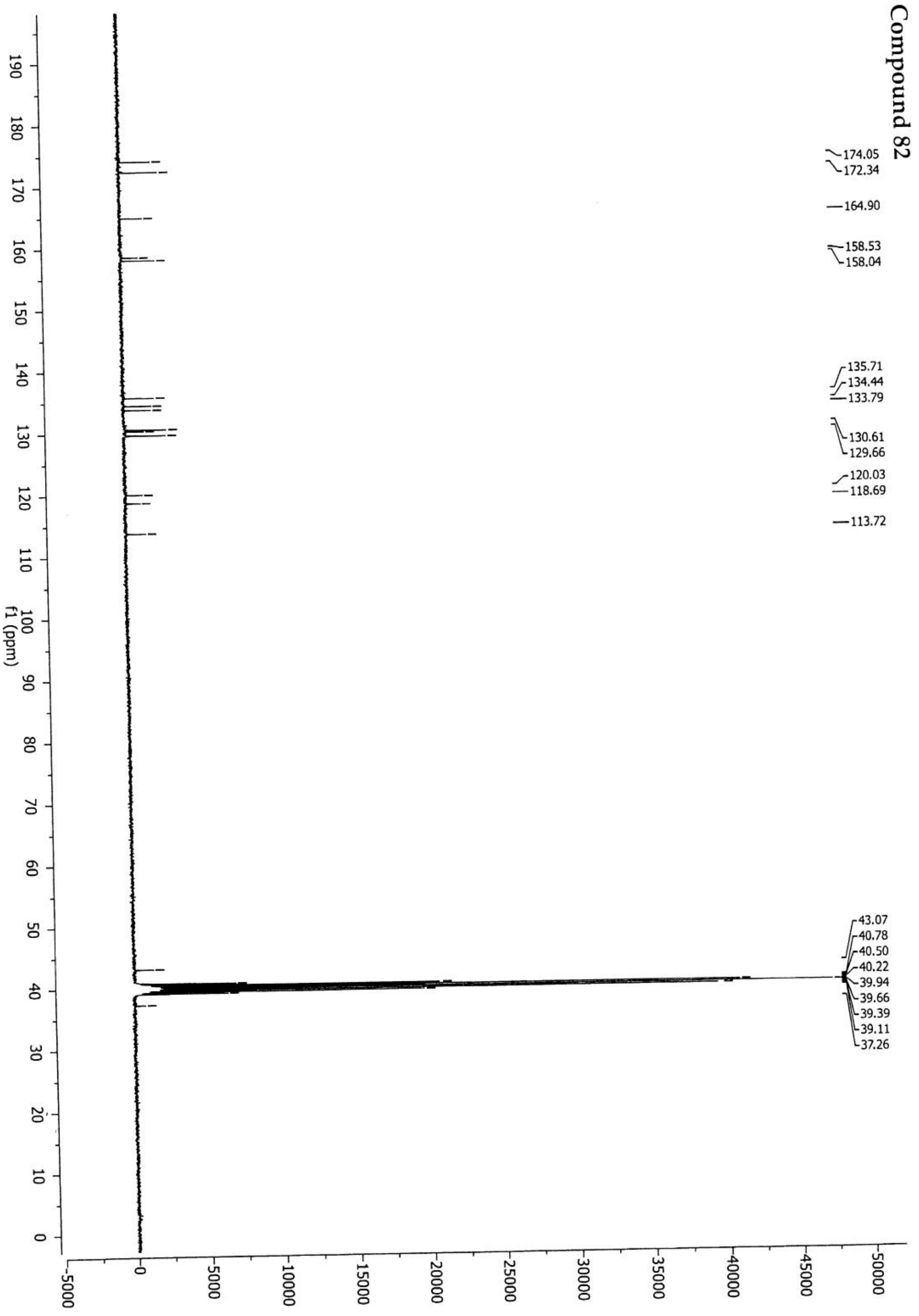

Fig. S72. ^{13}C NMR spectrum for compound **82**

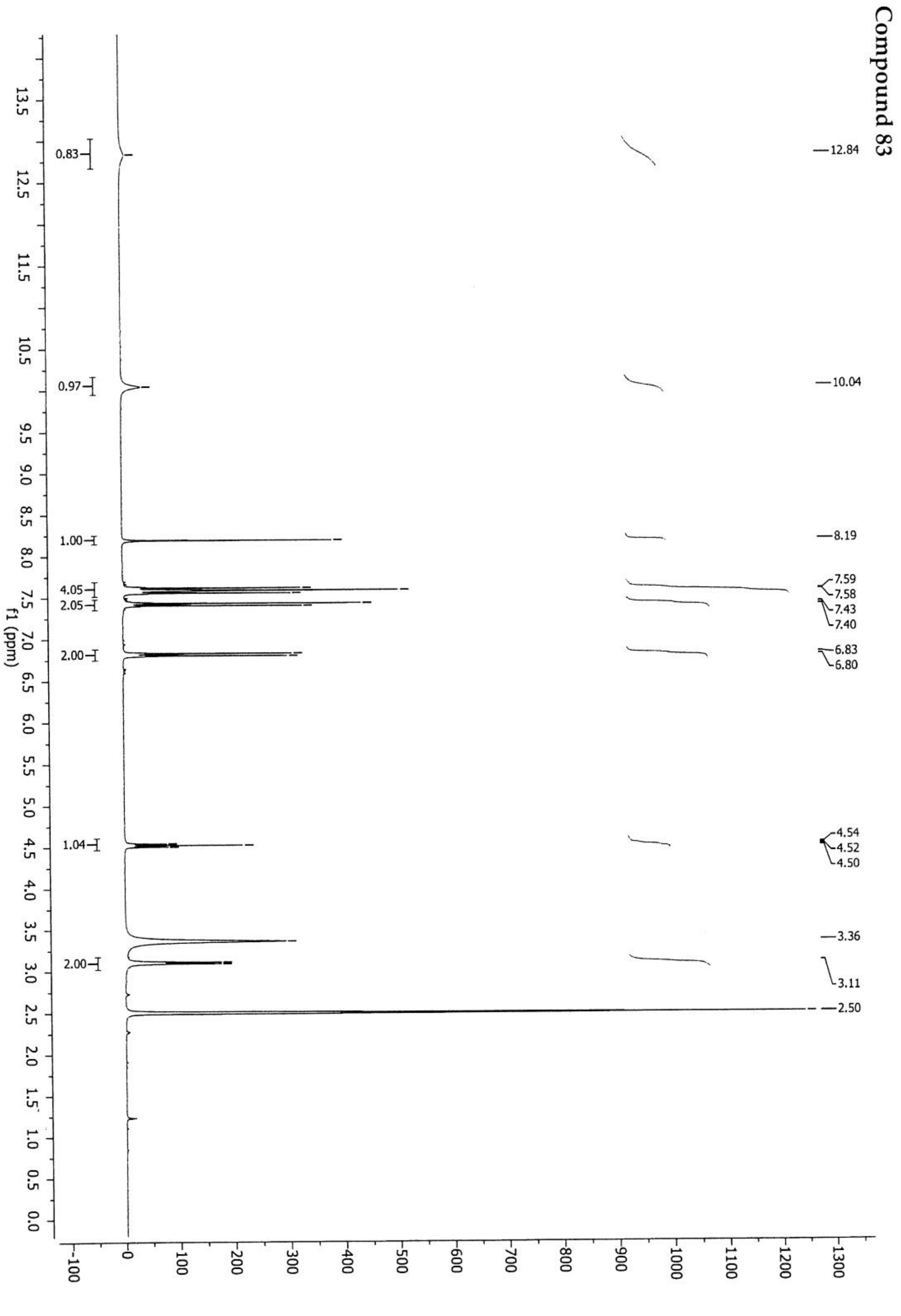

Fig. S73. ^{1}H NMR spectrum for compound **83**

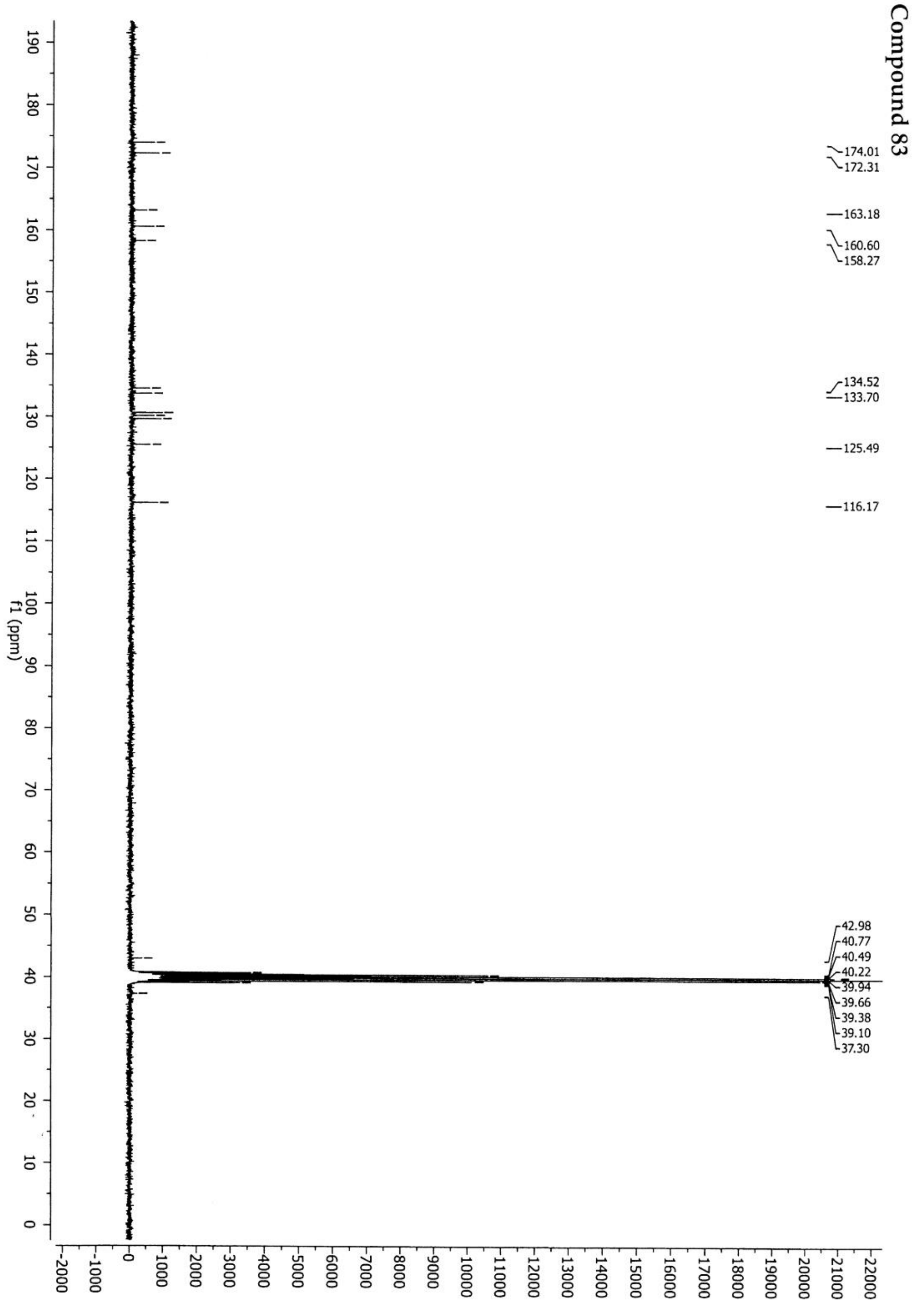

Fig. S74. ^{13}C NMR spectrum for compound **83**

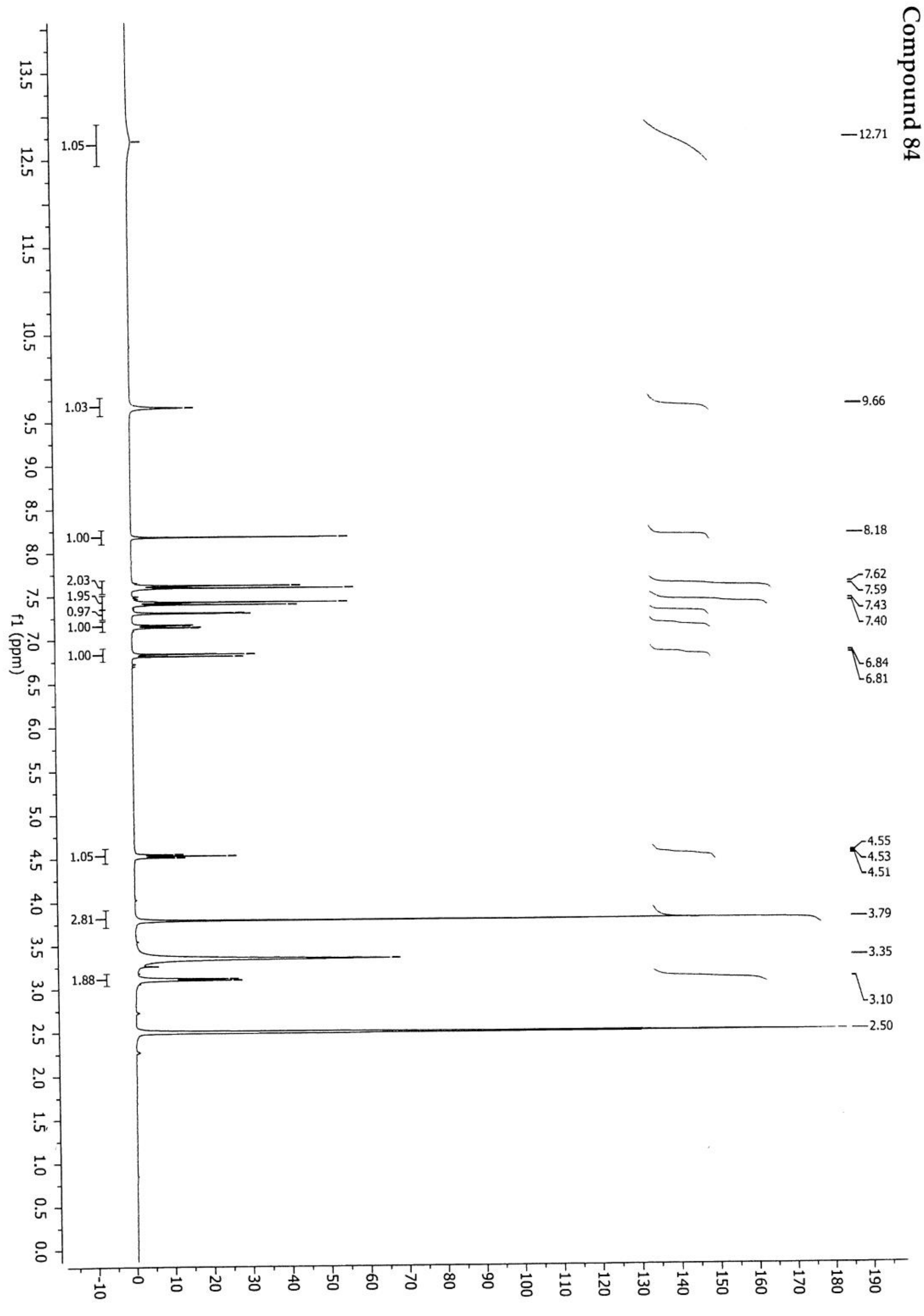

Fig. S75. ^{1}H NMR spectrum for compound **84**

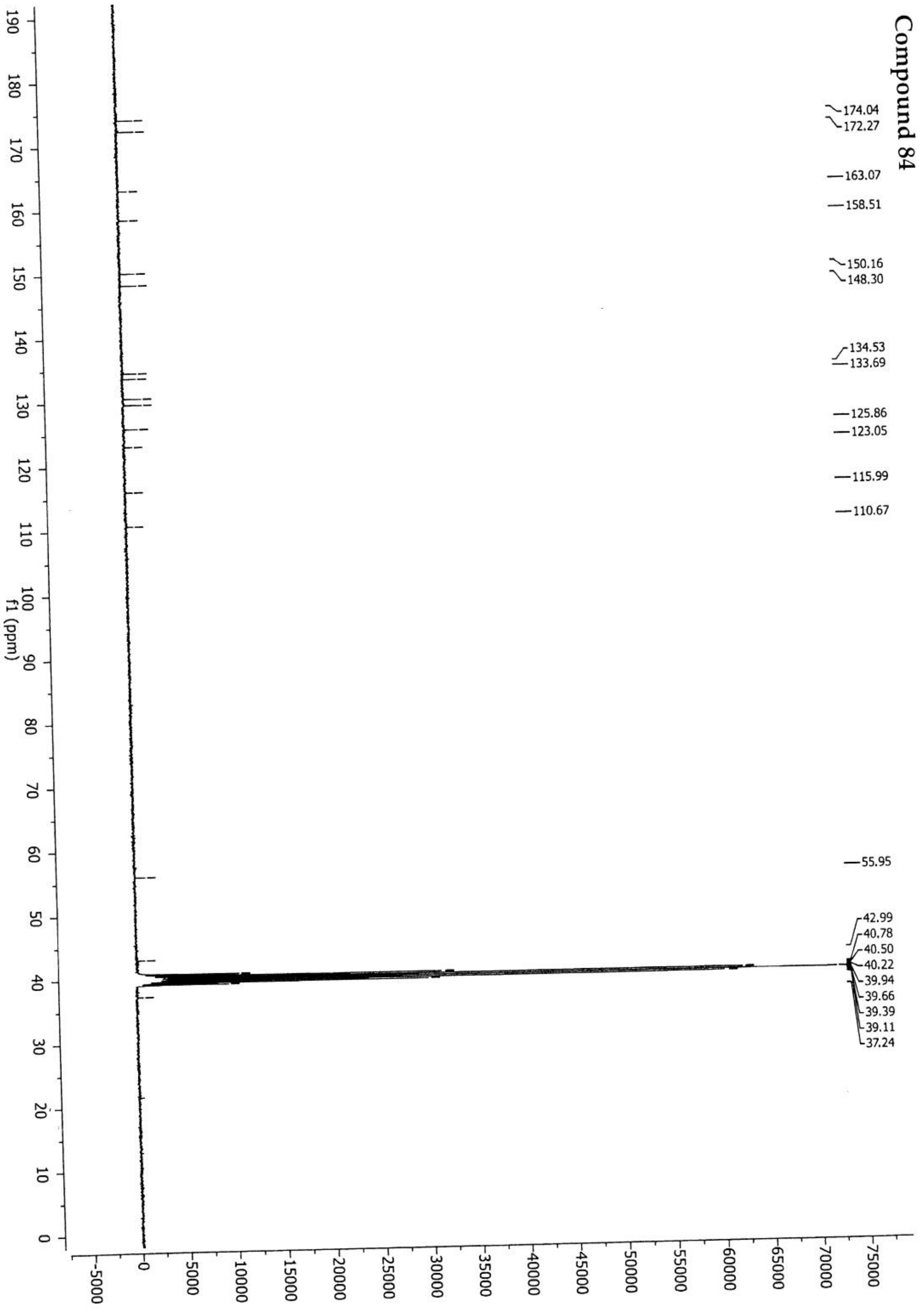

Fig. S76. ^{13}C NMR spectrum for compound **84**

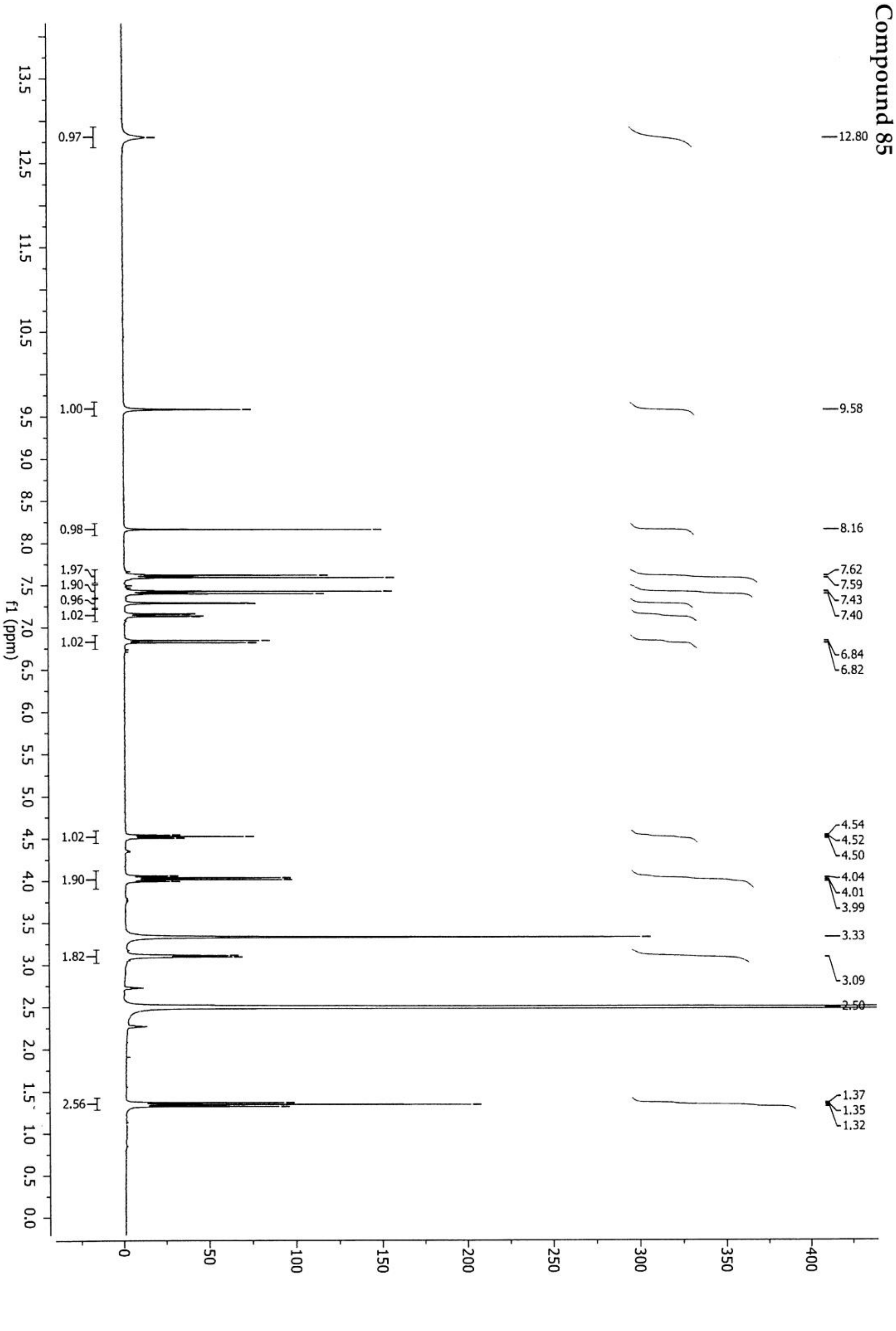

Fig. S77. ¹H NMR spectrum for compound **85**

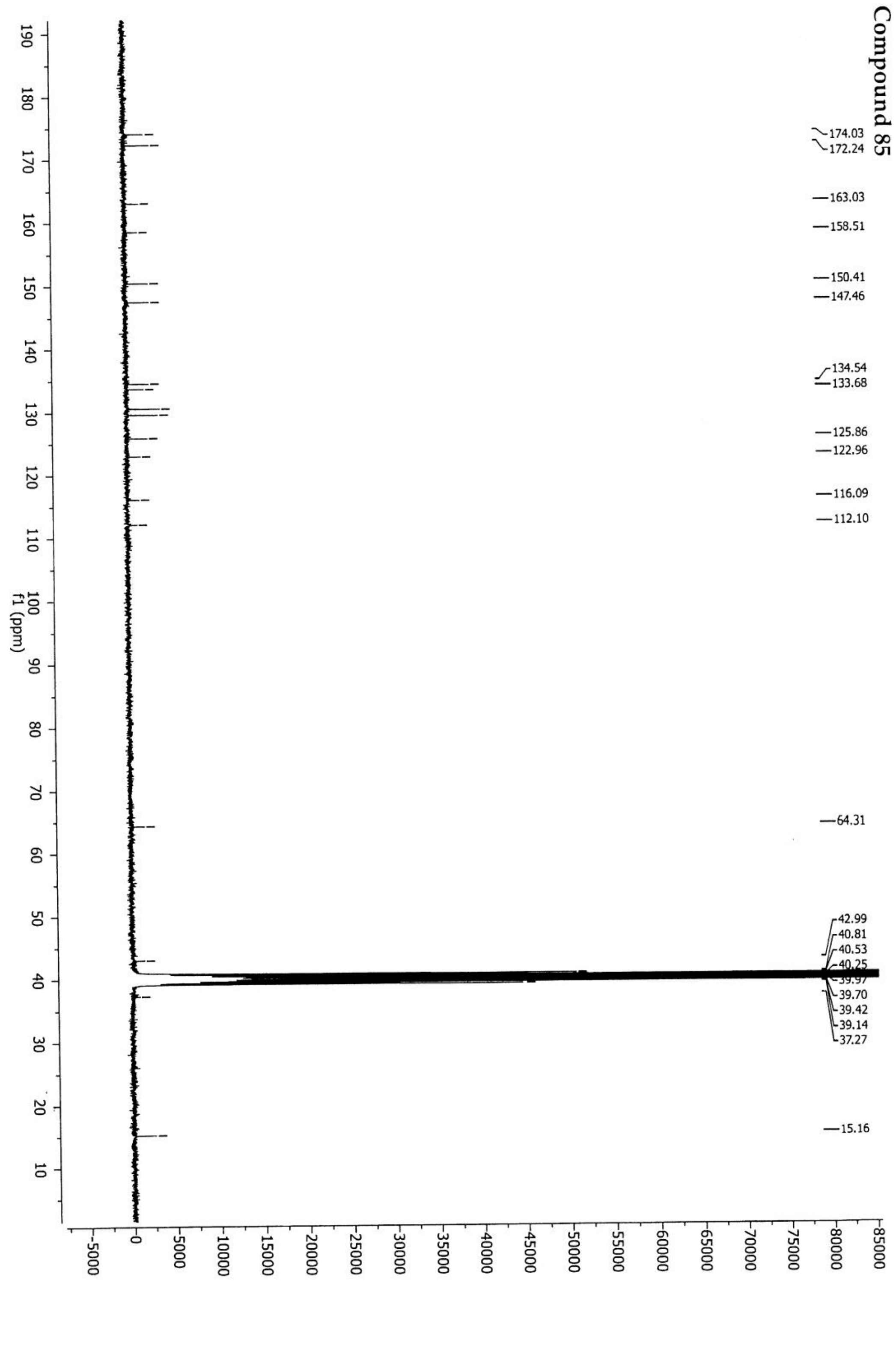

Fig. S78. ^{13}C NMR spectrum for compound **85**

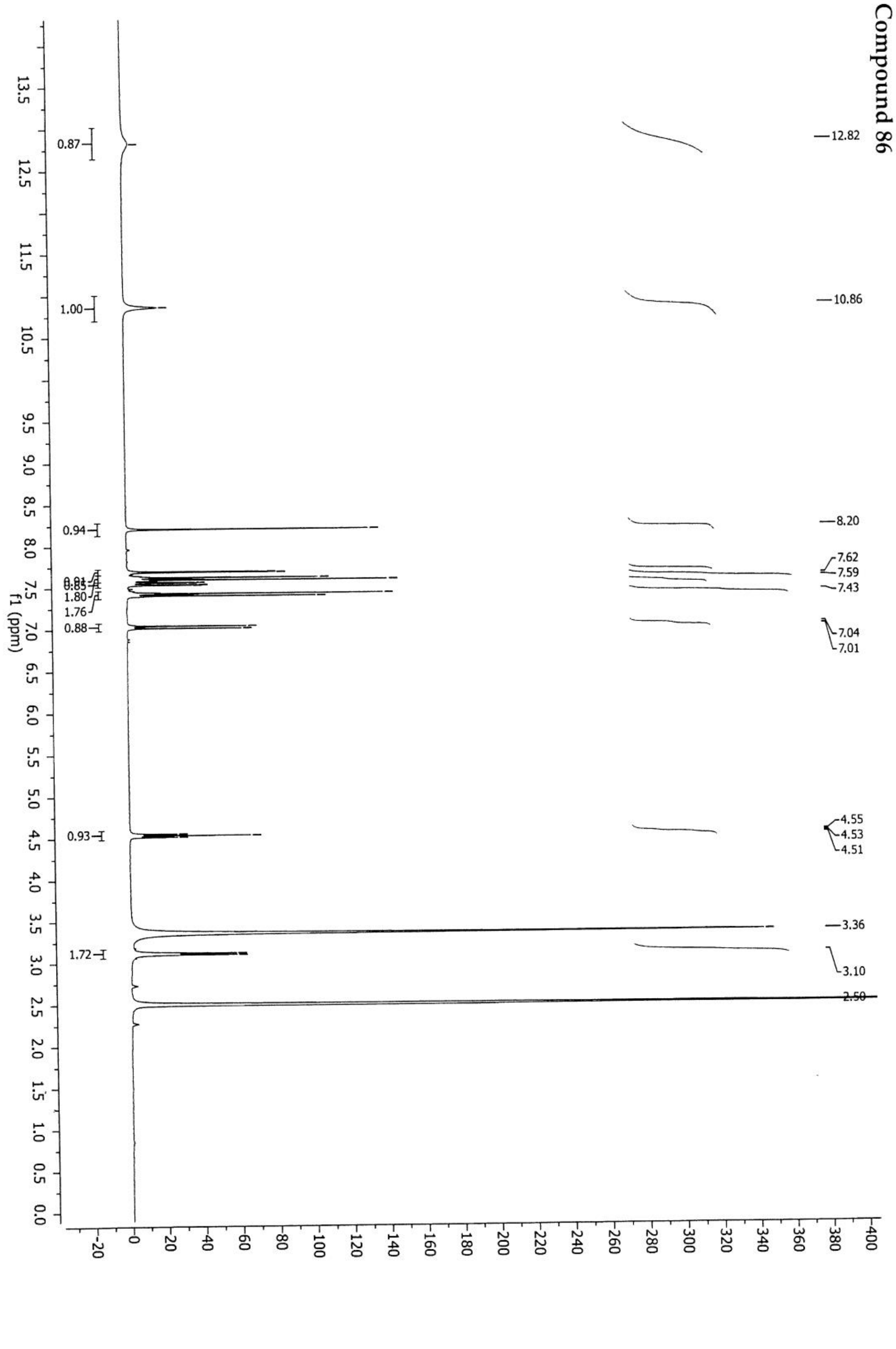

Fig. S79. ^1H NMR spectrum for compound **86**

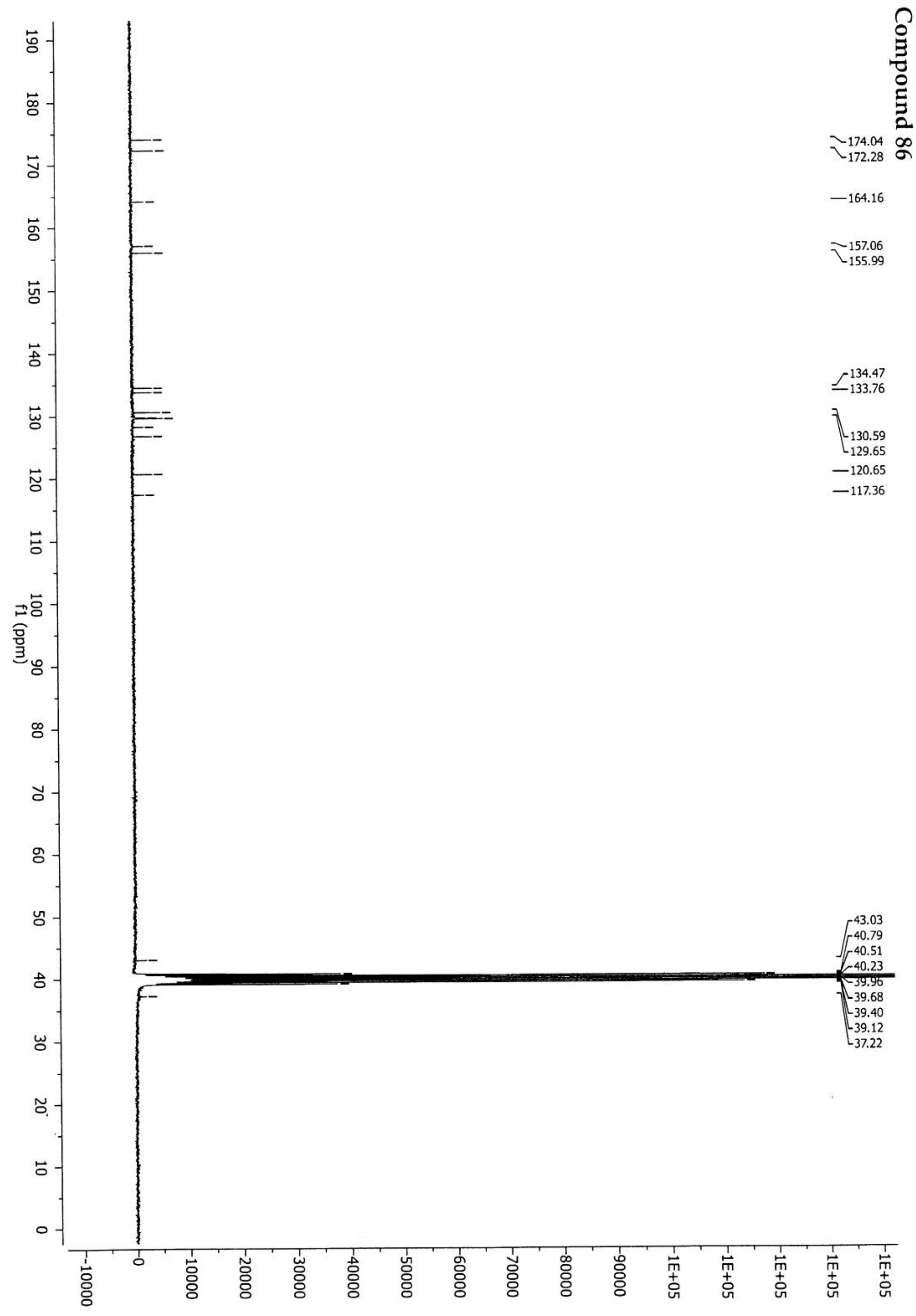

Fig. S80. ^{13}C NMR spectrum for compound **86**

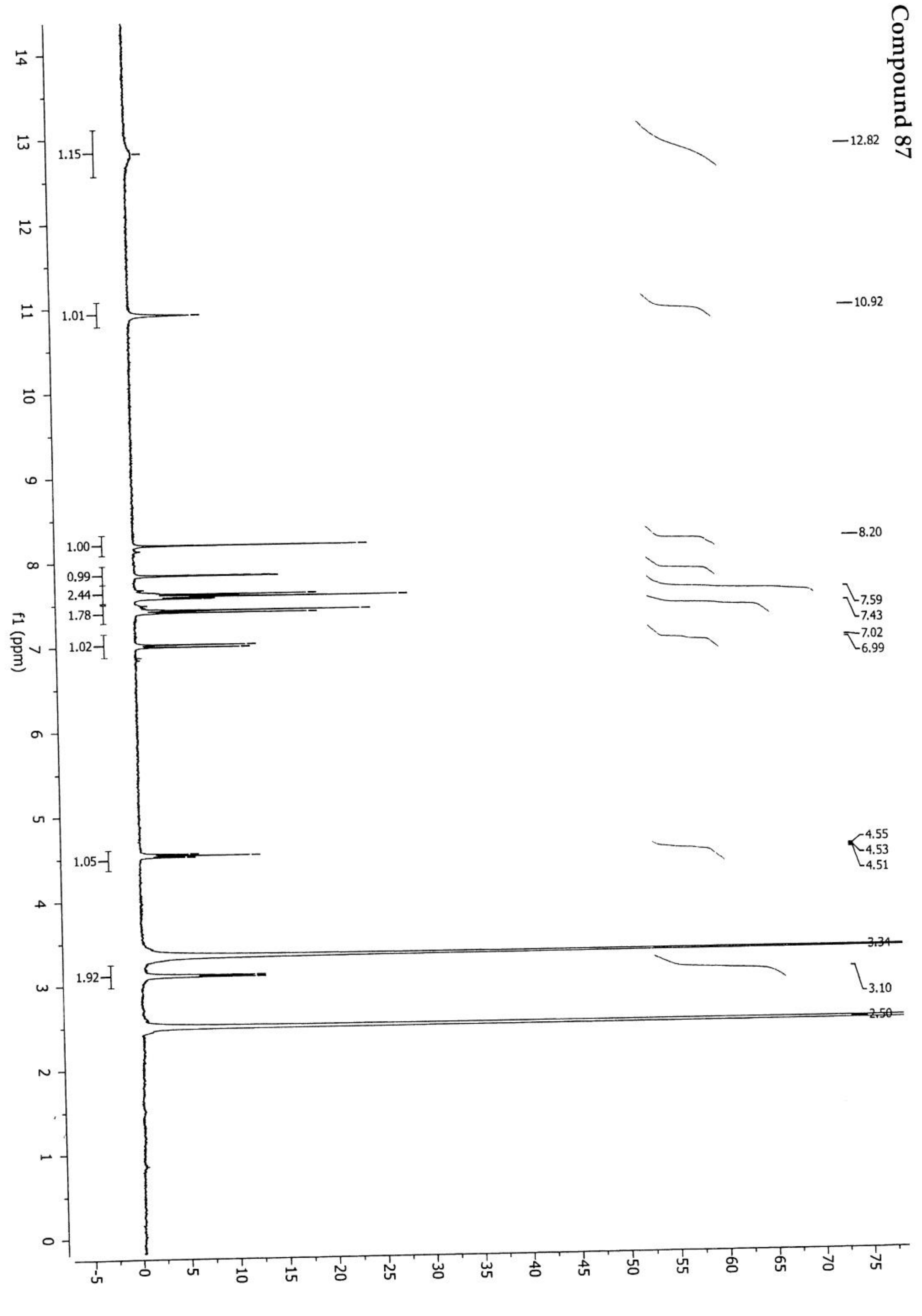

Fig. S81. ^1H NMR spectrum for compound **87**

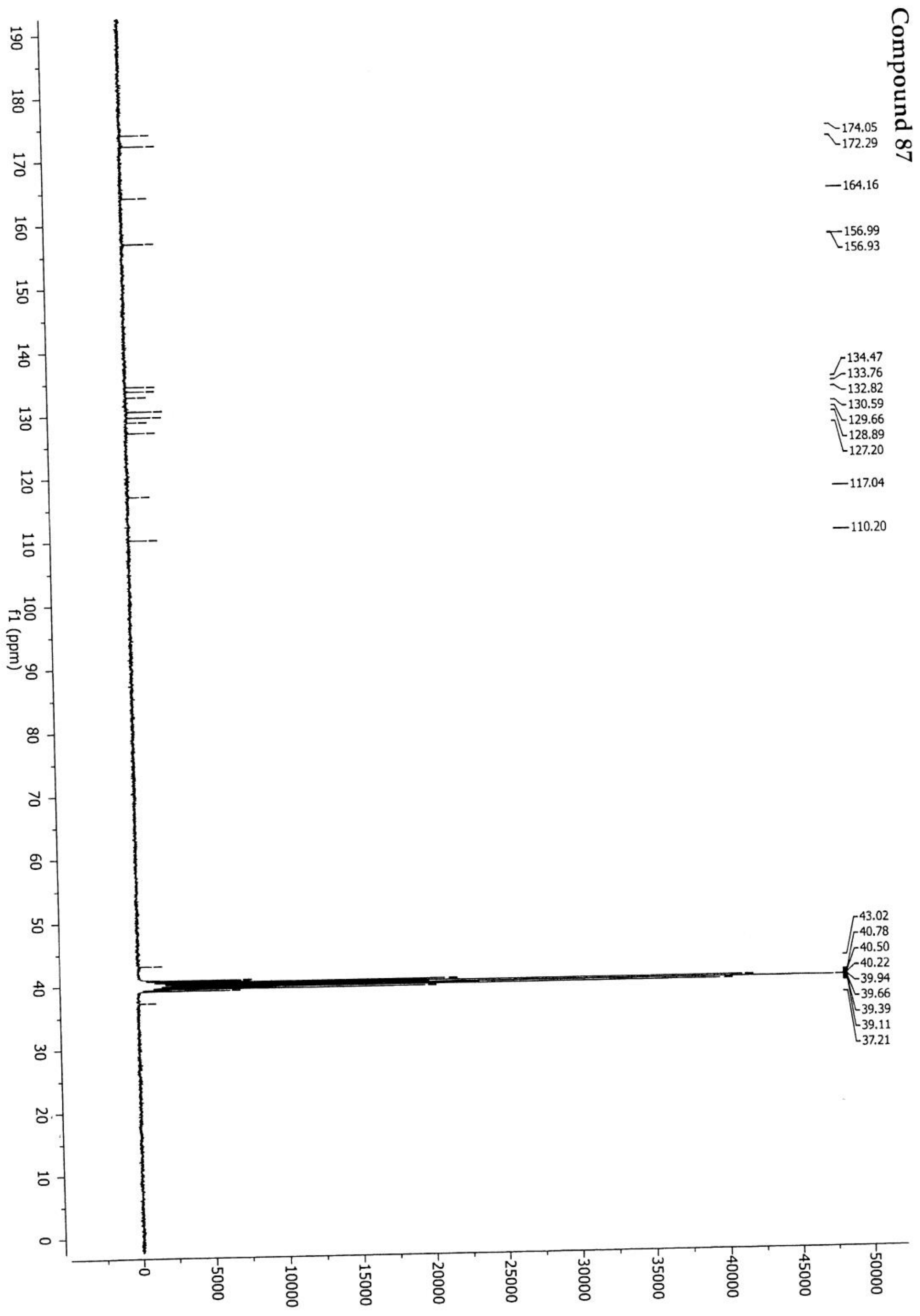

Fig. S82. ^{13}C NMR spectrum for compound **87**

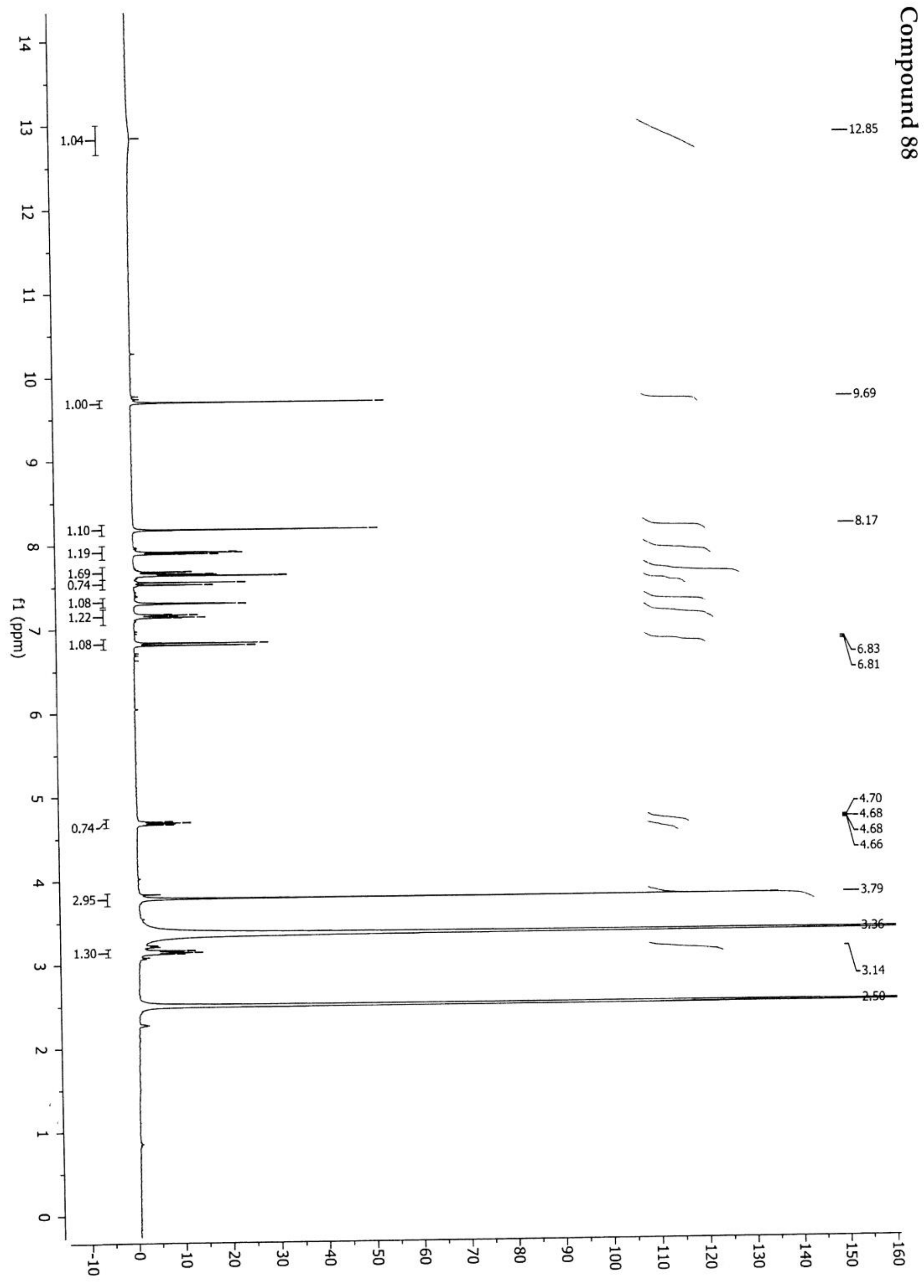

Fig. S83. ¹H NMR spectrum for compound **88**

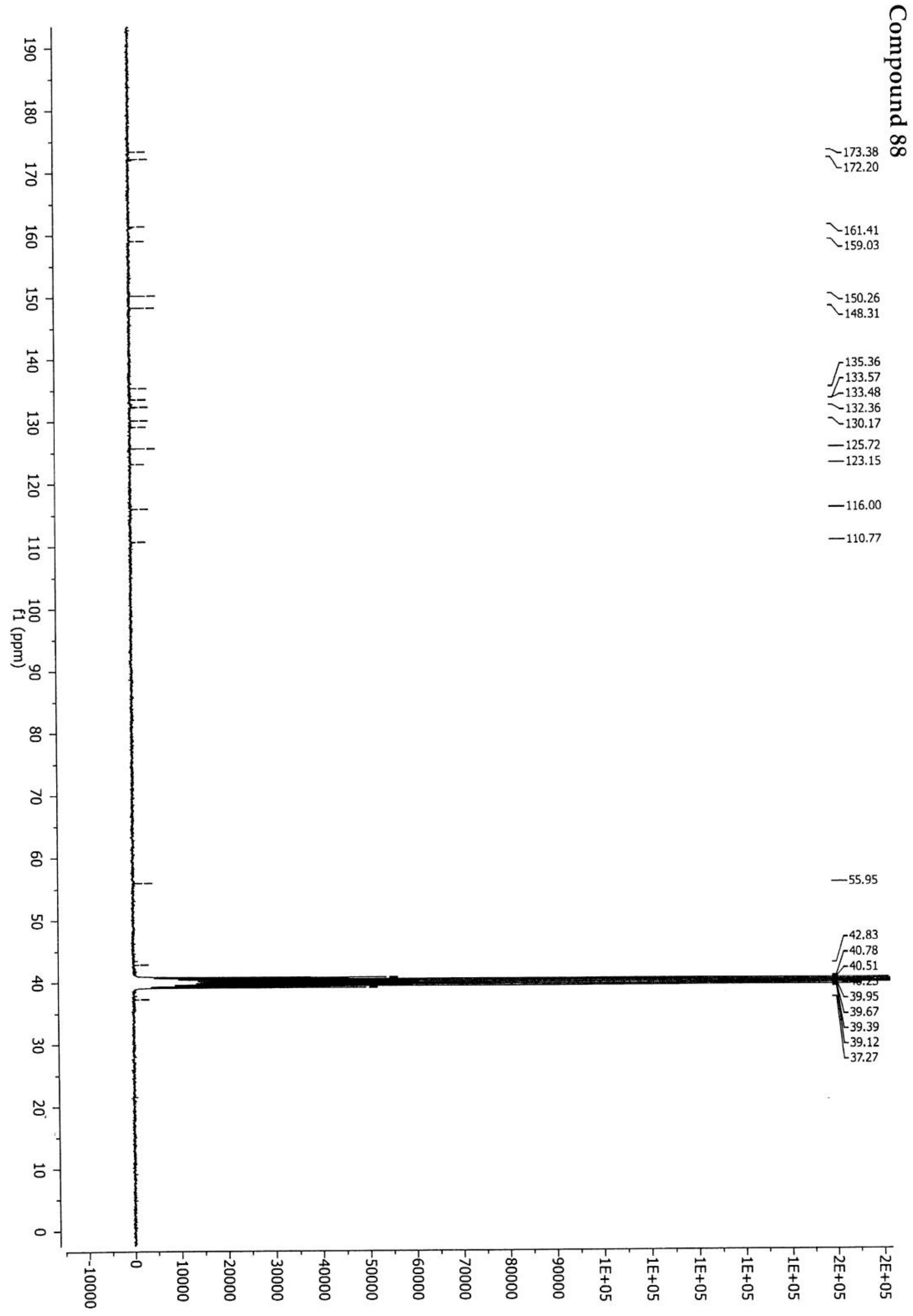

Fig. S84. ^{13}C NMR spectrum for compound **88**

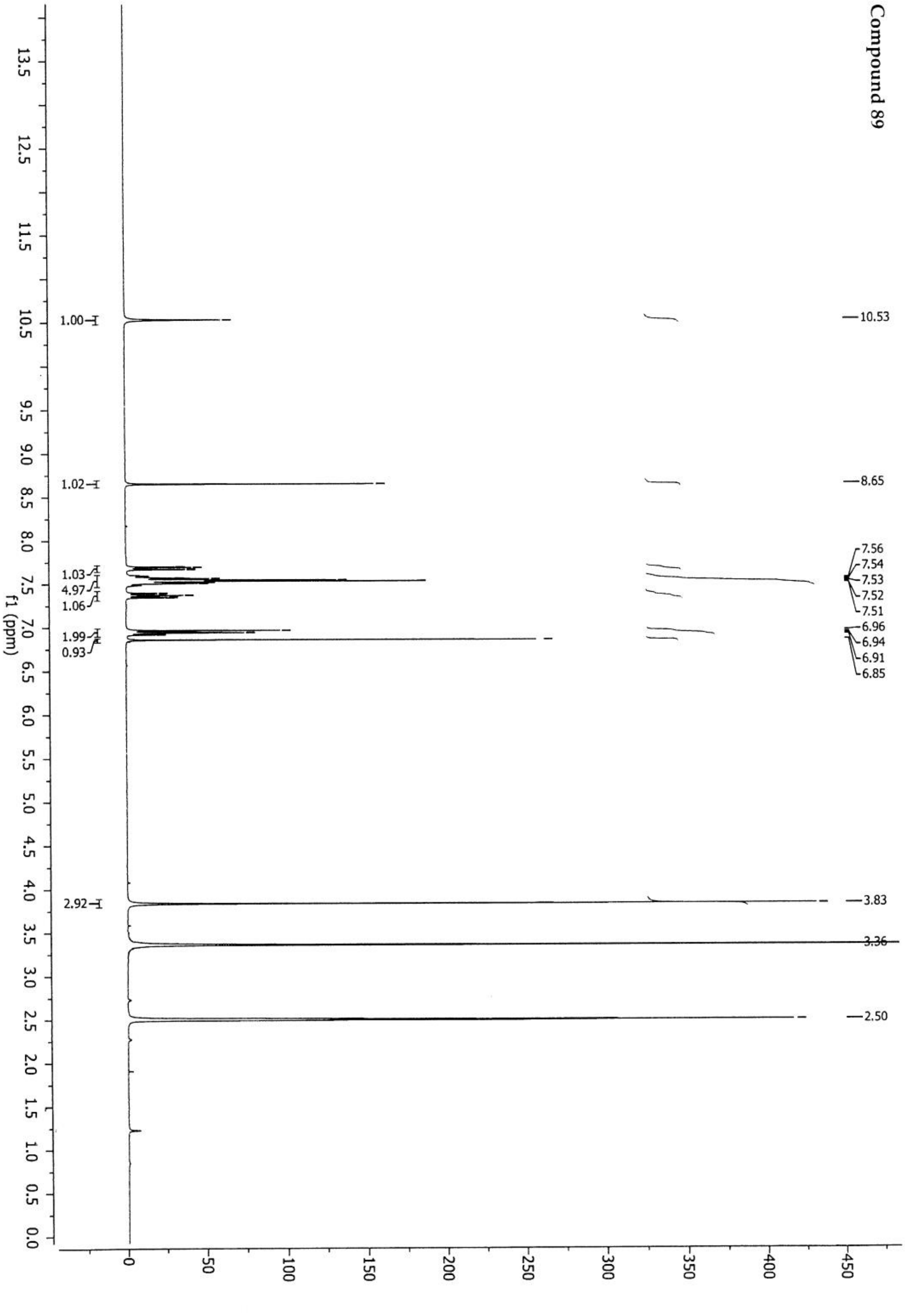

Fig. S85. ¹H NMR spectrum for compound **89**

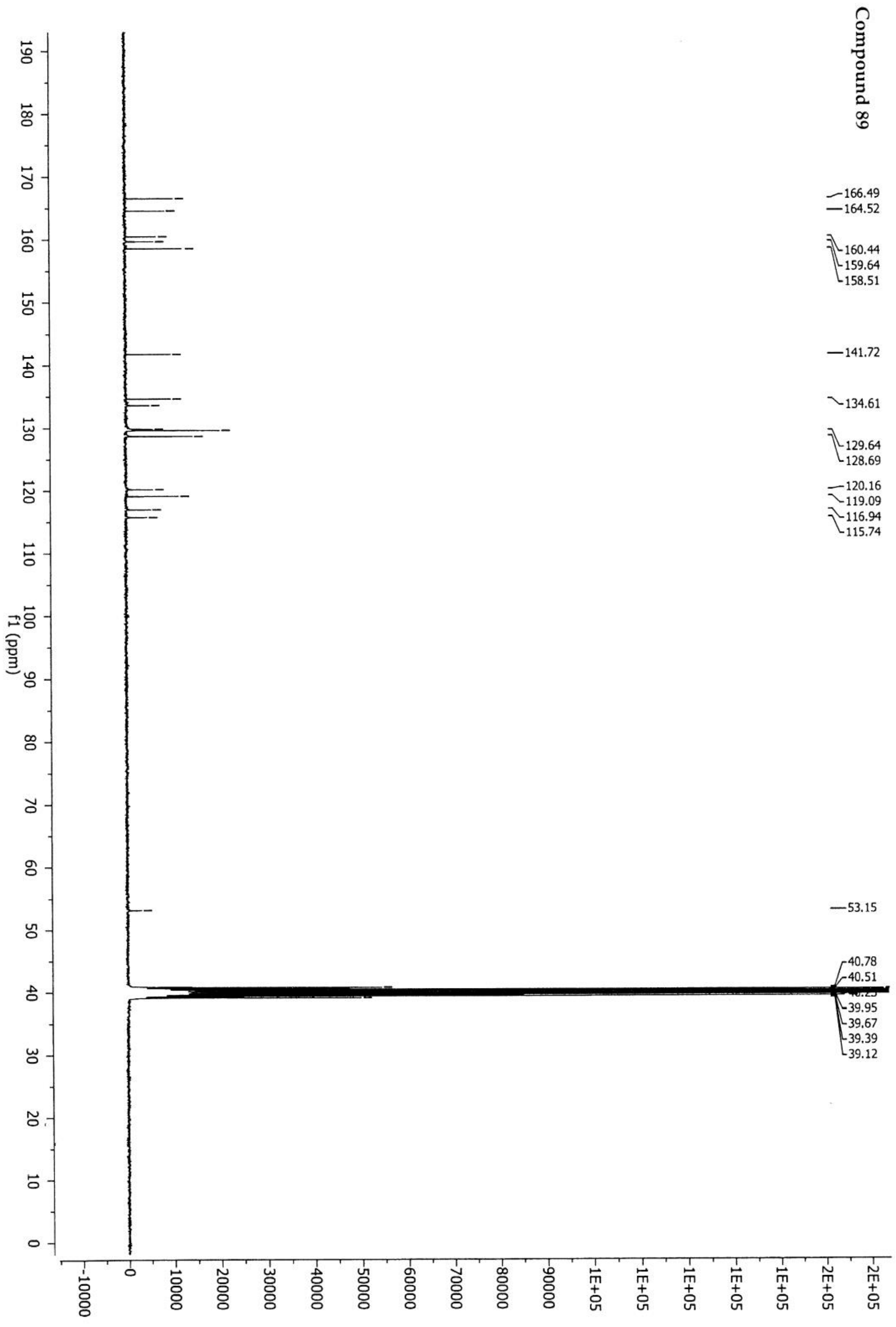

Fig. S86. ^{13}C NMR spectrum for compound **89**

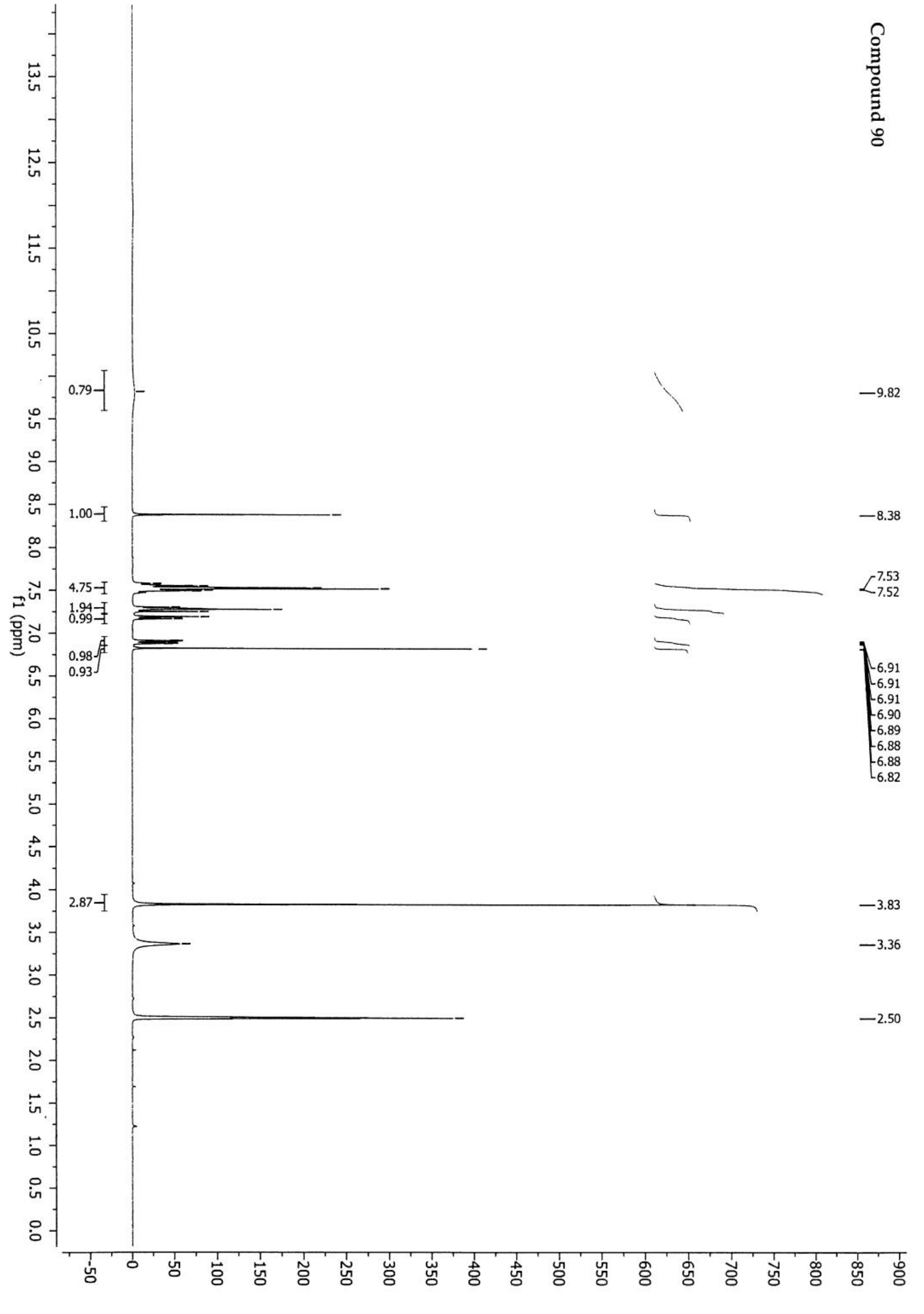

Fig. S87. ^{1}H NMR spectrum for compound **90**

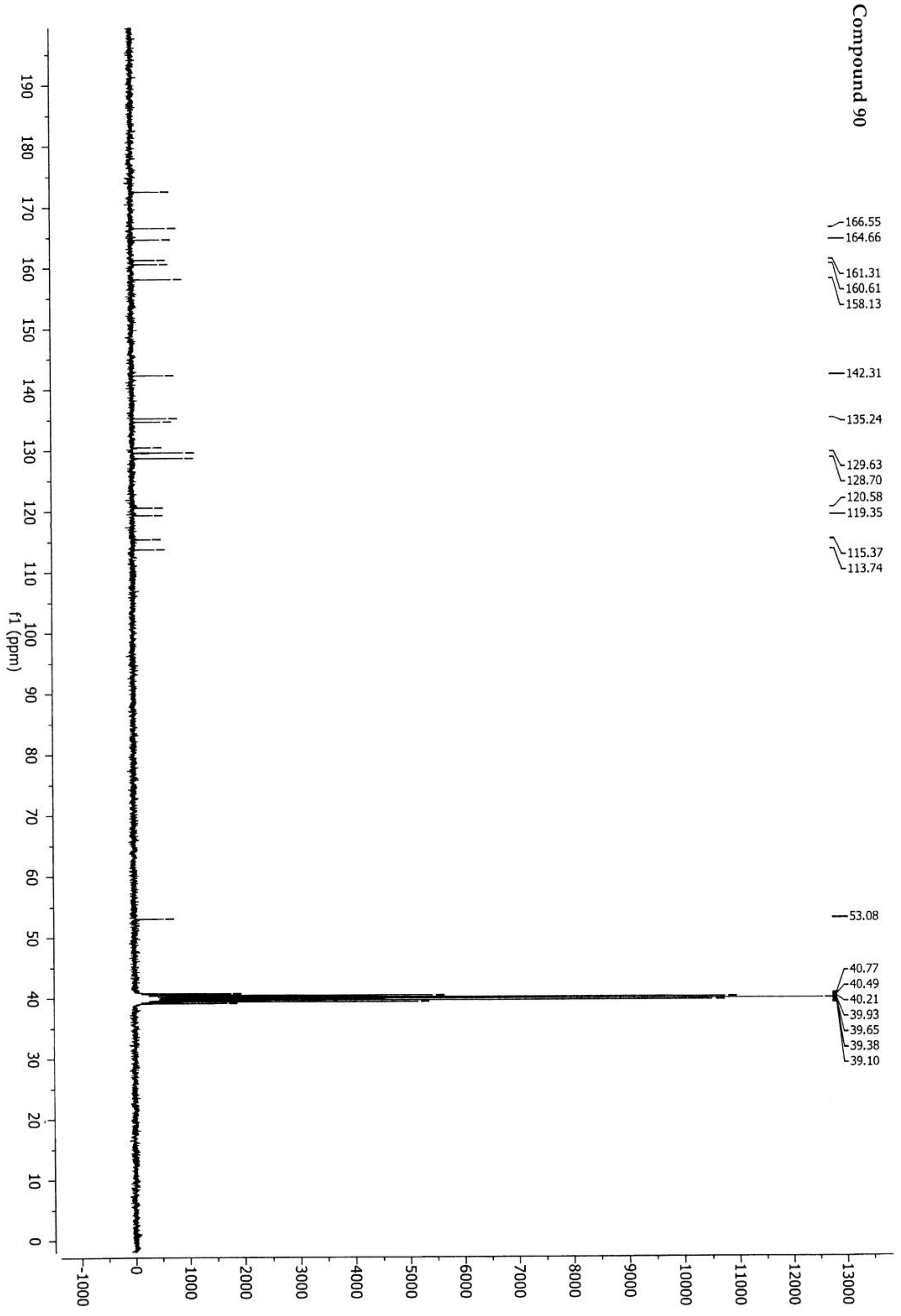

Fig. S88. ^{13}C NMR spectrum for compound **90**

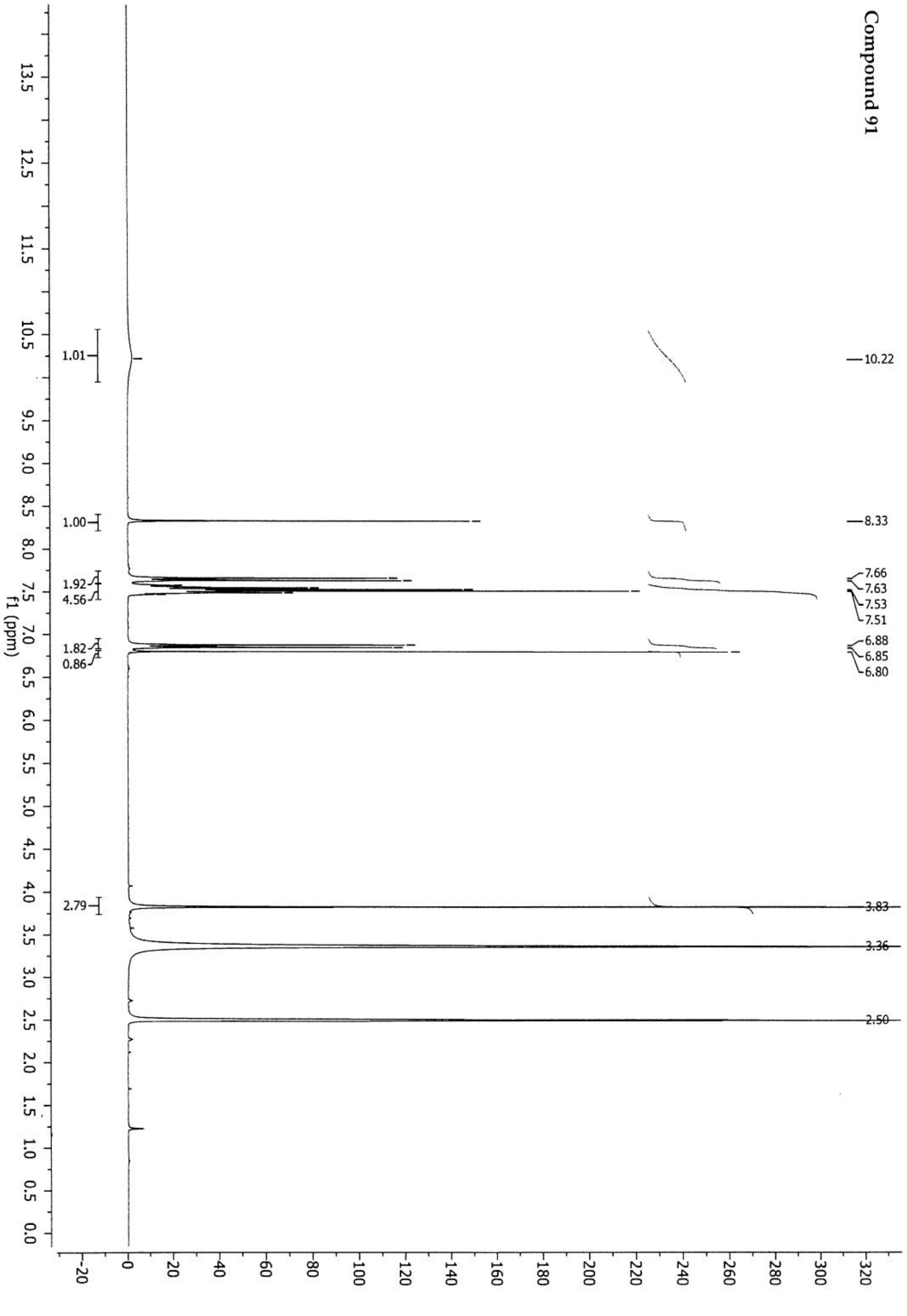

Fig. S89. ¹H NMR spectrum for compound **91**

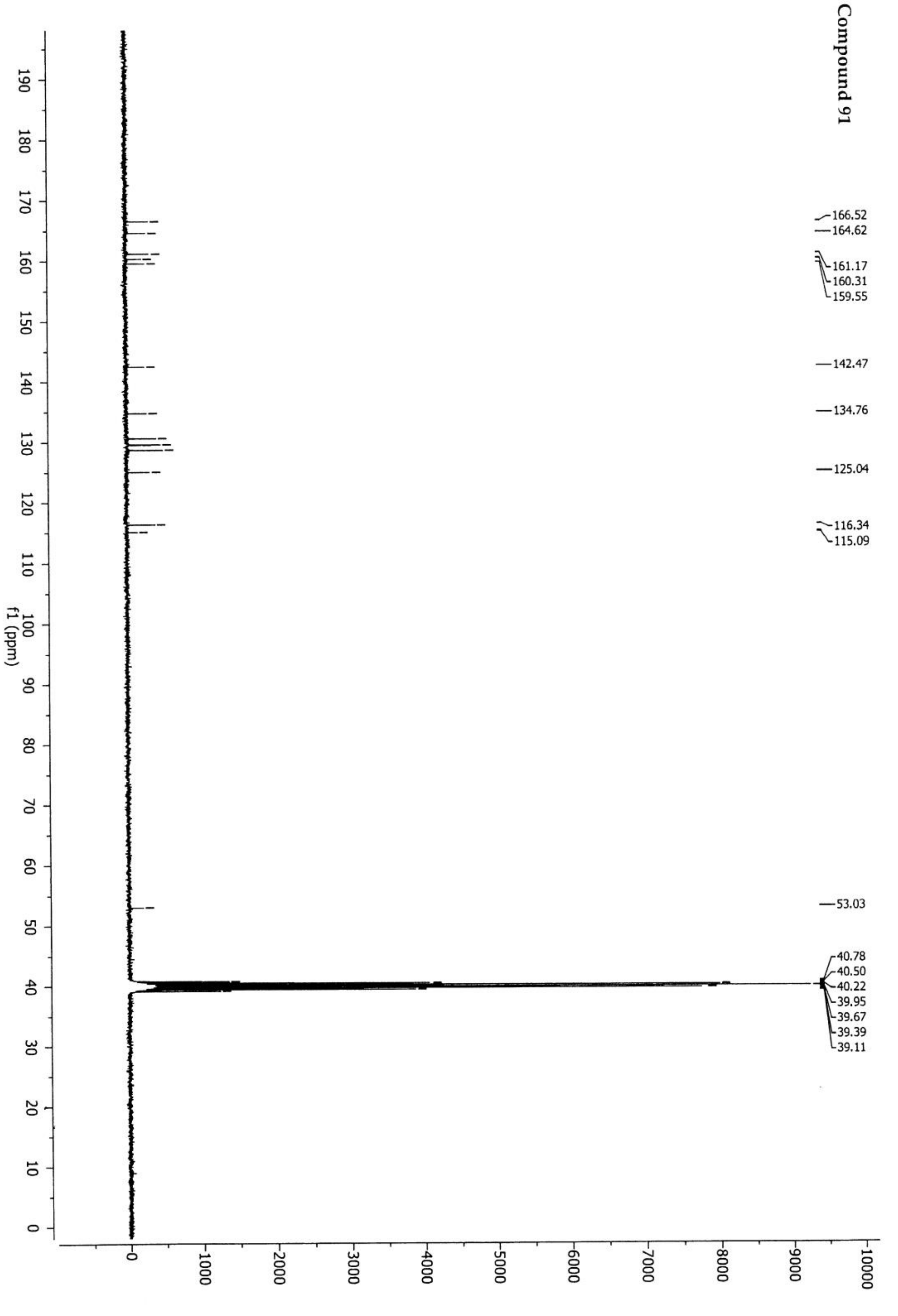

Fig. S90. ^{13}C NMR spectrum for compound **91**

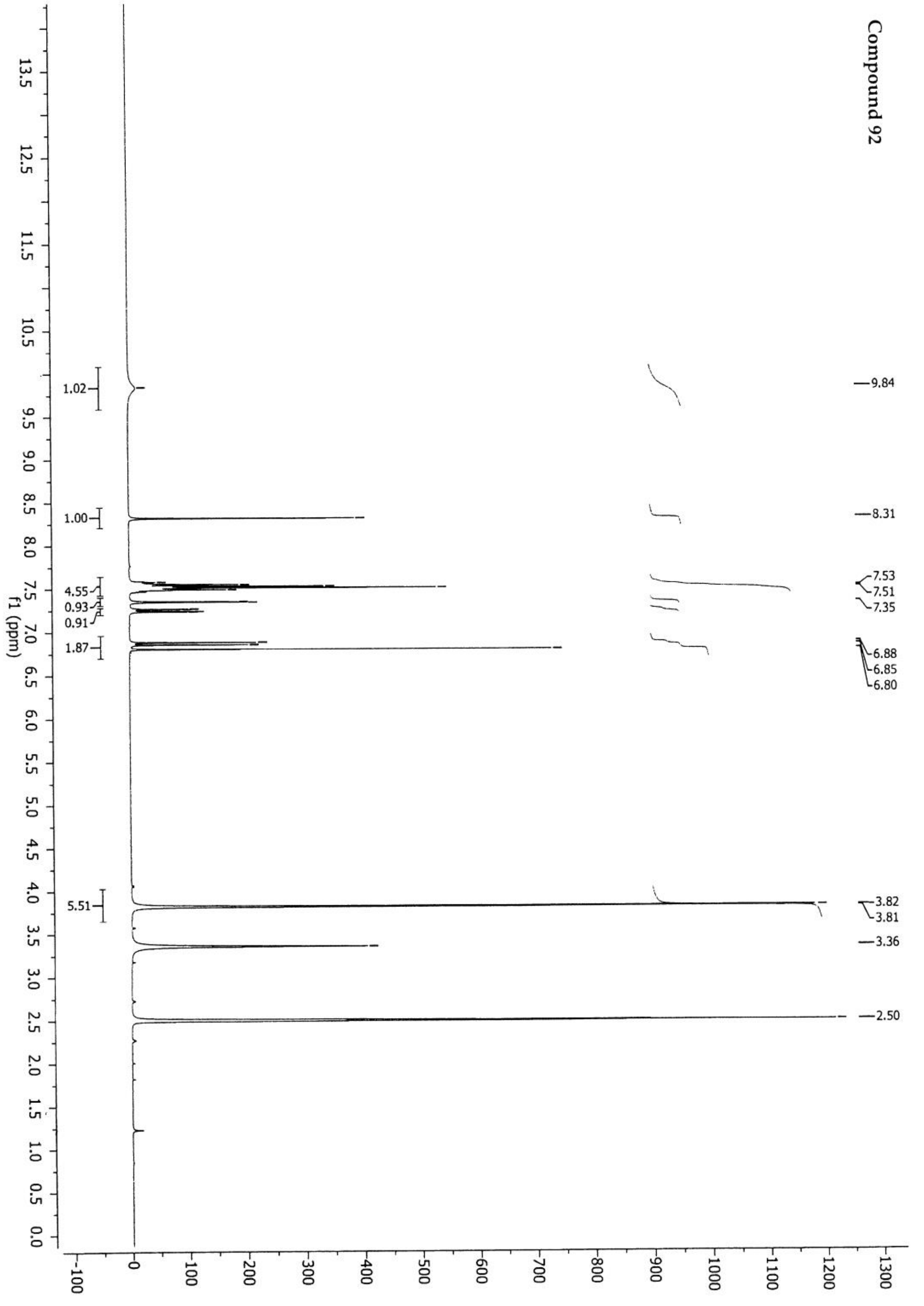

Fig. S91. ^{1}H NMR spectrum for compound **92**

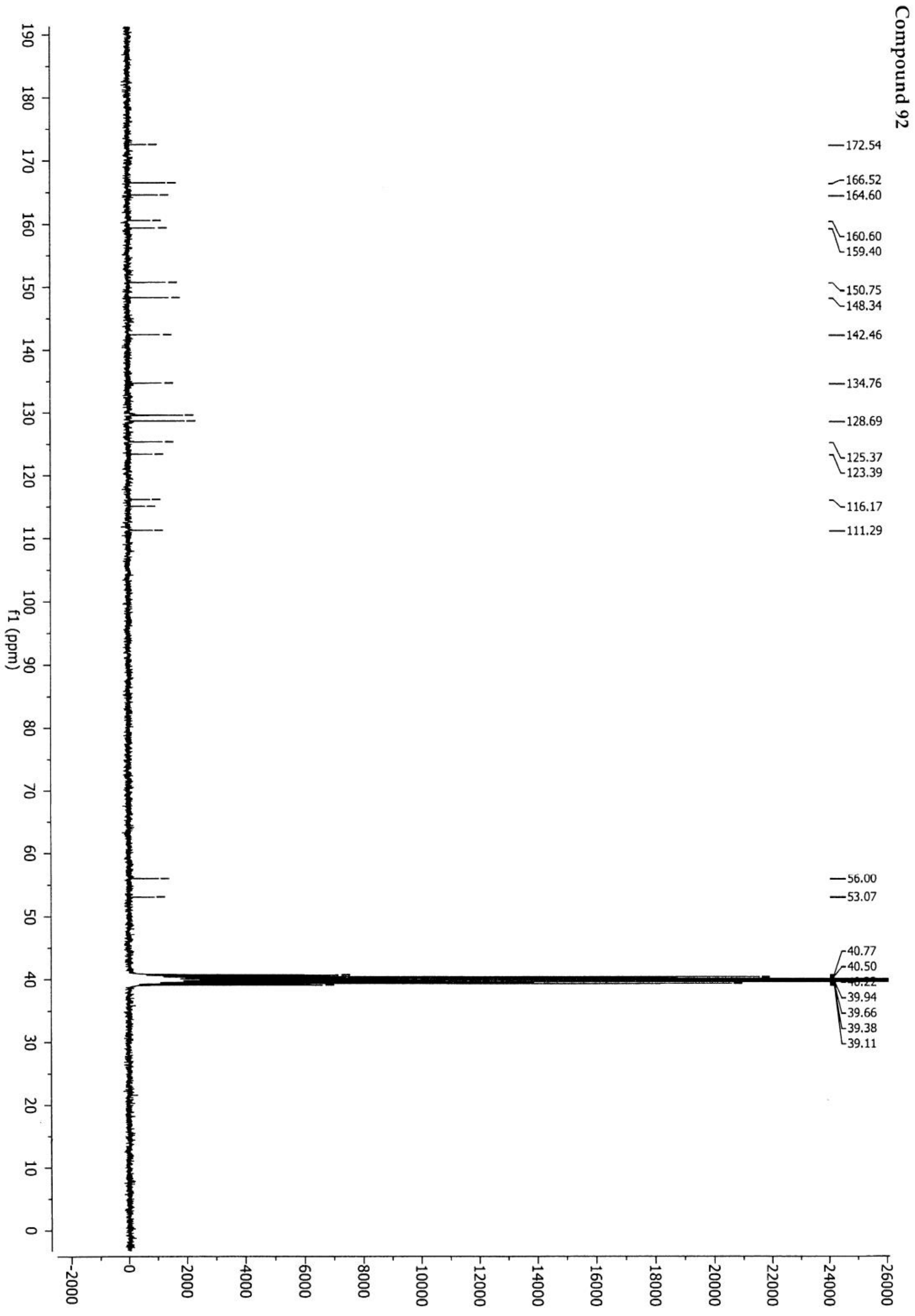

Fig. S92. ^{13}C NMR spectrum for compound **92**

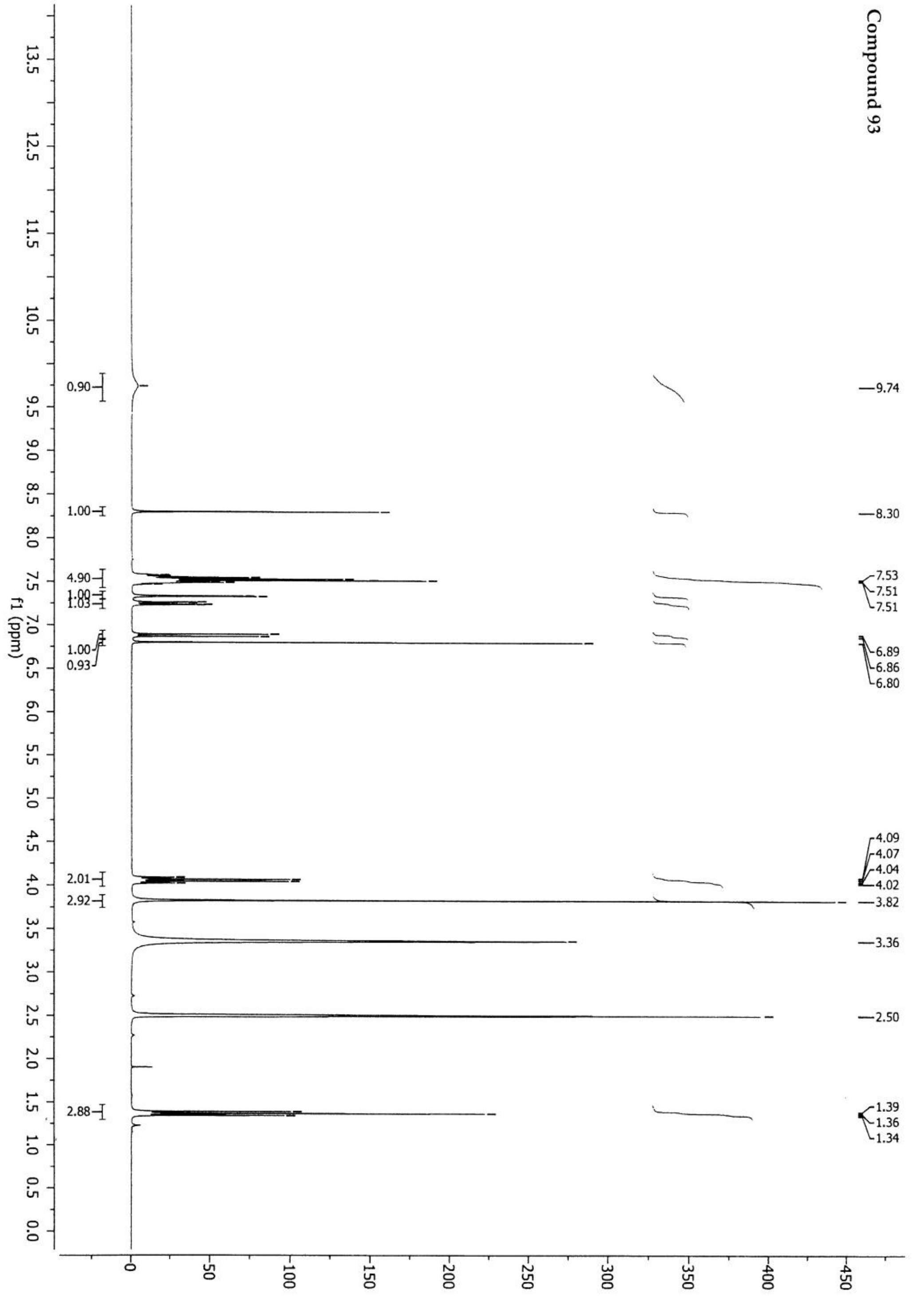

Fig. S93. ^{1}H NMR spectrum for compound **93**

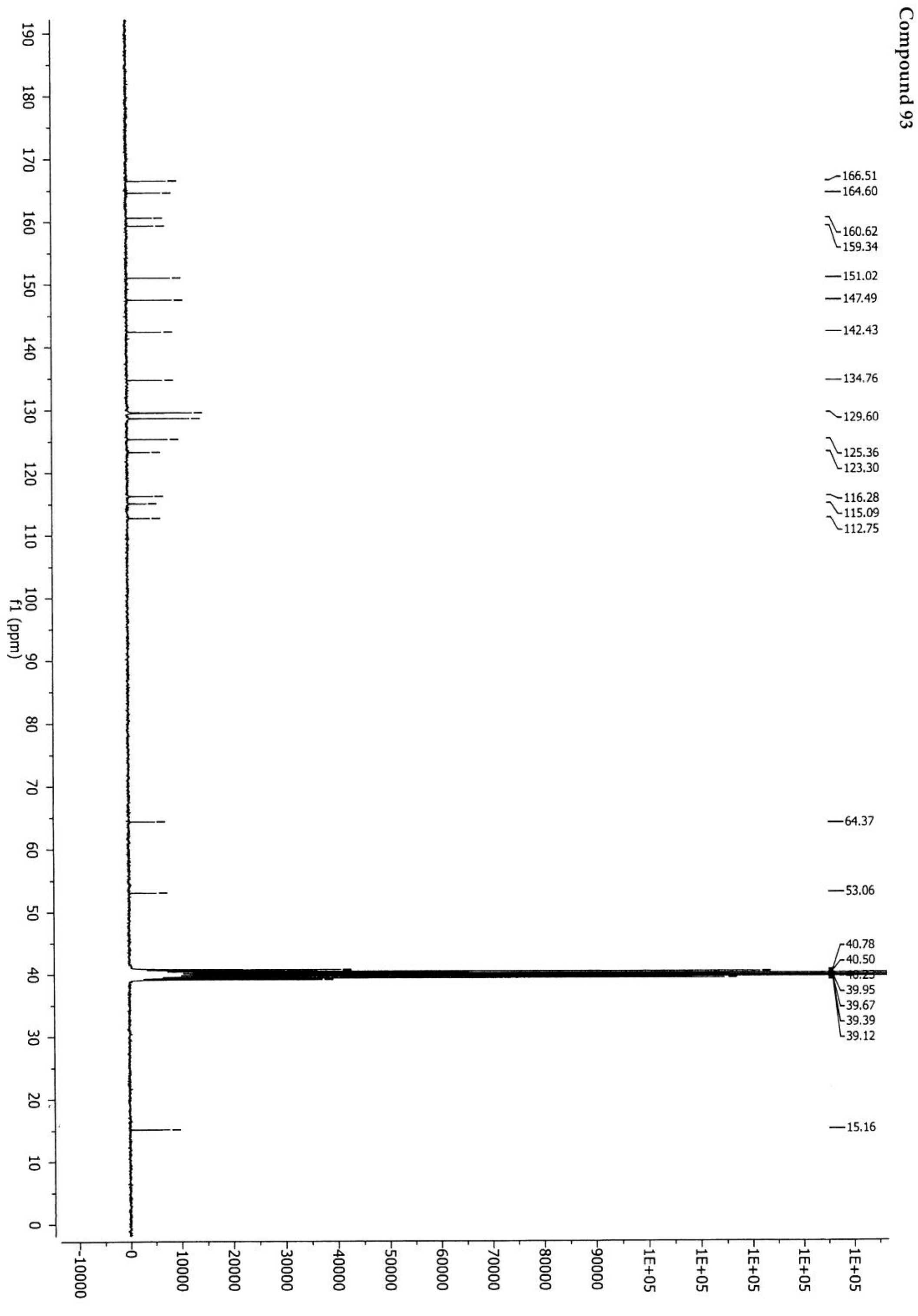

Fig. S94. ^{13}C NMR spectrum for compound **93**

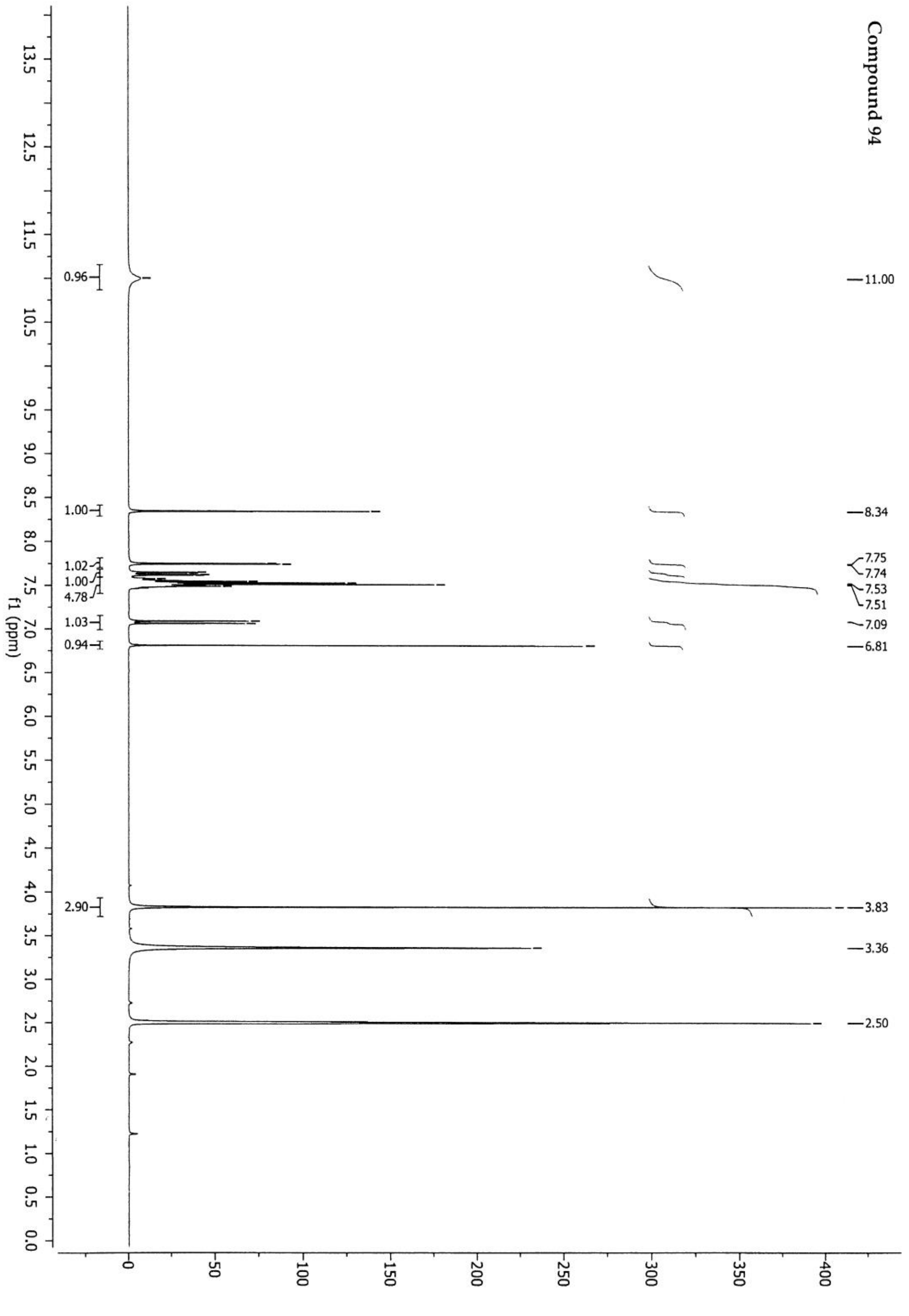

Fig. S95. ^{1}H NMR spectrum for compound **94**

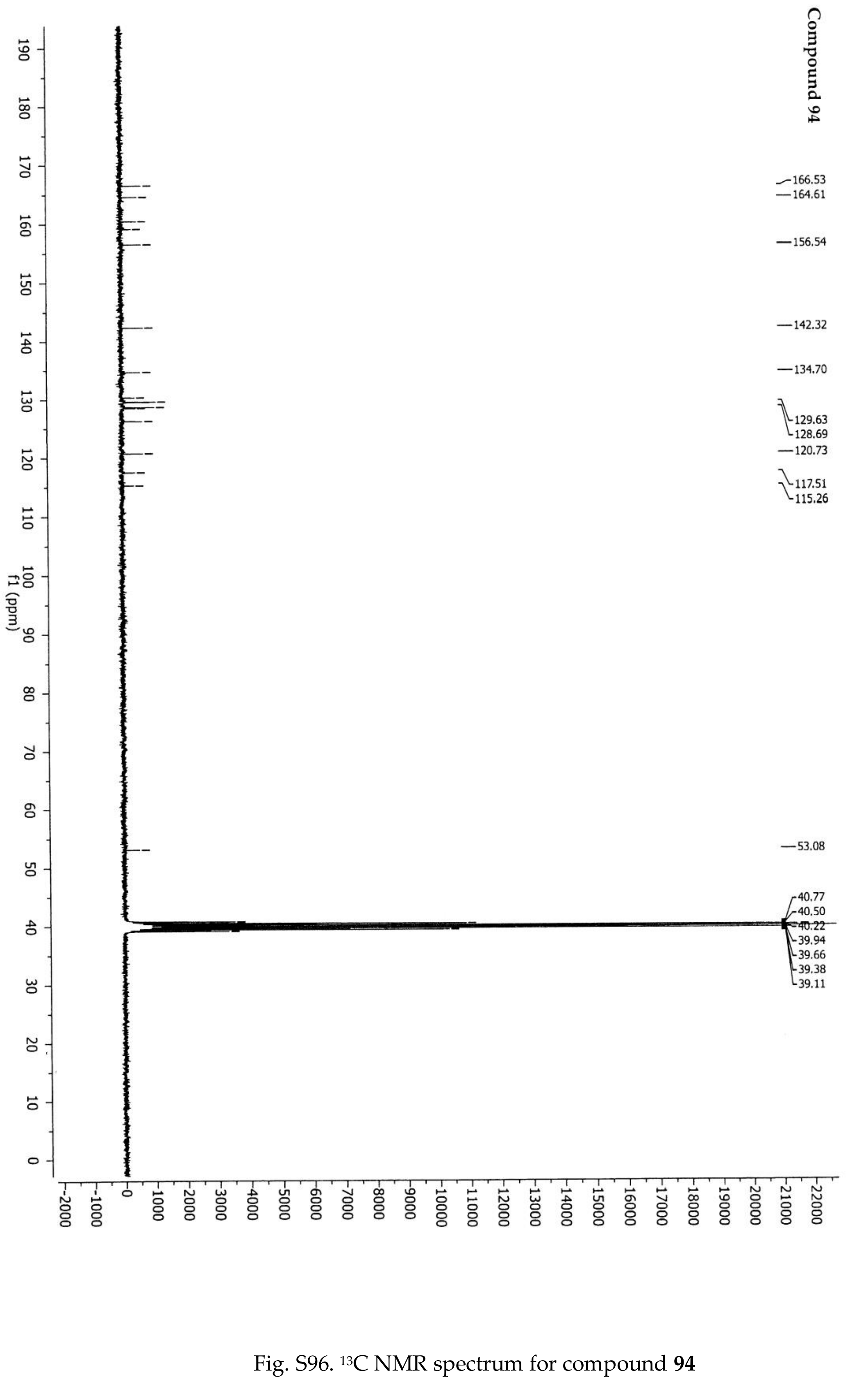

Fig. S96. ^{13}C NMR spectrum for compound **94**

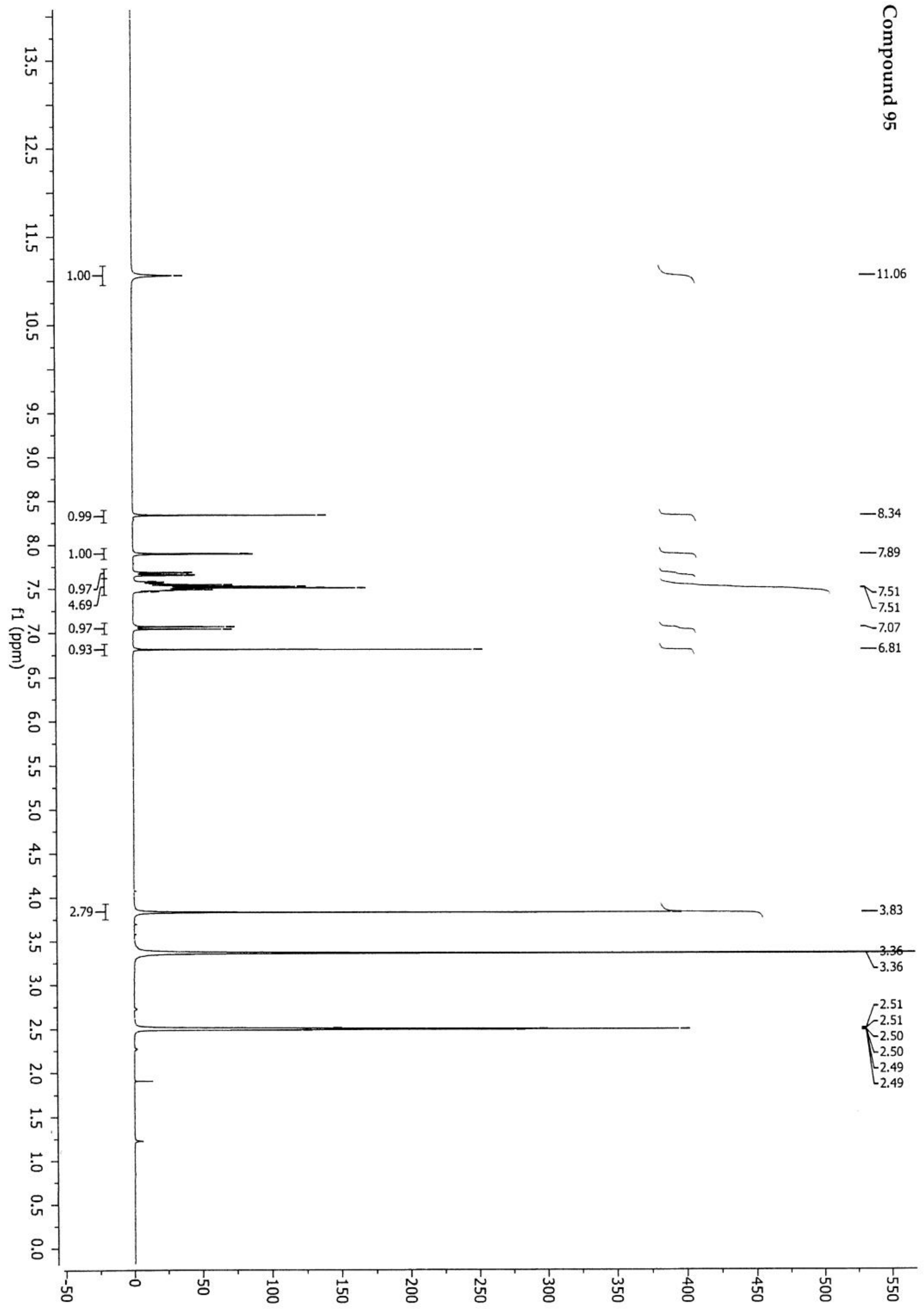

Fig. S97. ¹H NMR spectrum for compound **95**

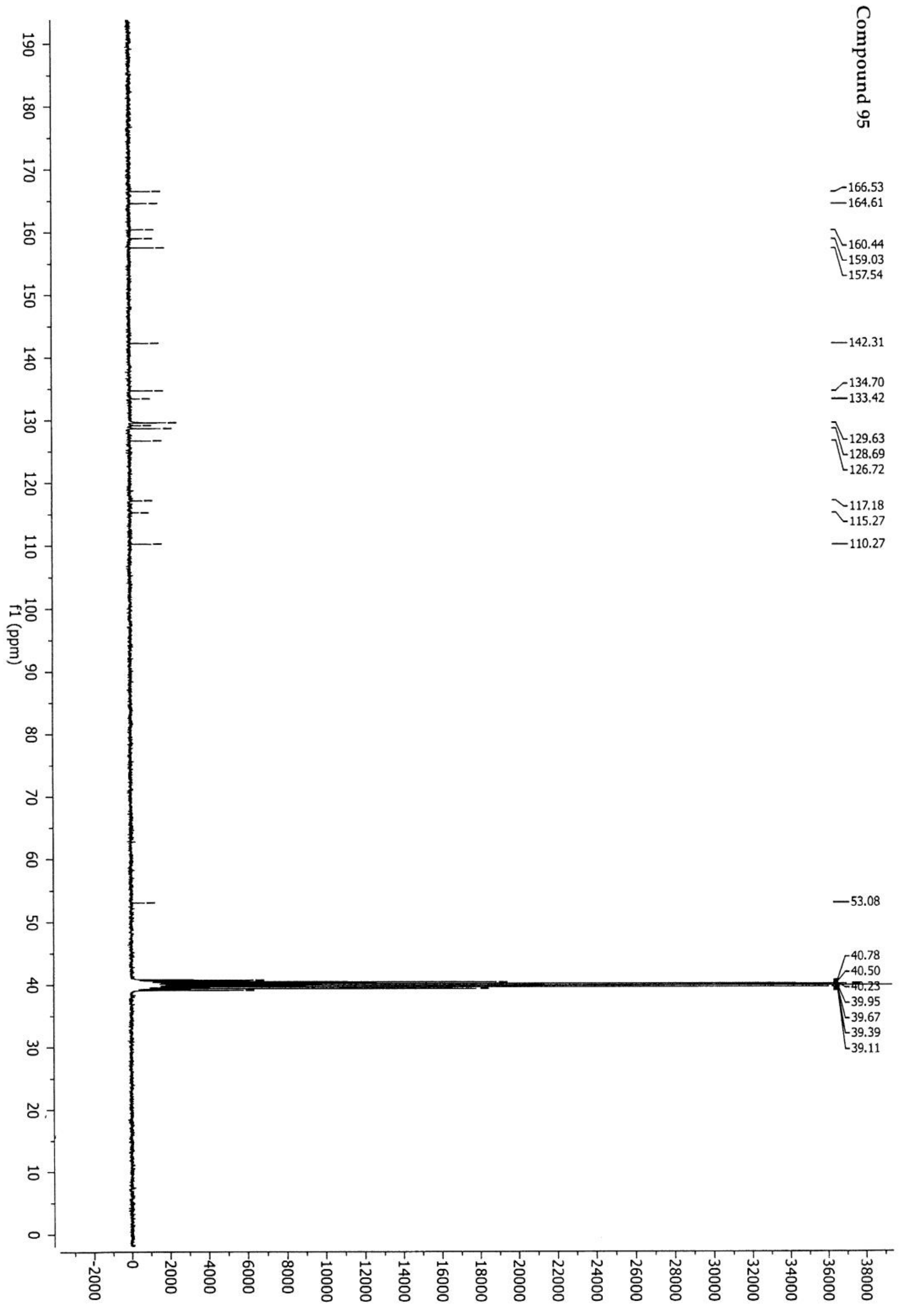

Fig. S98. ^{13}C NMR spectrum for compound **95**

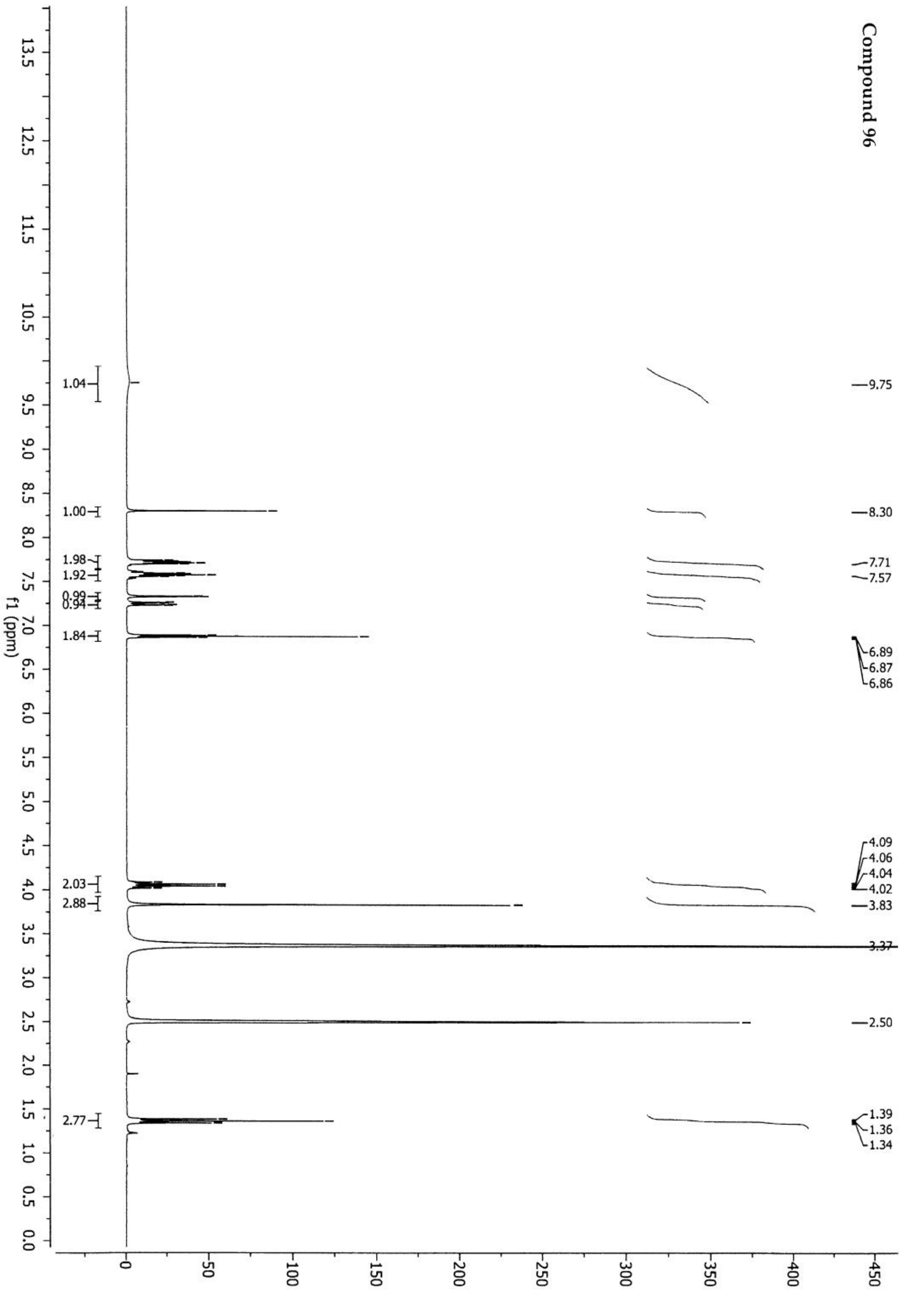

Fig. S99. ^{1}H NMR spectrum for compound **96**

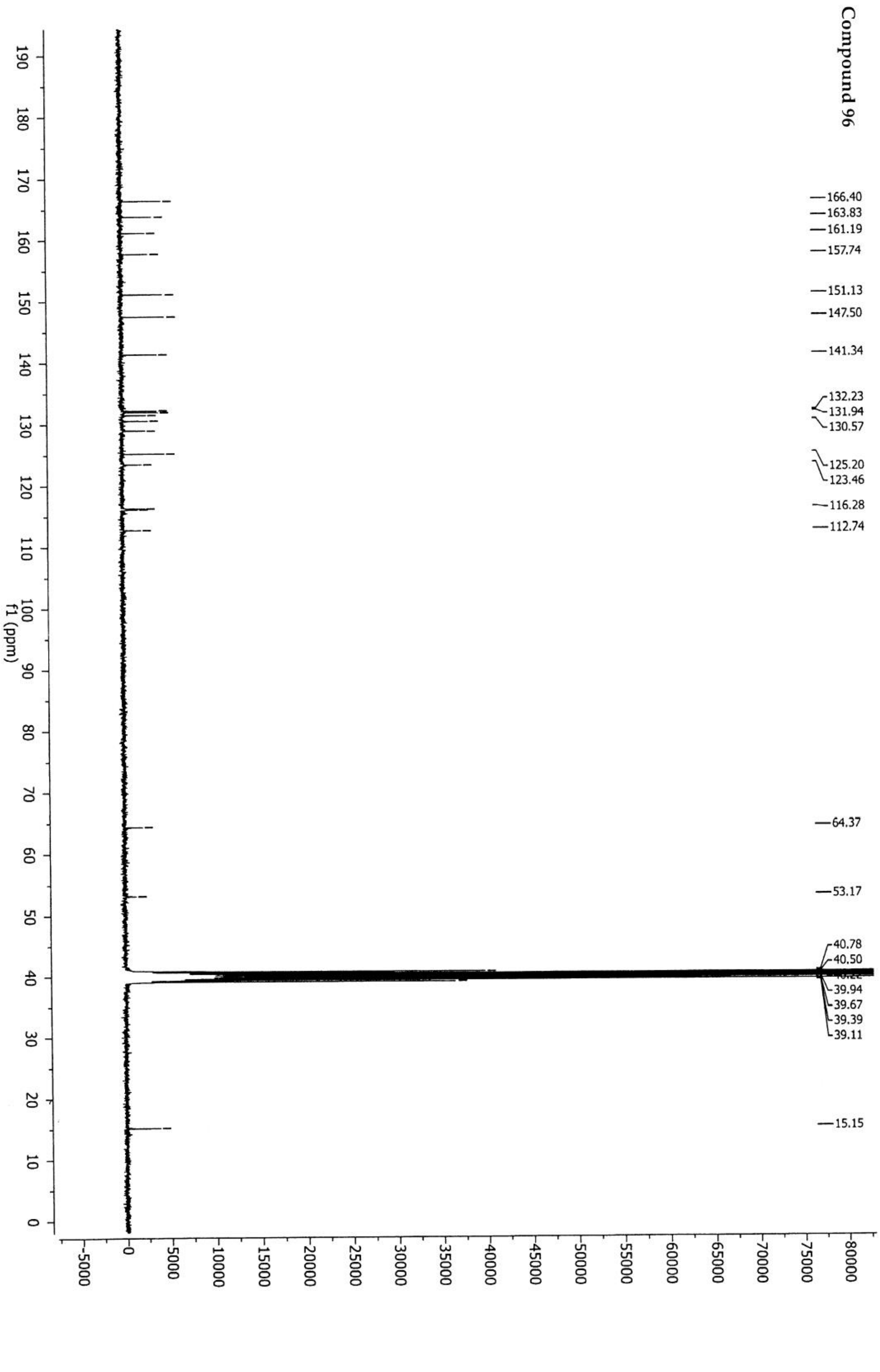

Fig. S100. ^{13}C NMR spectrum for compound **96**

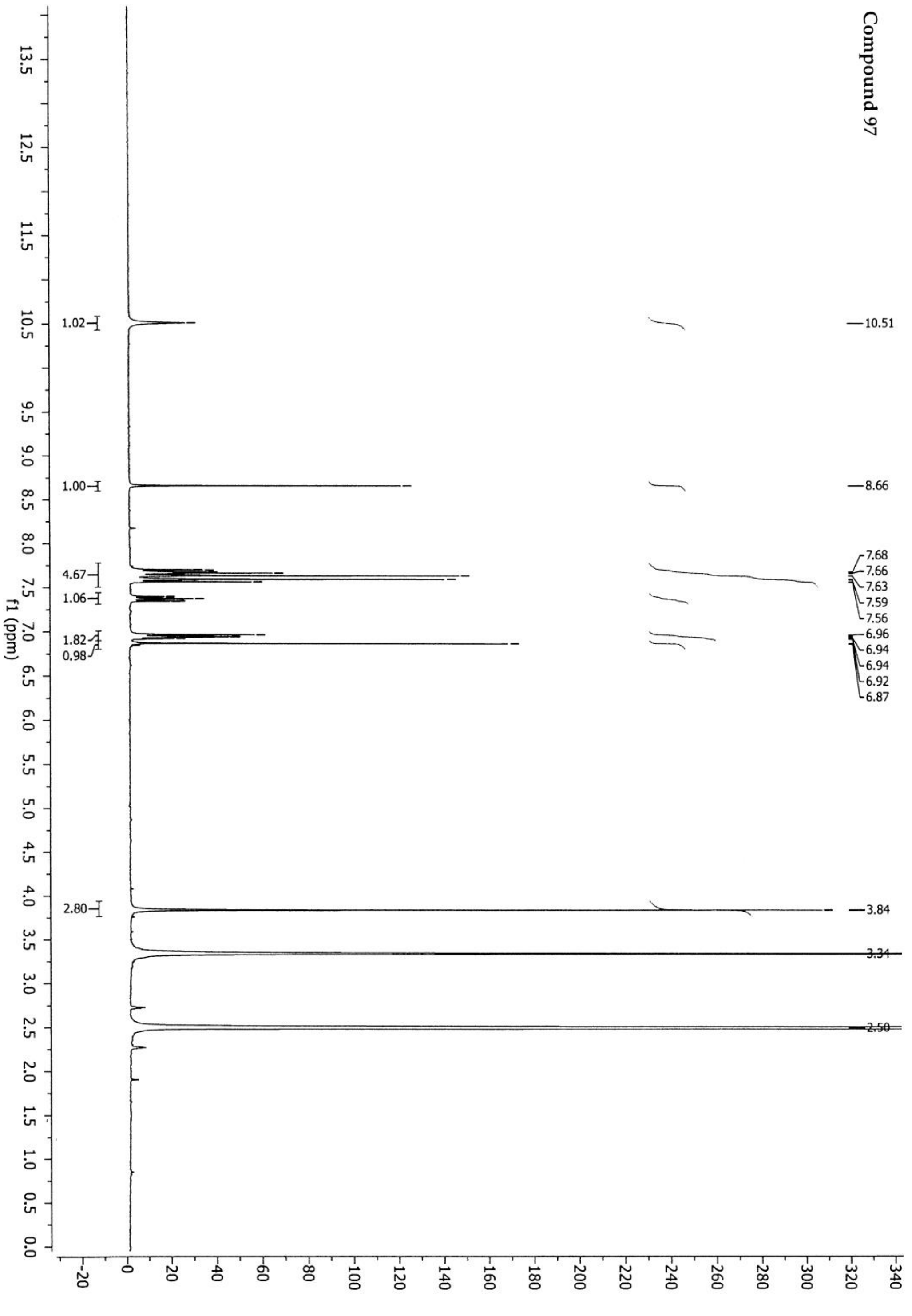

Fig. S101. ¹H NMR spectrum for compound **97**

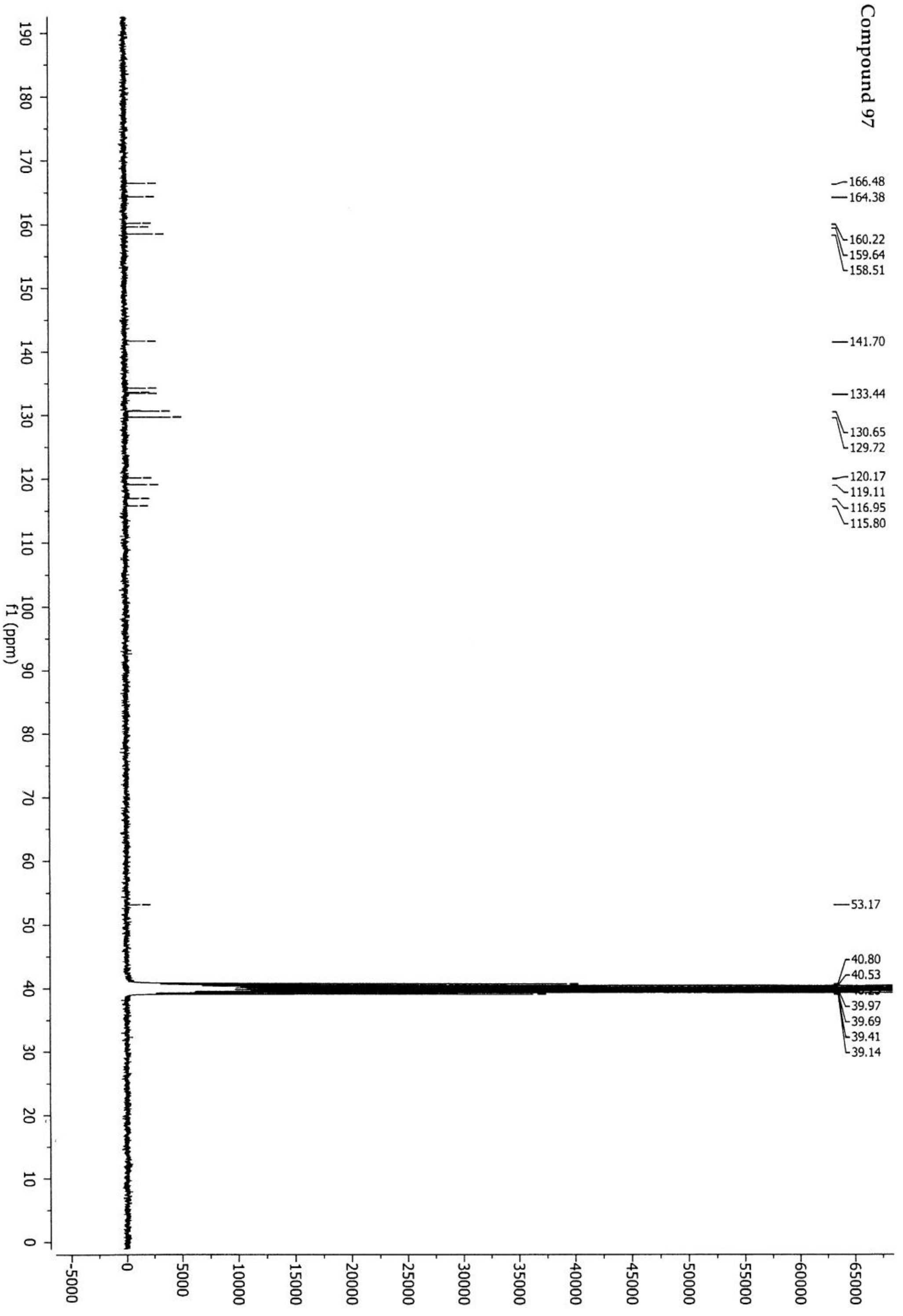

Fig. S102. ^{13}C NMR spectrum for compound **97**

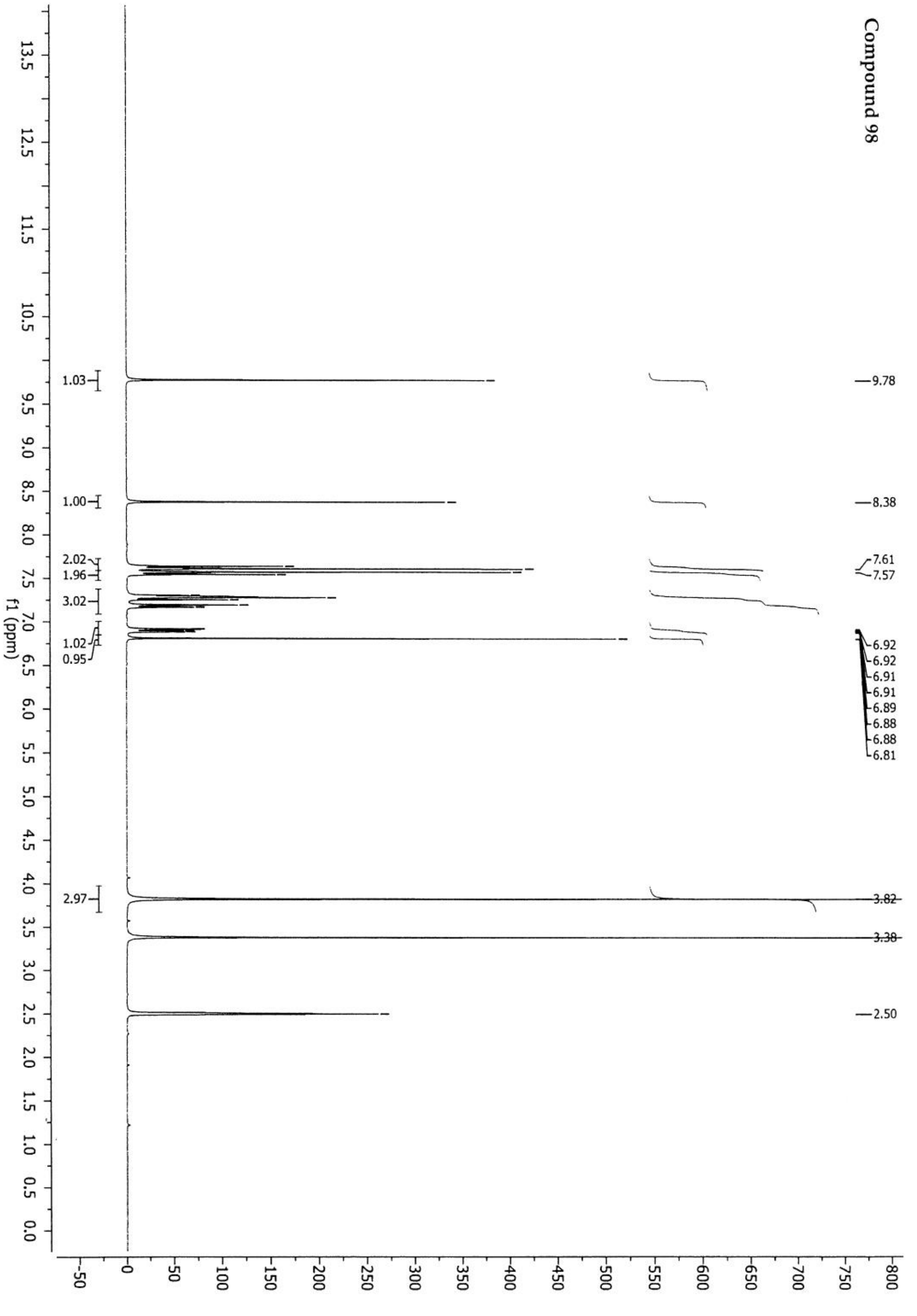

Fig. S103. ^{1}H NMR spectrum for compound **98**

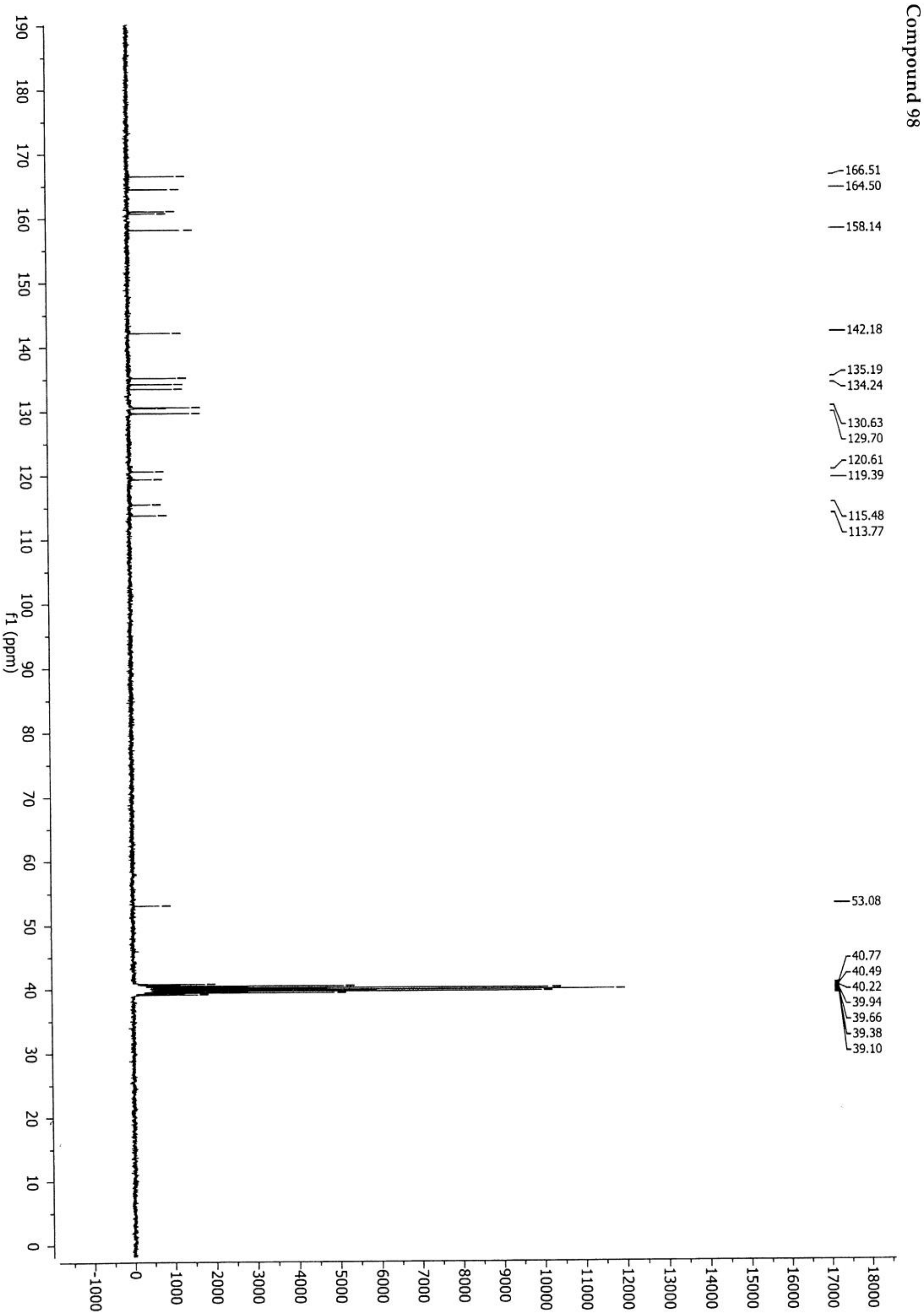

Fig. S104. ^{13}C NMR spectrum for compound **98**

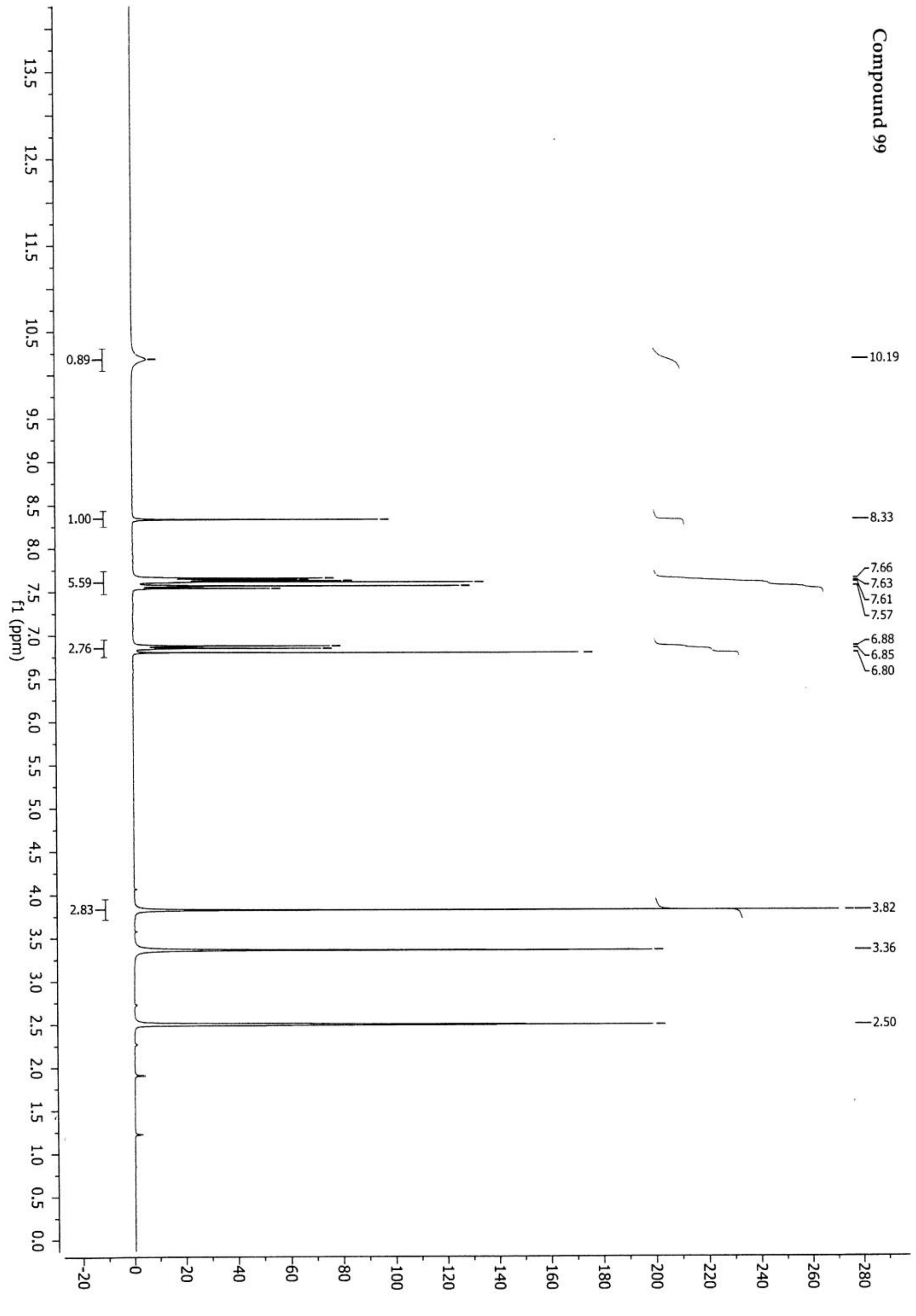

Fig. S105. ^{1}H NMR spectrum for compound **99**

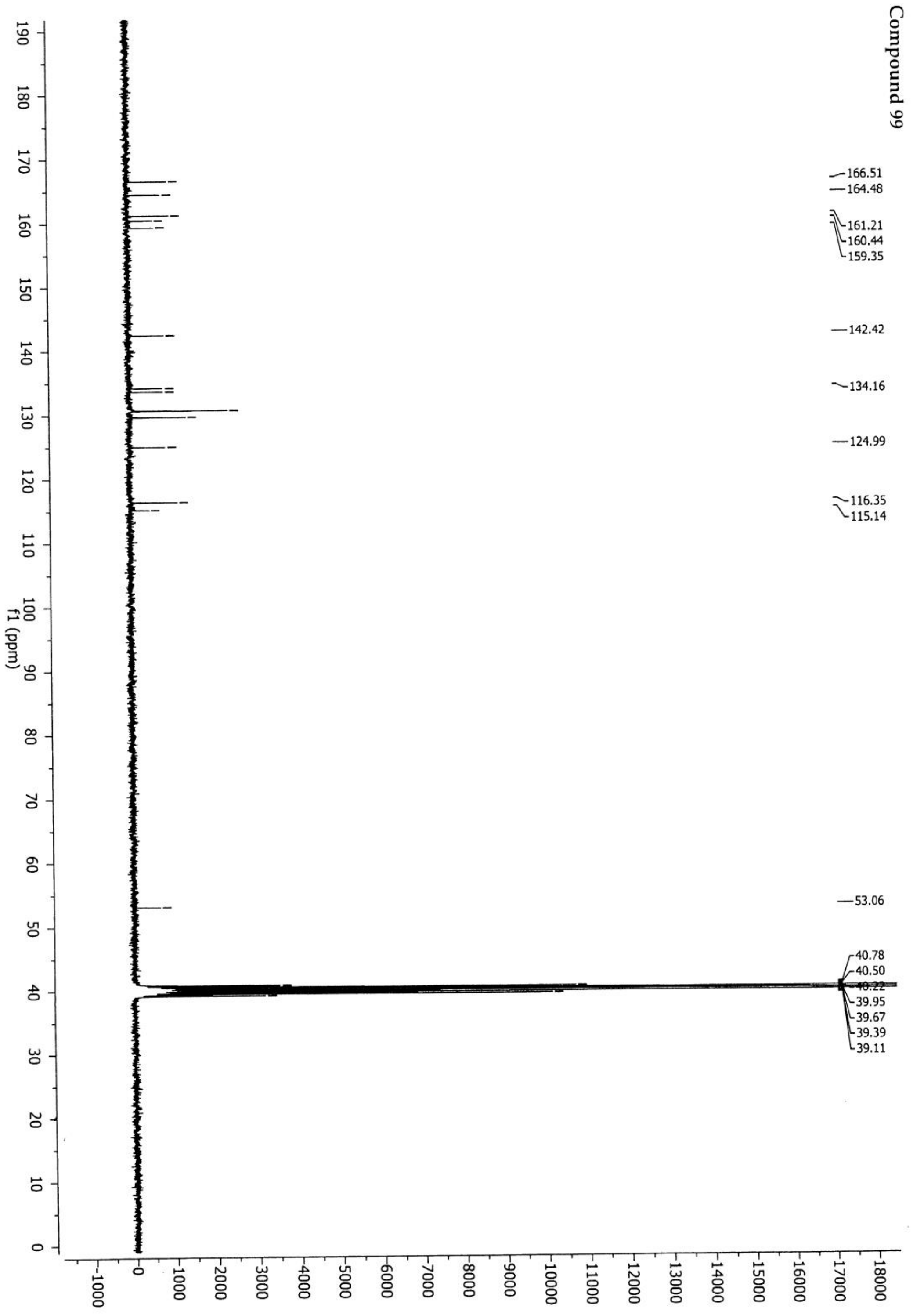

Fig. S106. ^{13}C NMR spectrum for compound **99**

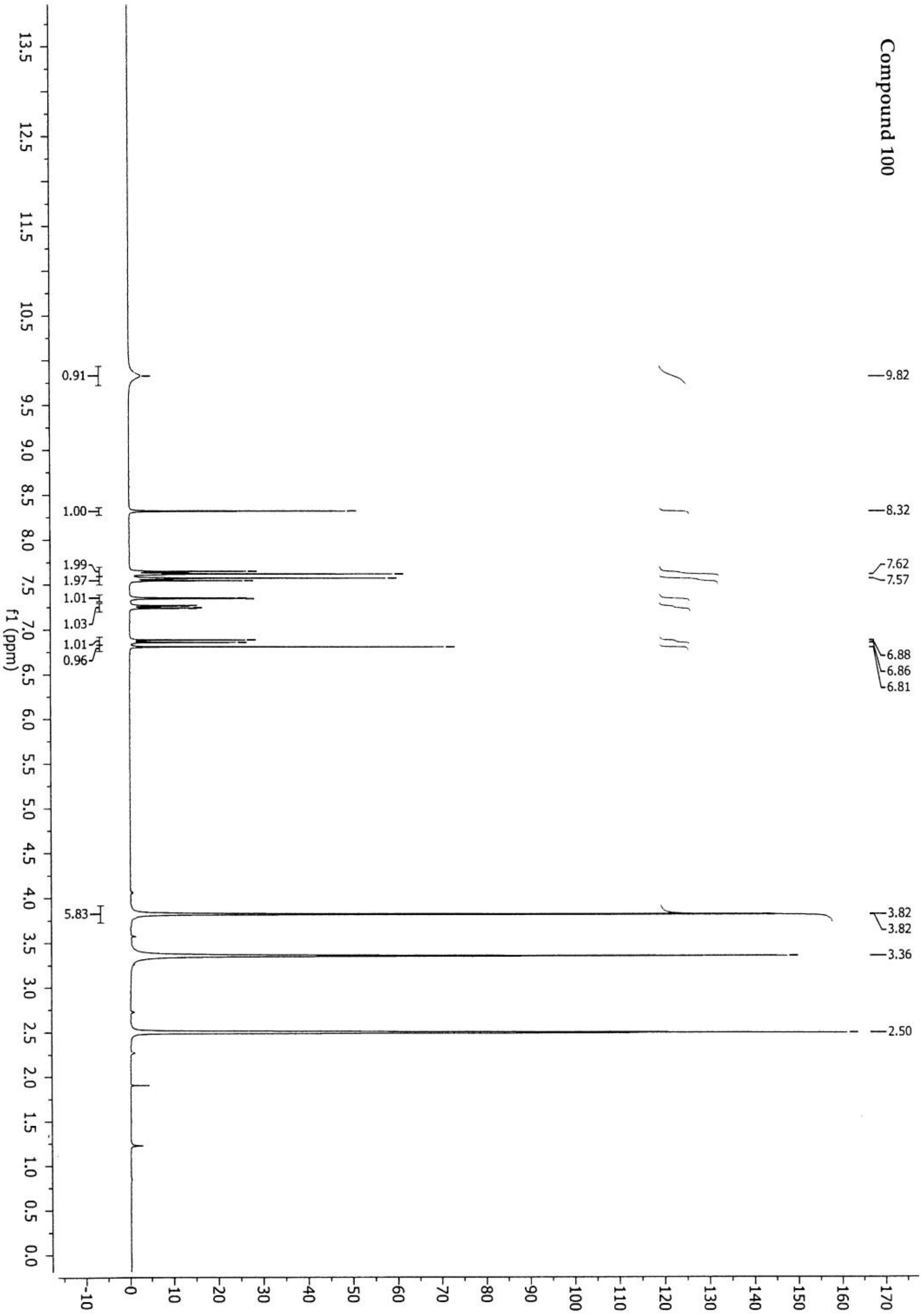

Fig. S107. ^{1}H NMR spectrum for compound **100**

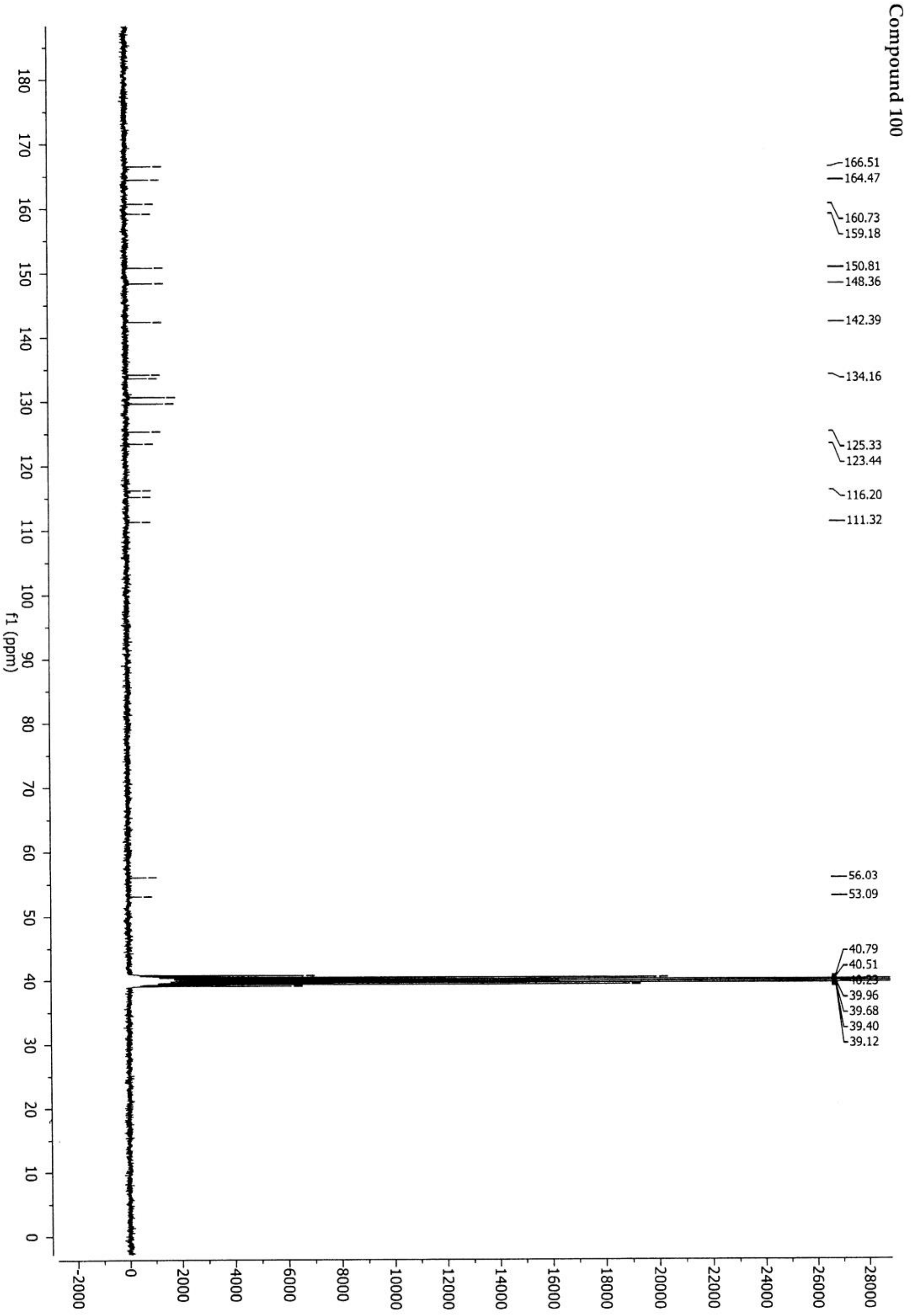

Fig. S108. ^{13}C NMR spectrum for compound **100**

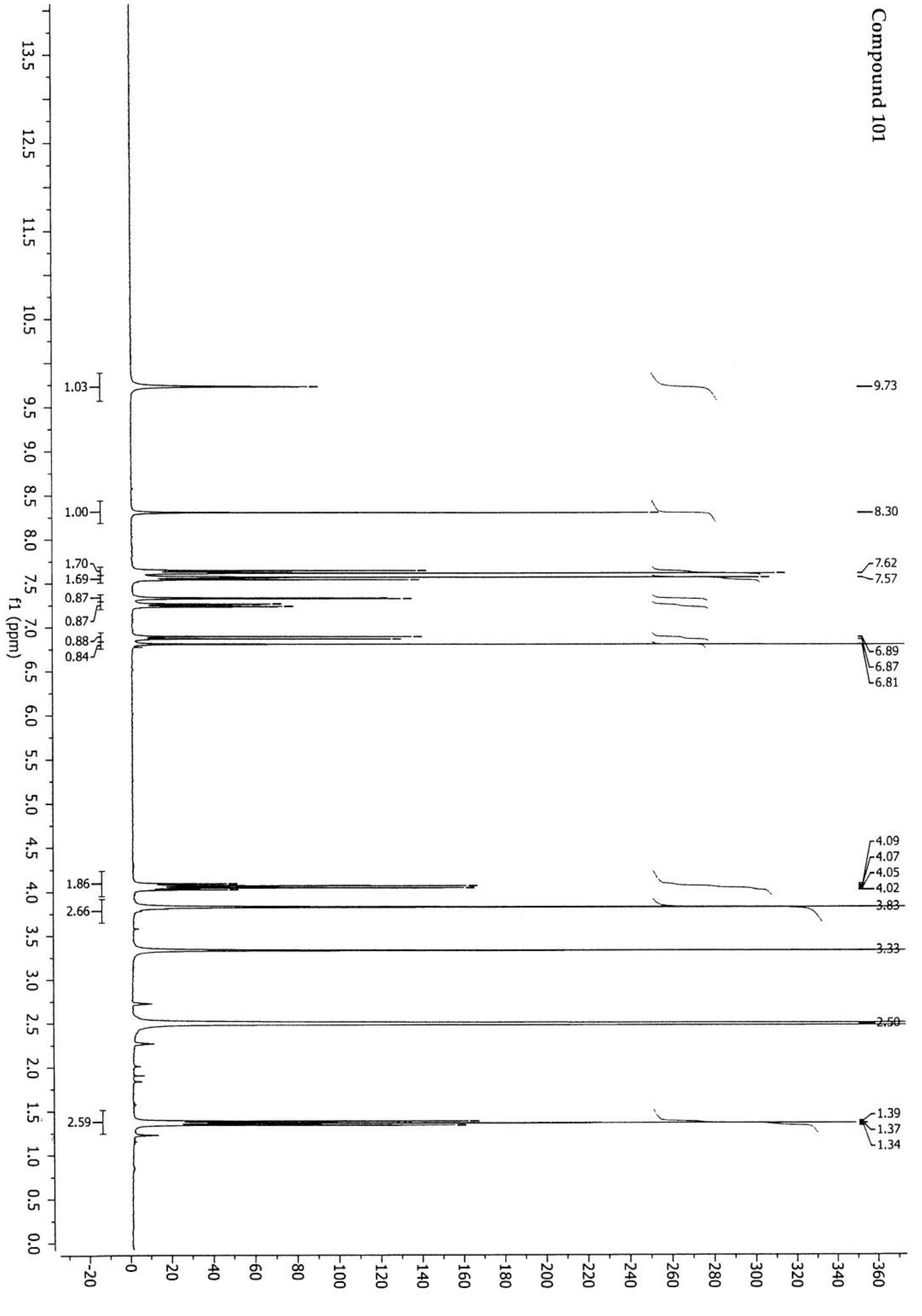

Fig. S109. ^{1}H NMR spectrum for compound **101**

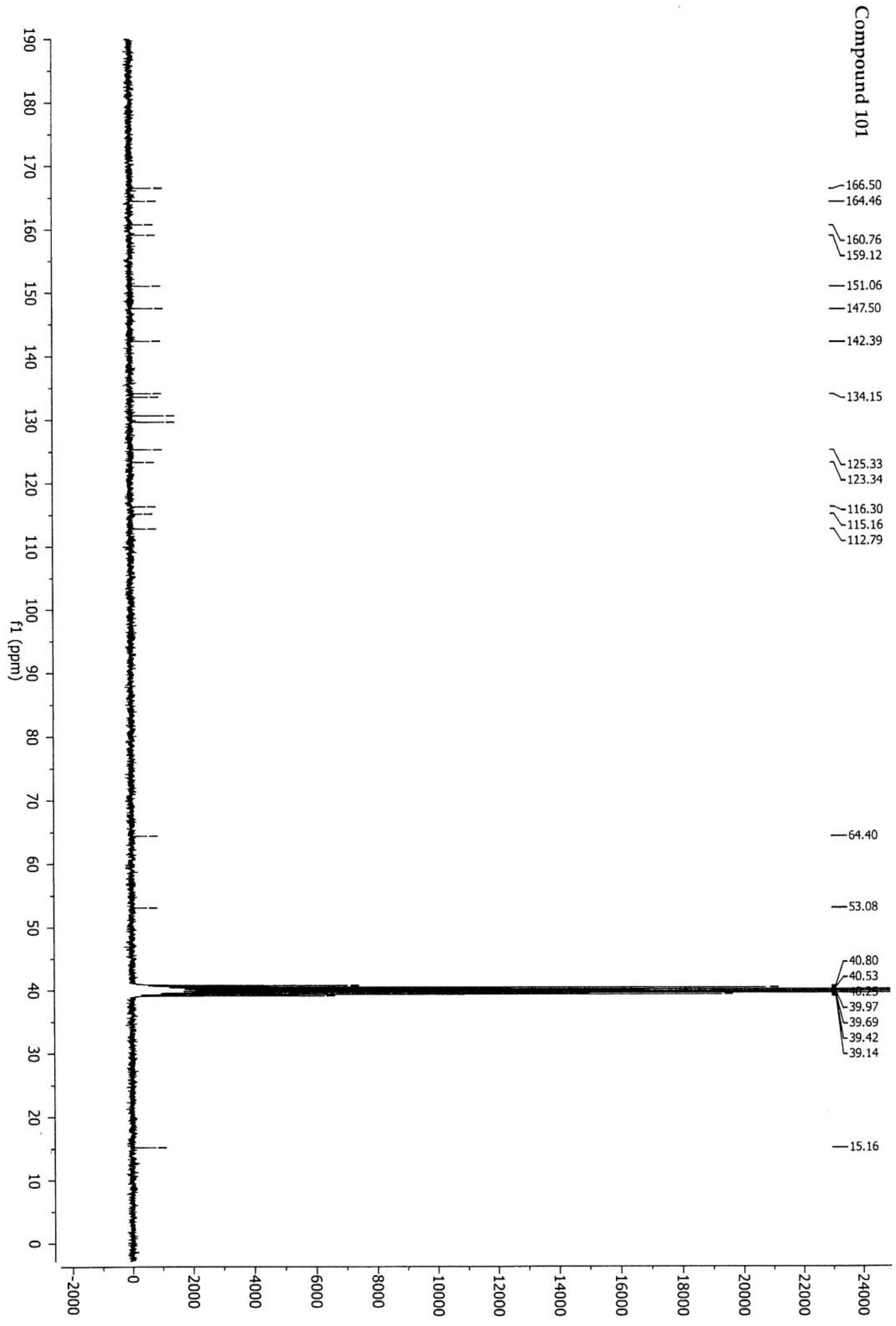

Fig. S110. ^{13}C NMR spectrum for compound **101**

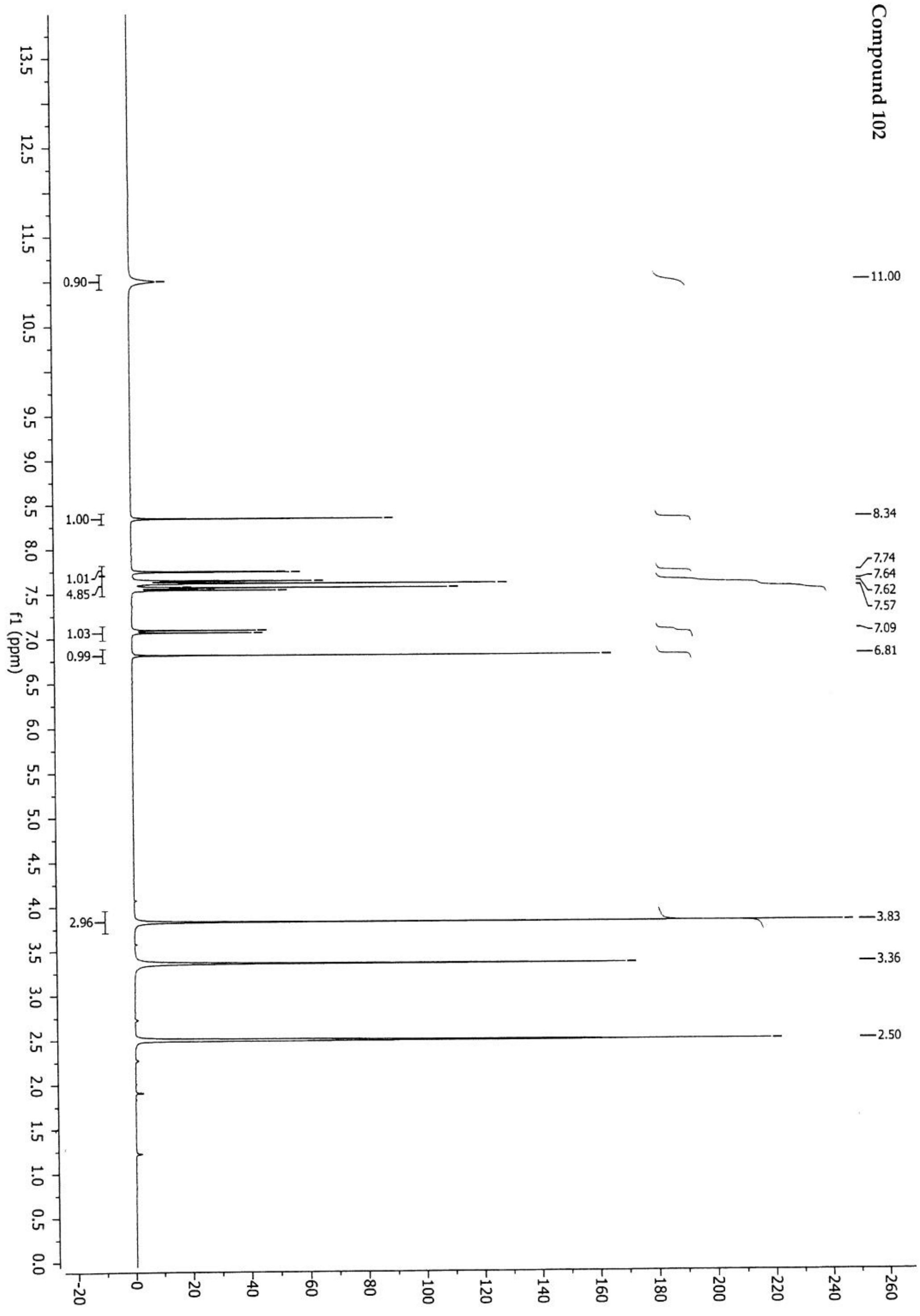

Fig. S111. ^{1}H NMR spectrum for compound **102**

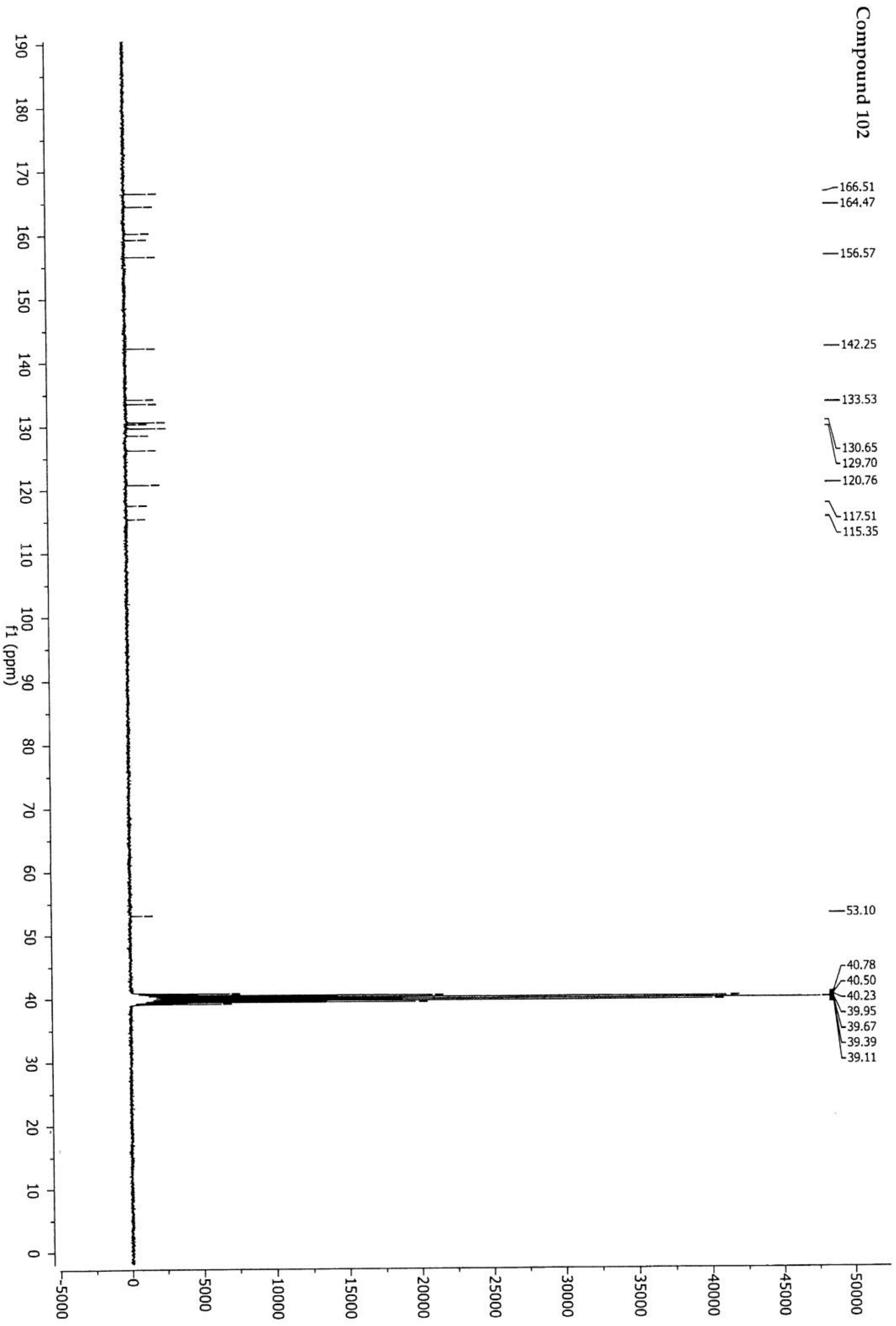

Fig. S112. ^{13}C NMR spectrum for compound **102**

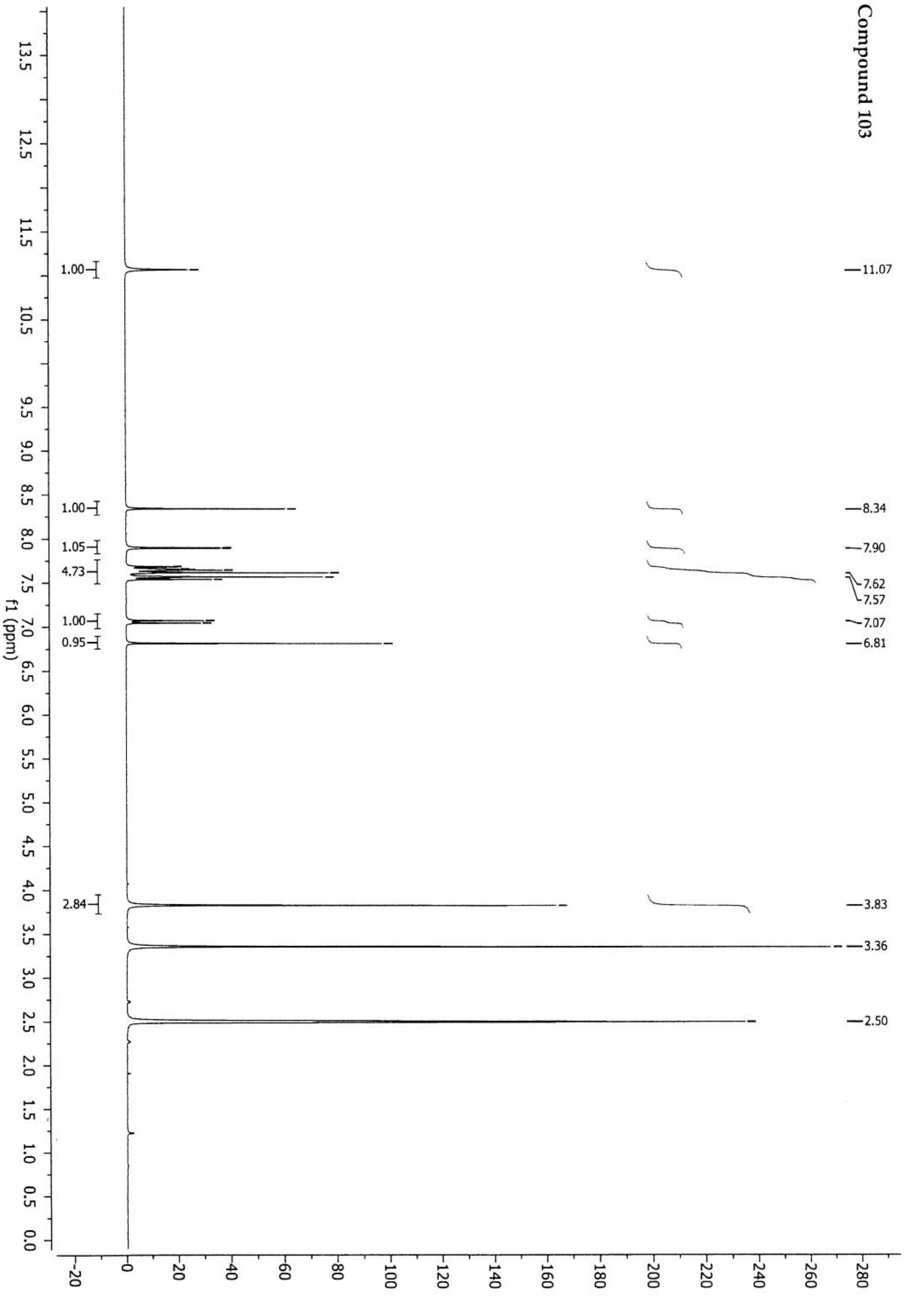

Fig. S113. ^{1}H NMR spectrum for compound **103**

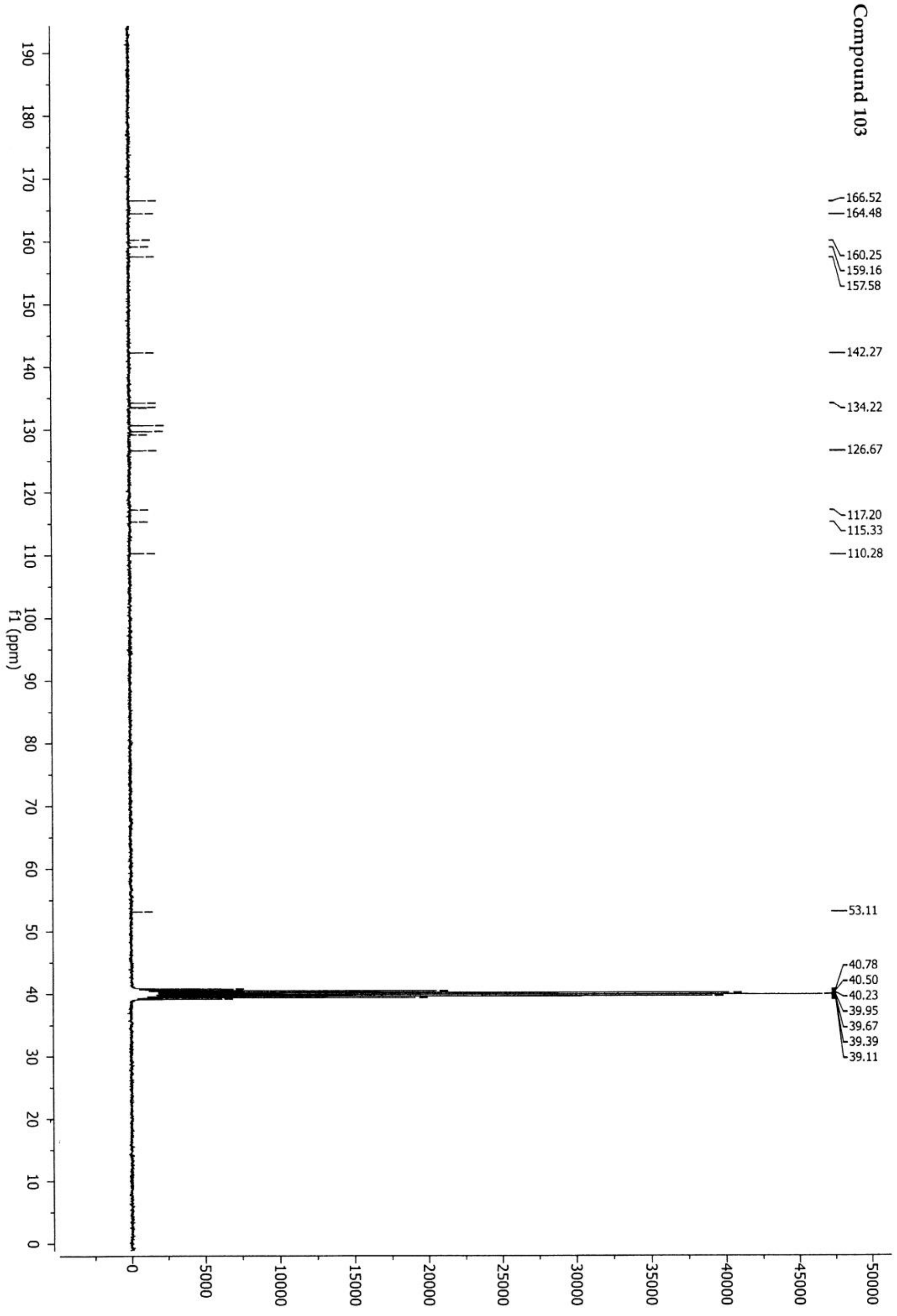

Fig. S114. ^{13}C NMR spectrum for compound **103**

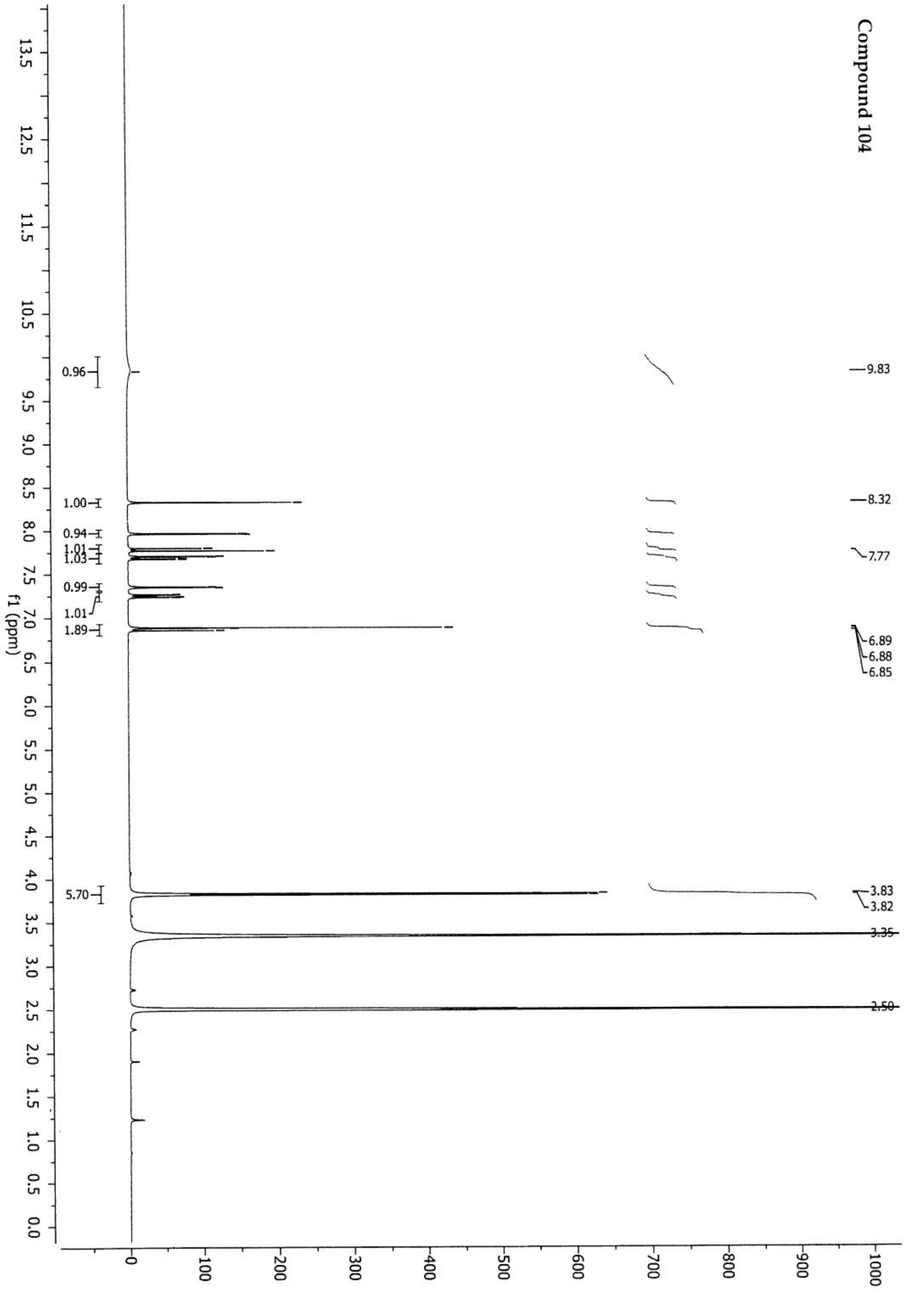

Fig. S115. ^{1}H NMR spectrum for compound **104**

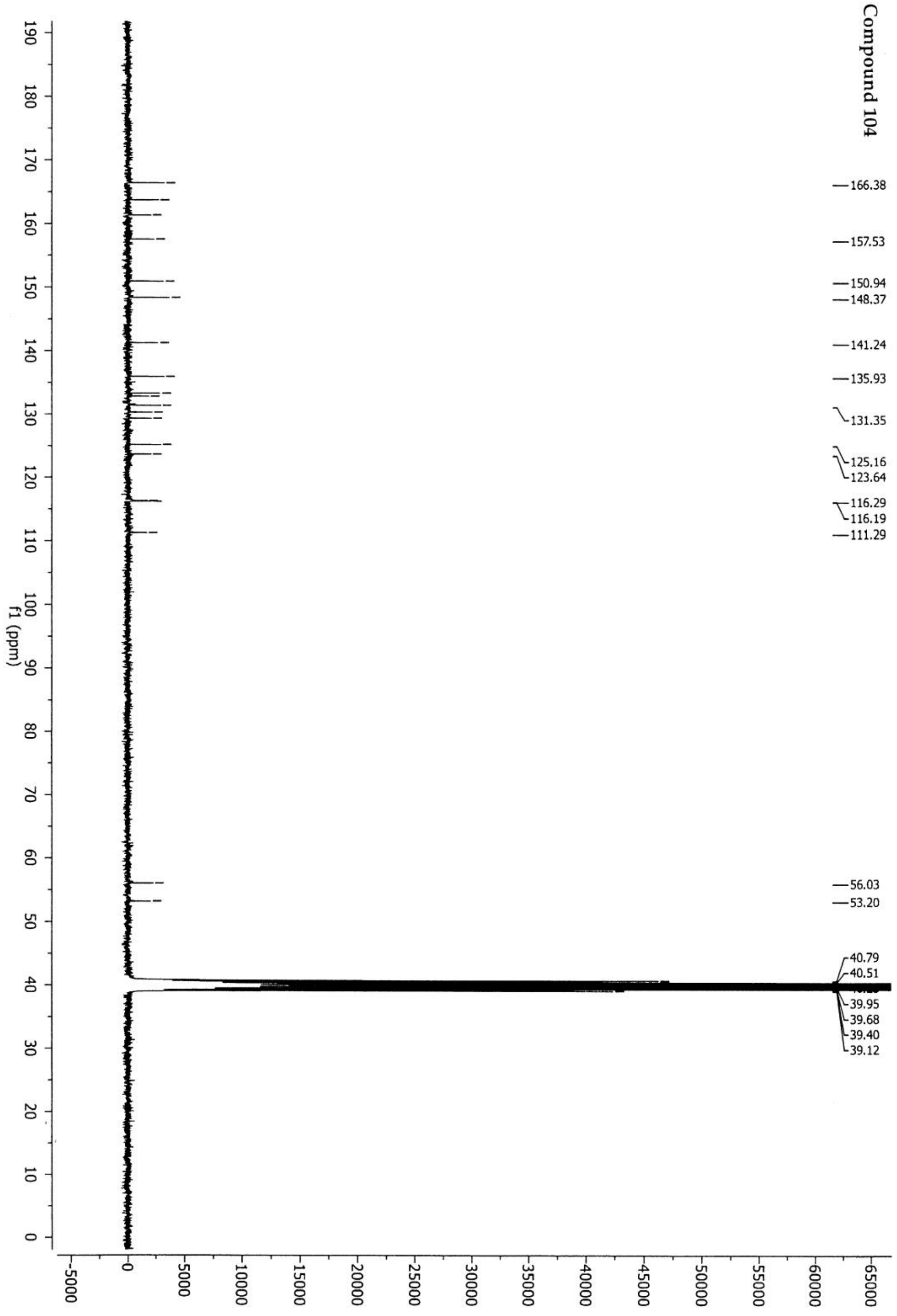

Fig. S116. ^{13}C NMR spectrum for compound **104**
